# A Realization Approach to Lossy Network Compression of a Tuple of Correlated Multivariate Gaussian RVs †

**DOI:** 10.3390/e24091227

**Published:** 2022-09-01

**Authors:** Charalambos D. Charalambous, Jan H. van Schuppen

**Affiliations:** 1 Department of Electrical and Computer Engineering, University of Cyprus, P.O. Box 20537, CY-1678 Nicosia, Cyprus; 2 Van Schuppen Control Research, Gouden Leeuw 143, 1103 KB Amsterdam, The Netherlands

**Keywords:** Gray–Wyner network, Wyner’s lossy common information, weak realizations of conditional independence, canonical variable form of multivariate Gaussian random variables, multi-user communication

## Abstract

Examined in this paper is the Gray and Wyner source coding for a simple network of correlated multivariate Gaussian random variables, $Y_{1}:\Omega \to R^{p_{1}}$ and $Y_{2}:\Omega \to R^{p_{2}}$. The network consists of an encoder that produces two private rates $R_{1}$ and $R_{2}$, and a common rate $R_{0}$, and two decoders, where decoder 1 receives rates $\left(R_{1},R_{0}\right)$ and reproduces $Y_{1}$ by ${\hat{Y}}_{1}$, and decoder 2 receives rates $\left(R_{2},R_{0}\right)$ and reproduces $Y_{2}$ by ${\hat{Y}}_{2}$, with mean-square error distortions $E||Y_{i}-{\hat{Y}}_{i}{\left|\right|}_{R^{p_{i}}}^{2}\leq \Delta_{i}\in \left[0,\infty \right],i=1,2$. Use is made of the weak stochastic realization and the geometric approach of such random variables to derive test channel distributions, which characterize the rates that lie on the Gray and Wyner rate region. Specific new results include: (1) A proof that, among all continuous or finite-valued random variables, W:Ω→W, Wyner’s common information, $C\left(Y_{1},Y_{2}\right)=\inf_{P_{Y_{1},Y_{2},W}:\;P_{Y_{1},Y_{2}|W}=P_{Y_{1}|W}P_{Y_{2}|W}}I\left(Y_{1},Y_{2};W\right)$, is achieved by a Gaussian random variable, $W:\Omega \to R^{n}$ of minimum dimension *n*, which makes the two components of the tuple $\left(Y_{1},Y_{2}\right)$ conditionally independent according to the weak stochastic realization of $\left(Y_{1},Y_{2}\right)$, and a the formula $C\left(Y_{1},Y_{2}\right)=\frac{1}{2}\sum_{j=1}^{n}\ln\left(\frac{1+d_{j}}{1-d_{j}}\right),$ where $d_{i}\in \left(0,1\right),i=1,\ldots ,n$ are the *canonical correlation coefficients* of the correlated parts of $Y_{1}$ and $Y_{2}$, and a realization of $\left(Y_{1},Y_{2},W\right)$ which achieves this. (2) The parameterization of rates that lie on the Gray and Wyner rate region, and several of its subsets. The discussion is largely self-contained and proceeds from first principles, while connections to prior literature is discussed.

## 1. Introduction

In their seminal paper, *Source Coding for a Simple Network* [1], Gray and Wyner characterized the lossless rate region for a tuple of finite-valued random variables, and the lossy rate region for a tuple of arbitrary distributed random variables. Many extensions and generalizations followed Gray and Wyner’s fundamental work. Wyner [2] introduced an operational definition of the common information between a tuple of sources that generate symbols with values in finite spaces. Wyner’s operational definition of common information is defined as the minimum achievable common message rate on the Gray and Wyner lossless rate region. Witsenhausen [3] investigated bounds for Wyner’s common information, and sequences of pairs of random variables in this regard [4]. Gács and Körner [5] introduced another definition of common randomness between a tuple of jointly independent and identically distributed random variables. Benammar and Zaidi [6,7] characterized the Gray–Wyner rate region, when there is side information at the decoders, under various scenarios that include both receivers and reproduce the source symbols without distortion. Insightful application examples for binary sources are considered in [7] (Section 4.2). In their previous work, Benammar and Zaidi [8,9] characterized the rate distortion function of the Heegard and Berger [10] problem, with two sources and side information at the two decoders (under a degraded set-up). Connections between the Gray and Wyner lossy source coding network and the notions of empirical and strong coordination capacity for arbitrary networks were developed by Cuff, Permuter and Cover [11] and the references therein, where the authors elaborated on the usefulness of the common information between the different network nodes.

Viswanatha, Akyol and Rose [12], and Xu, Liu and Chen [13], explored the connection of Wyner’s common information and the Gray and Wyner lossy rate region, to generalize Wyner’s common information to its lossy counterpart, for random variables taking values in arbitrary spaces. They characterized Wyner’s lossy common information as the minimum common message rate on the Gray and Wyner lossy rate region, when the sum rate is arbitrarily close to the rate distortion function with joint decoding for the Gray and Wyner lossy network. Applications to encryption and secret key generation are discussed by Viswanatha, Akyol and Rose in [12] (and references therein).

The current paper is focused on the calculations of rates that lie in the Gray and Wyner rate region [1], for two sources that generate symbols, according to the model of jointly independent and identically distributed multivariate correlated Gaussian random variables $Y_{1}:\Omega \to R^{p_{1}},Y_{2}:\Omega \to R^{p_{2}}$, and square-error fidelity at the two decoders. The current literature on methods and algorithms to compute such rates are subject to a number of limitations which often prevent their practical usefulness:

(1) Rates that lie in the Gray and Wyner rate region are only known for the special case of a tuple of scalar-valued Gaussian random variables with square error distortion, i.e., $p_{1}=p_{2}=1$ [1,12,13].

(2) Wyner’s lossy common information is only computed in closed form, for the special cases of a tuple of scalar-valued Gaussian random variables, [12,13].

(3) Important generalizations to a tuple of sources that generate multivariate Gaussian symbols, require new derivations often of considerable difficulty.

(4) Realizations of the optimal test channel distributions and their structural properties of the various rate distortion functions (RDFs), which are involved in the Gray and Wyner characterization of the rate region, are not developed.

(5) A proof that the Gray and Wyner for jointly Gaussian sources, is characterized by a Gaussian auxiliary random variable *W* is still missing from past literature.

It is known from [1] that the Gray and Wyner rate region can be parameterized by an *auxiliary random variable* W:Ω→W, via several rate distortion functions. Moreover, subsets of the Gray and Wyner rate region are parameterized by *W* which satisfies conditional independence (1).

(1) $$\begin{array}{c} P_{Y_{1},Y_{2}|W}=P_{Y_{1}|W}P_{Y_{2}|W}. \end{array}$$

The current paper makes use of the canonical variable form and the weak stochastic realization of the tuple of random variables $\left(Y_{1},Y_{2}\right)$, introduced in Section 2 to characterize subsets of the Gray and Wyner rate region, which are parameterized by jointly Gaussian random variables $\left(Y_{1},Y_{2},W\right)$ with $W:\Omega \to R^{n}$, where *n* is a finite number, while in some cases, the minimum dimension of *W* is clarified. The weak stochastic realization is developed to deal with the fundamental issue that, for the Gray and Wyner network, one is given the joint distribution $P_{Y_{1},Y_{2}}$, while the characterization of the RDFs involves the specification of the test channel distributions, that achieve these RDFs, and the actual construction of realizations of all random variables involved, that induce the test channel distributions. Furthermore, Wyner’s common information between $Y_{1}$ and $Y_{2}$ involves the construction of a joint distribution $P_{Y_{1},Y_{2},W}$ where *W* is the auxiliary random variable that makes $Y_{1}$ and $Y_{2}$ conditionally independent, i.e., (1) holds.

The rest of the section serves mainly to review the Gray and Wyner characterization of the lossy rate region and the characterization of Wyner’s lossy common information.

### 1.1. Literature Review

*(a) The Gray and Wyner source coding for a simple network [1].*

**Figure 1:**
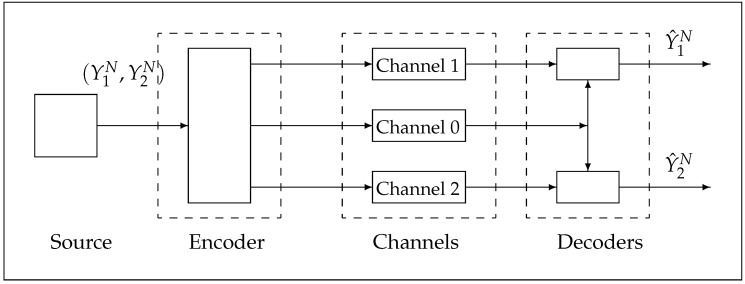
The Gray and Wyner source coding for a simple network [1] $\left(Y_{1,i},Y_{2,i}\right)∼P_{Y_{1},Y_{2}},i=1,\ldots ,N$.

Consider the Gray and Wyner source coding for a simple network, as shown in Figure 1, for a tuple of jointly independent and identically distributed multivariate Gaussian random variables $\left(Y_{1}^{N},Y_{2}^{N}\right)=\left\{\left(Y_{1,i},Y_{2,i}\right):i=1,2,\ldots ,N\right\}$,

(2) $$\begin{array}{c} Y_{1,i}:\Omega \to R^{p_{1}}=Y_{1},\;Y_{2,i}:\Omega \to R^{p_{2}}=Y_{2},\;i=1,\ldots ,N \end{array}$$

with square error distortion functions at the two decoders,

(3) $$\begin{array}{c} D_{Y_{1}}\left(y_{1}^{N},{\hat{y}}_{1}^{N}\right)=\frac{1}{N}\sum_{i=1}^{N}\left|\right|y_{1,i}-{\hat{y}}_{1,i}{\left|\right|}_{R^{p_{1}}}^{2},\;D_{Y_{2}}\left(y_{2}^{N},{\hat{y}}_{2}^{N}\right)=\frac{1}{N}\sum_{i=1}^{N}\left|\right|y_{2,i}-{\hat{y}}_{2,i}{\left|\right|}_{R^{p_{2}}}^{2} \end{array}$$

where $\left|\right|\cdot {\left|\right|}_{R^{p_{i}}}^{2}$ are Euclidean distances on $R^{p_{i}},i=1,2$.

The encoder takes as its input the data sequences $\left(Y_{1}^{N},Y_{2}^{N}\right)$ and produces at its output three messages, $\left(S_{0},S_{1},S_{2}\right)$, with binary bit representations $\left(NR_{0},NR_{1},NR_{2}\right)$, respectively. There are three channels, channel 0, channel 1, channel 2, with capacities $\left(C_{0},C_{1},C_{2}\right)$ (in bits per second), respectively, to transmit the messages to two decoders. Channel 0 is a common channel and channel 1 and channel 2 are the private channels which connect the encoder to each of the two decoders. Message $S_{0}$ is a *common* or *public* message that is transmitted through the common channel 0 with capacity $C_{0}$ to decoder 1 and decoder 2; $S_{1}$ is a *private* message which is transmitted through the *private* channel 1 with capacity $C_{1}$ to decoder 1; and $S_{2}$ is a *private* message, which is transmitted through the *private* channel 2 with capacity $C_{2}$ to decoder 2.

Decoder 1 aims to reproduce $Y_{1}^{N}$ by ${\hat{Y}}_{1}^{N}$ subject to an average distortion and decoder 2 aims to reproduce $Y_{2}^{N}$ by ${\hat{Y}}_{2}^{N}$, subject to an average distortion, where $\left({\hat{Y}}_{1,i},{\hat{Y}}_{2,i}\right)=\left({\hat{y}}_{1,i},{\hat{y}}_{2,i}\right)\in {\hat{Y}}_{1}\times {\hat{Y}}_{2}\subseteq Y_{1}\times Y_{2},i=1,\ldots ,N$, that is,

(4) $$\begin{array}{c} E\left\{D_{Y_{1}}\left(Y_{1}^{N},{\hat{Y}}_{1}^{N}\right)\right\}\leq \Delta_{1},\;E\left\{D_{Y_{2}}\left(Y_{2}^{N},{\hat{Y}}_{2}^{N}\right)\right\}\leq \Delta_{2},\;\left(\Delta_{1},\Delta_{2}\right)\in \left[0,\infty \right]\times \left[0,\infty \right]. \end{array}$$

Gray and Wyner characterized the rate region, denoted by $R_{GW}\left(\Delta_{1},\Delta_{2}\right)$, via a coding scheme that uses the auxiliary random variable *W*, and the family of probability distributions

(5) $$\begin{array}{c} P≜\left\{P_{Y_{1},Y_{2},W}\left(y_{1},y_{2},w\right),\;\;y_{1}\in Y_{1},y_{2}\in Y_{2},w\in W|\;\;P_{Y_{1},Y_{2},W}\left(y_{1},y_{2},\infty \right)=P_{Y_{1},Y_{2}}\left(y_{1},y_{2}\right)\right\} \end{array}$$

such that the joint probability distribution $P_{Y_{1},Y_{2},W}\left(y_{1},y_{2},w\right)$ on $Y_{1}\times Y_{2}\times W$, has a $\left(Y_{1},Y_{2}\right)$−marginal probability distribution $P_{Y_{1},Y_{2}}\left(y_{1},y_{2}\right)$ on $Y_{1}\times Y_{2}$ that coincides with the probability distribution of $\left(Y_{1},Y_{2}\right)$.

For the source and distortion function specified in (2) and (3), we apply the weak stochastic realization to construct the family of distributions P, which is parameterized by the auxiliary random variable *W*. Use is made of the characterization of $R_{GW}\left(\Delta_{1},\Delta_{2}\right)$ is described in terms of an auxiliary random variable, as follows.

**Theorem** **1** (Theorem 8 in [1]). *Let $R_{GW}\left(\Delta_{1},\Delta_{2}\right)$ denote the Gray and Wyner rate region of the simple network shown in Figure 1.*

*Suppose there exists ${\hat{y}}_{i}\in {\hat{Y}}_{i}$ such that $E\{d_{Y_{i}}\left(Y_{i},{\hat{y}}_{i}\right)\}<\infty$, for i=1,2.*

*For each $P_{Y_{1},Y_{2},W}\in P$ and $\Delta_{1}\geq 0,\Delta_{2}\geq 0$, define the subset of Euclidean 3− dimensional space*

(6) $$\begin{array}{c} R_{GW}^{P_{Y_{1},Y_{2},W}}\left(\Delta_{1},\Delta_{2}\right)=\left\{\left(R_{0},R_{1},R_{2}\right)|\;\;R_{0}\geq I\left(Y_{1},Y_{2};W\right),\;\;R_{1}\geq R_{Y_{1}|W}\left(\Delta_{1}\right),\;\;R_{2}\geq R_{Y_{2}|W}\left(\Delta_{2}\right)\right\} \end{array}$$

*where $R_{Y_{i}|W}\left(\Delta_{i}\right)$ is the conditional rate distortion function of $Y_{i}^{N}$, conditioned on $W^{N}$, at decoder i, for i=1,2, and $R_{Y_{1},Y_{2}}\left(\Delta_{1},\Delta_{2}\right)$ is the joint rate distortion function of the joint decoding of $\left(Y_{1}^{N},Y_{2}^{N}\right)$ (all single letters). Let*

(7) $$\begin{array}{c} R_{GW}^{*}\left(\Delta_{1},\Delta_{2}\right)={\left(\underset{P_{Y_{1},Y_{2},W}\in P}{⋃}R_{GW}^{P_{Y_{1},Y_{2},W}}\left(\Delta_{1},\Delta_{2}\right)\right)}^{c} \end{array}$$

*where ${\left(\cdot \right)}^{c}$ denotes the closure of the indicated set. The achievable Gray–Wyner lossy rate region is given by*

(8) $$\begin{array}{c} R_{GW}\left(\Delta_{1},\Delta_{2}\right)=R_{GW}^{*}\left(\Delta_{1},\Delta_{2}\right). \end{array}$$

Gray and Wyner [1] (Theorem 6) also showed that, if $\left(R_{0},R_{1},R_{2}\right)\in R_{GW}\left(\Delta_{1},\Delta_{2}\right)$, then

(9) $$\begin{array}{cc} R_{0}+R_{1}+R_{2} & \geq R_{Y_{1},Y_{2}}\left(\Delta_{1},\Delta_{2}\right), \end{array}$$

(10) $$\begin{array}{cc} R_{0}+R_{1} & \geq R_{Y_{1}}\left(\Delta_{1}\right), \end{array}$$

(11) $$\begin{array}{cc} R_{0}+R_{2} & \geq R_{Y_{2}}\left(\Delta_{2}\right) \end{array}$$

where $R_{Y_{i}}\left(\Delta_{i}\right)$ is the rate distortion function of $Y_{i}^{N}$ at decoder *i*, for i=1,2, and $R_{Y_{1},Y_{2}}\left(\Delta_{1},\Delta_{2}\right)$ is the joint rate distortion function of $\left(Y_{1}^{N},Y_{2}^{N}\right)$ at the two decoders. The inequality in (9) is called the *Pangloss Bound* of the Gray–Wyner lossy rate region $R_{GW}\left(\Delta_{1},\Delta_{2}\right)$. The set of triples $\left(R_{0},R_{1},R_{2}\right)\in R_{GW}\left(\Delta_{1},\Delta_{2}\right)$ that satisfy the equality $R_{0}+R_{1}+R_{2}=R_{Y_{1},Y_{2}}\left(\Delta_{1},\Delta_{2}\right)$ is called the *Pangloss Plane* of the Gray–Wyner lossy rate region $R_{GW}\left(\Delta_{1},\Delta_{2}\right)$.

Gray and Wyner proved that $R_{GW}\left(\Delta_{1},\Delta_{2}\right)$, is also determined from [1] ((4) of page 1703, Equation (42)),

(12) $$\begin{array}{c} T\left(\alpha_{1},\alpha_{2}\right)=\underset{P_{Y_{1},Y_{2},W}\in P}{\inf}\left\{I\left(Y_{1},Y_{2};W\right)+\alpha_{1}R_{Y_{1}|W}\left(\Delta_{1}\right)+\alpha_{2}R_{Y_{2}|W}\left(\Delta_{2}\right)\right\} \end{array}$$

where $0\leq \alpha_{i}\leq 1,i=1,2,\alpha_{1}+\alpha_{2}\geq 1$, and where for each $P_{Y_{1},Y_{2},W}\in P$, the conditional distribution $P_{Y_{1},Y_{2}|W}$ is defined, from which follows the $Y_{i}-$ marginals $P_{Y_{i}|W},i=1,2$.

*(b) Wyner’s common Information of finite-valued random variables.*

Wyner [2] introduced an operational definition of the common information between a tuple of random variables $\left(Y_{1}^{N},Y_{2}^{N}\right)$ that takes values in finite spaces.

The *first approach* of Wyner’s operational definition of common information between sequences $Y_{1}^{N}$ and $Y_{2}^{N}$ is defined as the minimum achievable common message rate $R_{0}$ on the Gray–Wyner network of Figure 1.

Wyner’s single letter information theoretic characterization of the infimum of all achievable message rates $R_{0}$, called Wyner’s common information, is defined by

(13) $$\begin{array}{c} C\left(Y_{1},Y_{2}\right)=\underset{P_{Y_{1},Y_{2},W}:\;P_{Y_{1},Y_{2}|W}=P_{Y_{1}|W}P_{Y_{2}|W}}{\inf}I\left(Y_{1},Y_{2};W\right). \end{array}$$

Here, $P_{Y_{1},Y_{2},W}$ is any joint probability distribution on $Y_{1}\times Y_{2}\times W$ with $\left(Y_{1},Y_{2}\right)$−marginal $P_{Y_{1},Y_{2}}$, such that *W* makes $Y_{1}$ and $Y_{2}$ conditionally independent, that is $P_{Y_{1},Y_{2},W}\in P$.

*(c) Minimum common message rate and Wyner’s lossy common information for arbitrary random variables.*

Viswanatha, Akyol and Rose [12], and Xu, Liu and Chen [13] explored the connection of Wyner’s common information and the Gray–Wyner lossy rate region, to provide a new interpretation of Wyner’s common information to its lossy counterpart.

The following characterization was derived by Xu, Liu and Chen [13] (an equivalent characterization was also derived by Viswanatha, Akyol and Rose [12]).

**Theorem** **2** (Theorem 4 in [13]). *Suppose there exists ${\hat{y}}_{i}\in {\hat{Y}}_{i}$ such that $E\{d_{Y_{i}}\left(Y_{i},{\hat{y}}_{i}\right)\}<\infty$, for i=1,2.*

*Let $C_{GW}\left(Y_{1},Y_{2};\Delta_{1},\Delta_{2}\right)$ denote the minimum common message rate $R_{0}$ on the Gray–Wyner lossy rate region $R_{GW}\left(\Delta_{1},\Delta_{2}\right)$, with a sum rate not exceeding the joint rate distortion function, $\sum_{i=0}^{2}R_{i}\geq R_{Y_{1},Y_{2}}\left(\Delta_{1},\Delta_{2}\right)$, while satisfying the average distortions.*

*Then, $C_{GW}\left(Y_{1},Y_{2};\Delta_{1},\Delta_{2}\right)$ is characterized by the optimization problem*

(14) $$\begin{array}{c} C_{GW}\left(Y_{1},Y_{2};\Delta_{1},\Delta_{2}\right)=\inf\;I\left(Y_{1},Y_{2};W\right) \end{array}$$

*such that the following identity holds*

(15) $$\begin{array}{c} R_{Y_{1}|W}\left(\Delta_{1}\right)+R_{Y_{2}|W}\left(\Delta_{2}\right)+I\left(Y_{1},Y_{2};W\right)=R_{Y_{1},Y_{2}}\left(\Delta_{1},\Delta_{2}\right) \end{array}$$

*where the infimum is over all random variables W taking values in W, which parameterize the source distribution via $P_{Y_{1},Y_{2},W}$, having a $Y_{1}\times Y_{2}-$ marginal source distribution $P_{Y_{1},Y_{2}}$, and induce joint distributions $P_{W,Y_{1},Y_{2},{\hat{Y}}_{1},{\hat{Y}}_{2}}$ which satisfy the constraints.*

It is shown in [12,13] that there exists a distortion region such that $C_{GW}\left(Y_{1},Y_{2};\Delta_{1},\Delta_{2}\right)=C_{W}\left(Y_{1},Y_{2}\right)$, i.e., it is independent of the distortions $\left(\Delta_{1},\Delta_{2}\right)$, and $C_{W}\left(Y_{1},Y_{2}\right)=C\left(Y_{1},Y_{2}\right)$, i.e., it is equal to Wyner’s information theoretic characterization of common information between $Y_{1}$ and $Y_{2}$, defined by (13). However, their proofs that *W* is finite-dimensional Gaussian relies on the assumption that *W* is continuous-valued.

The next theorem is derived by Xu, Liu and Chen [13].

**Theorem** **3** (Theorem 5 in [13]). *Let $\left(Y_{1},Y_{2}\right)$ be a pair of random variables with distribution $P_{Y_{1},Y_{2}}$ on the alphabet space $Y_{1}\times Y_{2}$, where $Y_{1}$ and $Y_{2}$ are arbitrary measurable spaces that can be discrete or continuous.*

*Let W be any random variable achieving $C(Y_{1},Y_{2})$ defined by (13).*

*Let the reproduction alphabet ${\hat{Y}}_{1}=Y_{1}$, ${\hat{Y}}_{2}=Y_{2}$ and two per-letter distortion measures $d_{Y_{1}}\left(y_{1},{\hat{y}}_{1}\right),d_{Y_{2}}\left(y_{2},{\hat{y}}_{2}\right)$ satisfy*

(16) $$\begin{array}{c} d_{Y_{i}}\left(y_{i},{\hat{y}}_{i}\right)>d_{Y_{i}}\left(y_{i},y_{i}\right)=0,\;\;y_{i}\neq {\hat{y}}_{i},\;\;i=1,2 \end{array}$$

*If the following conditions are satisfied:*

*(1) For any $y_{1}\in Y_{1},y_{2}\in Y_{2}$ and w∈W, $P_{W|Y_{1},Y_{2}}>0$;*

*(2) There exists an ${\hat{y}}_{i}\in {\hat{Y}}_{i}$, such that*

(17) $$\begin{array}{c} E\left\{d_{Y_{i}}\left(Y_{i},{\hat{y}}_{i}\right)\right\}<\infty ,\;\;\;\;i=1,2 \end{array}$$

*then there exists a strictly positive vector $\gamma =\left(\gamma_{1},\gamma_{2}\right)\in \left(0,\infty \right)\times \left(0,\infty \right)$, such that, for $0\leq (\Delta_{1},\Delta_{2})\leq \gamma$,*

(18) $$\begin{array}{c} C_{GW}\left(Y_{1},Y_{2};\Delta_{1},\Delta_{2}\right)=C_{W}\left(Y_{1},Y_{2}\right)=C\left(Y_{1},Y_{2}\right). \end{array}$$

*Moreover, $C_{GW}\left(Y_{1},Y_{2};\Delta_{1},\Delta_{2}\right)$ is constant on $D_{W}=\left\{\left(\Delta_{1},\Delta_{2}\right)\in \left[0,\infty \right]\times \left[0,\infty \right]:0\leq \left(\Delta_{1},\Delta_{2}\right)\leq \gamma \right\}$.*

The analog of the above theorem is also derived by Viswanatha, Akyol and Rose in [12] (Lemma 1). A subset of the Pangloss plane is derived by Gray and Wyner [1] (Theorem 9).

For a bivariate Gaussian random variables, i.e., $p_{1}=p_{2}=1$, with square-error distortions, Viswanatha, Akyol and Rose in [12], and Xu, Liu and Chen [13] computed $C_{GW}\left(Y_{1},Y_{2};\Delta_{1},\Delta_{2}\right)$, by using Xiao’s and Luo’s [14] (Theorem 6) the closed-form expression of joint rate distortion function $R_{Y_{1},Y_{2}}\left(\Delta_{1},\Delta_{2}\right)$. In addition, for the bivariate Gaussian random variables, with symmetric square-error distortions, i.e., $\Delta_{1}=\Delta_{2}=\Delta$, Gray and Wyner [1] (Section 2.5, (B)), computed a rate-triple $\left(R_{0},R_{1},R_{2}\right)\in R_{GW}\left(\Delta_{1},\Delta_{2}\right)$ that lies on the Pangloss plane.

### 1.2. Main Theorems and Discussion

What follows is a brief summary of the main theorems derived in this paper, and relations to the literature.

Theorem 9 shows that, among all joint distributions $P_{Y_{1},Y_{2},W}$ induced by a tuple of multivariate correlated Gaussian random variables $\left(Y_{1},Y_{2}\right)$, and an arbitrary random variable W:Ω→W, continuous or discrete-valued, Wyner’s common information $C(Y_{1},Y_{2})$, defined by (13), is minimized by a triple $\left(Y_{1},Y_{2},W\right)$ which induces a jointly Gaussian distribution $P_{Y_{1},Y_{2},W}$, and $W:\Omega \to W=R^{n}$ is a finite-dimensional Gaussian random variable. In particular, Theorem 9 gives the weak stochastic realization of $\left(Y_{1},Y_{2}\right)$, and the construction of the random variable *W*, which induce a joint distribution $P_{Y_{1},Y_{2},W}$ that achieves the minimum of $I(Y_{1},Y_{2};W)$ such that *W* makes $Y_{1}$ and $Y_{2}$ conditionally independent.

Then, use is made of Theorem 9, Section 2.2, such as Definition 1 of the canonical variable form and the weak stochastic realization to derive Wyner’s common information $C(Y_{1},Y_{2})$ defined by (13), and the optimal realization of the triple $\left(Y_{1},Y_{2},W^{*}\right)=\left(Y_{1},Y_{2},W^{*}\right)$ that achieves $C(Y_{1},Y_{2})$, as stated in the next theorem.

**Theorem** **4.** *Consider a tuple of Gaussian random variables $Y_{i}:\Omega \to R^{p_{i}}$, with $Q_{Y_{i}}>0$, for i=1,2, $\left(Y_{1},Y_{2}\right)\in G\left(0,Q_{\left(Y_{1},Y_{2}\right)}\right)$, $Q_{\left(Y_{1},Y_{2}\right)}\geq 0$, and apply Algorithm A1 (and the notation therein) to decompose and transform the random variables into a canonical variable form (with abuse of notation, the transform random variables are denoted by $\left(Y_{1},Y_{2}\right)\in G\left(0,Q_{\mathrm{cvf}}\right)$.), $\left(Y_{1},Y_{2}\right)\in G\left(0,Q_{\mathrm{cvf}}\right)$, using the material and notation of Section 2.2, i.e., Definition 1.*

*(a) Then,*

(19) $$\begin{array}{cc} C(Y_{1},Y_{2})= & C\left(Y_{11},Y_{21}\right)+C\left(Y_{12},Y_{22}\right)+C\left(Y_{13},Y_{23}\right)\\ = & \left\{\begin{array}{ccc} 0, & if & p_{13}>0,\;p_{23}>0,\;p_{11}=p_{12}=p_{21}=p_{22}=0,\\ \frac{1}{2}\sum_{i=1}^{n}\ln\left(\frac{1+d_{i}}{1-d_{i}}\right), & if & p_{12}=p_{22}>0,\;p_{11}=p_{21}=0,\;p_{13}\geq 0,\;p_{23}\geq 0,\\ +\infty , & if & p_{11}=p_{21}>0 \end{array}\right. \end{array}$$

*where $\left(p_{11},p_{12},p_{13}\right)$ and $\left(p_{21},p_{22},p_{23}\right)$ are the dimensions of the canonical variable decomposition of the tuple $\left(Y_{1},Y_{2}\right)$, and*

(20) $$\begin{array}{cc} C(Y_{11},Y_{21})= & +\infty ,\;\;if\;\;p_{11}=p_{21}>0; \end{array}$$

(21) $$\begin{array}{cc} C(Y_{13},Y_{23})= & 0,\;\;if\;\;p_{13}>0\;\;and\;\;p_{23}>0; \end{array}$$

(22) $$\begin{array}{cc} C(Y_{12},Y_{22})= & \frac{1}{2}\sum_{i=1}^{n}\ln\left(\frac{1+d_{i}}{1-d_{i}}\right),\;\;if\;\;n=p_{12}=p_{22}>0. \end{array}$$

*Thus, $C(Y_{12},Y_{22})$ is the most interesting value if defined.*

*(b) The random variable $W^{*}$ defined below is such that $C(Y_{1},Y_{2})$ of part (a) is attained.*

(23) $$\begin{array}{c} W^{*}:\Omega \to R^{n},\;\;n\in Z_{+},\\ n_{1}=p_{11}=p_{21},\;\;n_{2}=p_{12}=p_{22},\;\;n_{1}+n_{2}=n,\\ W^{*}=\left(\begin{array}{c} W_{1}^{*}\\ W_{2}^{*} \end{array}\right),\;\;\;W_{1}^{*}:\Omega \to R^{n_{1}},\;\;W_{2}^{*}:\Omega \to R^{n_{2}},\;\;\\ W_{1}^{*}=Y_{11}=Y_{21}, \end{array}$$

(24) $$W_{2}^{*}=L_{1}Y_{12}+L_{2}Y_{22}+L_{3}V,\;\;see\;Theorem\;11.\left(b\right)for\;the\;formulas\;of\;L_{1},\;L_{2},\;L_{3}$$

*where the following properties hold:*

(25) $$then\;\left(Y_{1},Y_{2},W^{*}\right)\in G\left(0,Q_{s}\left(I\right)\right),\;see\;\left(81\right)forQ_{s}\left(I\right),$$

(26) $$(F^{Y_{11},Y_{12},Y_{13}},F^{Y_{21},Y_{22},Y_{23}}|F^{W_{1}^{*},W_{2}^{*}})\in \mathrm{CI},$$

(27) $$F^{W_{1}^{*}}\subseteq \left(F^{Y_{11}}\vee F^{Y_{21}}\right),F^{W_{2}^{*}}\subseteq \left(F^{Y_{12}}\vee F^{Y_{22}}\right),$$

(28) $$C\left(Y_{1},Y_{2}\right)=I\left(Y_{1},Y_{2};W^{*}\right).$$

*(c) The following operations are defined, using (a),*

(29) $$W^{*}=\left(\begin{array}{c} W_{1}^{*}\\ W_{2}^{*} \end{array}\right),$$

(30) $$W_{1}^{*}=Y_{11}=Y_{21},$$

(31) $$W_{2}^{*}=L_{1}Y_{12}+L_{2}Y_{22}+L_{3}V,\;\;see\;(103),\;(104)\;for\;the\;formulas\;of\;L_{1},\;L_{2},\;L_{3};$$

(32) $$Z_{12}=Y_{12}-E\left[Y_{12}|F^{W_{2}^{*}}\right]=Y_{12}-Q_{Y_{12},W_{2}^{*}}Q_{W_{2}^{*}}^{-1}W_{2}^{*},\;$$

(33) $$Z_{22}=Y_{22}-E\left[Y_{22}|F^{W_{2}^{*}}\right]=Y_{22}-Q_{Y_{22},W_{2}^{*}}Q_{W_{2}^{*}}^{-1}W_{2}^{*},$$

(34) $$Z_{13}=Y_{13},\;\;Z_{23}=Y_{23},\;\;(the\;components\;Z_{11}\;and\;Z_{21}\;do\;not\;exist),$$

(35) $$Z_{1}=\left(\begin{array}{c} Z_{12}\\ Z_{13} \end{array}\right),\;Z_{2}=\left(\begin{array}{c} Z_{22}\\ Z_{23} \end{array}\right)$$

*and these imply,*

(36) $$Y_{11}=W_{1}^{*}=Y_{21},$$

(37) $$Y_{12}=Q_{Y_{12},W_{2}^{*}}Q_{W_{2}^{*}}^{-1}W_{2}^{*}+Z_{12},\;\;\;\;Y_{22}=Q_{Y_{22},W_{2}^{*}}Q_{W_{2}^{*}}^{-1}W_{2}^{*}+Z_{22},$$

(38) $$Y_{13}=Z_{13},\;\;\;\;Y_{23}=Z_{23};$$

*equivalently*

$$\begin{array}{c} \left(\begin{array}{c} Y_{11}\\ Y_{12}\\ Y_{13} \end{array}\right)=\left(\begin{array}{cc} I_{n_{1}} & 0\\ 0 & Q_{Y_{12},W_{2}^{*}}Q_{W_{2}^{*}}^{-1}\\ 0 & 0 \end{array}\right)\left(\begin{array}{c} W_{1}^{*}\\ W_{2}^{*} \end{array}\right)+\left(\begin{array}{cc} 0 & 0\\ I_{n_{2}} & 0\\ 0 & I_{n-n_{1}-n_{2}} \end{array}\right)\left(\begin{array}{c} Z_{12}\\ Z_{13} \end{array}\right),\\ \left(\begin{array}{c} Y_{21}\\ Y_{22}\\ Y_{23} \end{array}\right)=\left(\begin{array}{cc} I_{n_{1}} & 0\\ 0 & Q_{Y_{22},W_{2}^{*}}Q_{W_{2}^{*}}^{-1}\\ 0 & 0 \end{array}\right)\left(\begin{array}{c} W_{1}^{*}\\ W_{2}^{*} \end{array}\right)+\left(\begin{array}{cc} 0 & 0\\ I_{n_{2}} & 0\\ 0 & I_{n-n_{1}-n_{2}} \end{array}\right)\left(\begin{array}{c} Z_{22}\\ Z_{23} \end{array}\right). \end{array}$$

The derivation of Theorem 4 is presented in Section 3.2, after several of the tools are presented, such as, weak stochastic realizations and minimal realizations.

**Remark** **1.** *Relation of Theorem 4 to the literature.*

*(a) Corollary 1 in [15] gives an expression analogous to the case (20), which is expressed in terms of the correlation coefficients, $\rho_{i}\in \left(-1,1\right)$ and not the canonical correlation coefficients $d_{i}\in \left(0,1\right)$. Similarly, [16], under Lemma 1, reproduces Corollary 1 in [15], with the correlation coefficients $\rho_{i}$ replaced by their absolute values $|\rho_{i}|$.*

*(b) The derivation in [15,16] is based on the use of rate distortion functions of Gaussian random variables with square-error distortion functions, which presupposes the that auxiliary RV W→W takes continuous values.*

*(c) Refs. [15,16] do not provide a realization of the triple $\left(Y_{1},Y_{2},W^{*}\right)$, as given in Theorem 4 (which is based on applying the parametrization of Theorem 8).*

*On the other hand, the derivation of Theorem 4 is based on Theorem 9, which shows that, among all joint distributions $P_{Y_{1},Y_{2},W}$ induced by a tuple of multivariate correlated Gaussian random variables $\left(Y_{1},Y_{2}\right)$, and an arbitrary random variable W:Ω→W, continuous or discrete-valued, Wyner’s common information $C(Y_{1},Y_{2})$, defined by (13), is minimized by a triple $\left(Y_{1},Y_{2},W\right)$ which induces a jointly Gaussian distribution $P_{Y_{1},Y_{2},W}$, and $W:\Omega \to W=R^{n}$ is finite-dimensional Gaussian random variable.*

*(d) The derivation of Theorem 4 contains many intermediate results which are applicable to the problems considered in [15,16], such as Relaxed Wyner’s Common Information in [17]. These are discussed in Section 4.3.*

Theorem 5 gives a parametric characterization of the Gray and Wyner rate region $R_{GW}\left(\Delta_{1},\Delta_{2}\right)$, with respect to the variance matrix of the triple of jointly Gaussian random variables $\left(Y_{1},Y_{2},W\right)$.

**Theorem** **5.** *Consider a tuple of Gaussian random variables $Y_{i}:\Omega \to R^{p_{i}}$, with $Q_{Y_{i}}>0$, for i=1,2, $\left(Y_{1},Y_{2}\right)\in G\left(0,Q_{\left(Y_{1},Y_{2}\right)}\right)$ (not necessarily in canonical variable form), with induced Gaussian measure $P_{0}=G\left(0,Q_{\left(Y_{1},Y_{2}\right)}\right)$ on the space $\left(R^{p_{1}}\times R^{p_{2}},B\left(R^{p_{1}}\right)⊗B\left(R^{p_{2}}\right)\right)$, and square error distrortion functions $D_{Y_{1}}\left(y_{1},{\hat{y}}_{1}\right)=\left|\right|y_{1}-{\hat{y}}_{1}{\left|\right|}_{R^{p_{1}}}^{2},\;D_{Y_{2}}\left(y_{2},{\hat{y}}_{2}\right)=\left|\right|y_{2}-{\hat{y}}_{2}{\left|\right|}_{R^{p_{2}}}^{2}$.*

*The following hold.*

*(a) There exists a Gaussian measure $P_{1}=G\left(0,Q_{\left(Y_{1},Y_{2},W\right)}\right)$ defined on the space $\left(R^{p_{1}}\times R^{p_{2}}\times R^{n},B\left(R^{p_{1}}\right)⊗B\left(R^{p_{2}}\right)⊗B\left(R^{n}\right)\right),n\in Z_{+}$ associated with the Gaussian random variables $\left(Y_{1},Y_{2})\right)$, $W:\Omega \to R^{n},W\in G\left(0,Q_{W}\right)$ such that $P_{1}{|}_{R^{p_{1}}\times R^{p_{2}}}=G\left(0,Q_{\left(Y_{1},Y_{2}\right)}\right)$. Moreover, a realization of the random variables $\left(Y_{1},Y_{2},W\right)$ with induced measure $P_{1}=G\left(0,Q_{\left(Y_{1},Y_{2},W\right)}\right)$ is*

(39) $$\left(\begin{array}{c} Y_{1}\\ Y_{2} \end{array}\right)=Q_{(Y_{1},Y_{2}),W}Q_{W}^{†}W+\left(\begin{array}{c} Z_{1}\\ Z_{2} \end{array}\right)$$

(40) $$\left(Z_{1},Z_{2}\right)\in G\left(0,Q_{\left(Z_{1},Z_{2}\right)}\right),\;\;\left(Z_{1},Z_{2}\right)\;\;independent\;of\;\;W,$$

(41) $$Q_{(Y_{1},Y_{2}),W}=E\left[\left(\begin{array}{c} Y_{1}\\ Y_{2} \end{array}\right)W^{T}\right]=\left(\begin{array}{c} Q_{Y_{1},W}\\ Q_{Y_{2},W} \end{array}\right)$$

*where $Q_{W}^{†}$ is the pseudoinverse of $Q_{W}$.*

*(b) For Gaussian auxiliary random variables given in part (a), the Gray–Wyner rate region $R_{GW}\left(\Delta_{1},\Delta_{2}\right)$ is determined from*

$$\begin{array}{cc} T^{G}\left(\alpha_{1},\alpha_{2}\right)= & \underset{\left(Y_{1},Y_{2},W\right)\in G\left(0,Q_{\left(Y_{1},Y_{2},W\right)}\right)\;of(68)}{\inf}\{I\left(Y_{1},Y_{2};W\right) \end{array}$$

(42) $$\begin{array}{cc} \; & +\alpha_{1}R_{Y_{1}|W}\left(\Delta_{1}\right)+\alpha_{2}R_{Y_{2}|W}\left(\Delta_{2}\right)\}\\ = & \underset{\left(Y_{1},Y_{2},W\right)\in G\left(0,Q_{\left(Y_{1},Y_{2},W\right)}\right),\;of(),()}{\inf}\{I\left(Y_{1},Y_{2};W\right) \end{array}$$

(43) $$\begin{array}{cc} \; & +\alpha_{1}R_{Y_{1}|W}\left(\Delta_{1}\right)+\alpha_{2}R_{Y_{2}|W}\left(\Delta_{2}\right)\}\\ = & \underset{Q_{Y_{1},Y_{2}|W},Q_{Y_{i}|W},i=1,2}{\inf}\{\frac{1}{2}\ln{\left(\frac{\det(Q_{\left(Y_{1},Y_{2}\right)})}{\det\left(Q_{Y_{1}|Y_{2},W}\right)\det\left(Q_{Y_{2}|W}\right)}\right)}^{+} \end{array}$$

(44) $$+\alpha_{1}R_{Y_{1}|W}\left(\Delta_{1}\right)+\alpha_{2}R_{Y_{2}|W}\left(\Delta_{2}\right)\}$$

(45) $$\;subject\;to,\;\;Q_{\left(Y_{1},Y_{2}\right)}\geq Q_{(Y_{1},Y_{2})|W},\;\;Q_{Y_{1}|Y_{2},W}=Q_{Y_{1}|W}-Q_{Y_{1},Y_{2}|W}Q_{Y_{2}|W}^{†}Q_{Y_{1},Y_{2}|W}^{T}$$

*where $0\leq \alpha_{i}\leq 1,i=1,2,\alpha_{1}+\alpha_{2}\geq 1$, $I\left(Y_{1},Y_{2};W\right)=H\left(Y_{1},Y_{2}\right)-H\left(Y_{1}|Y_{2},W\right)-H\left(Y_{2}|W\right)$, and $R_{Y_{i}|W}\left(\Delta_{i}\right),i=1,2$ are given in Theorem 13.(b), and ${\left(\cdot \right)}^{+}=\max\left\{1,\cdot \right\}$.*

The derivation of Theorem 5 is presented in Section 4.4, after the structural properties of RDFs, $R_{Y_{1},Y_{2}}\left(\Delta_{1},\Delta_{2}\right),R_{Y_{i}|W}\left(\Delta_{i}\right),R_{Y_{i}}\left(\Delta_{i}\right),i=1,2$, of Theorem 12, Theorem 13, Theorem 14 are presented. From Theorem 5, follow simplified characterizations of subsets of the rate region $R_{GW}\left(\Delta_{1},\Delta_{2}\right)$, such as rates that lie on Pangloss Plane, and rates that correspond to *W* that make $Y_{1}$ and $Y_{2}$ conditional independent, i.e., *W* is such that $P_{Y_{1},Y_{2}|W}=P_{Y_{1}|W}P_{Y_{2}|W}$.

Utilizing the structural properties of RDFs, $R_{Y_{1},Y_{2}}\left(\Delta_{1},\Delta_{2}\right),R_{Y_{i}|W}\left(\Delta_{i}\right),R_{Y_{i}}\left(\Delta_{i}\right),i=1,2$, of Theorem 12, Theorem 13, Theorem 14, and Theorem 4, the next theorem is obtained, which gives the formula of Wyner’s lossy common information $C_{GW}\left(Y_{1},Y_{2};\Delta_{1},\Delta_{2}\right)=C_{W}\left(Y_{1},Y_{2}\right)$.

**Theorem** **6.** *Consider a tuple $\left(Y_{1},Y_{2}\right)$ of Gaussian random variables in the canonical variable form of Definition 1. Restrict attention to the correlated parts of these random variables, as defined in Theorem 8, by (77)–(79), and subset of the distortion region is defined by*

(46) $$D_{W}=\left\{\left(\Delta_{1},\Delta_{2}\right)\in \left[0,\infty \right]\times \left[0,\infty \right]|\;\;0\leq \Delta_{1}\leq n\left(1-d_{1}\right),\;\;0\leq \Delta_{2}\leq n\left(1-d_{1}\right)\right\},$$

(47) $$\forall j\in Z_{n},\;\;d_{j}\in \left(0,1\right).$$

*Then, Wyner’s lossy common information (calculation of expression in Theorem 3) is given by*

(48) $$\begin{array}{cc} C_{GW}\left(Y_{1},Y_{2};\Delta_{1},\Delta_{2}\right)= & \;C_{W}\left(Y_{1},Y_{2}\right) \end{array}$$

(49) $$\begin{array}{cc} = & \;C\left(Y_{1},Y_{2}\right)=\frac{1}{2}\sum_{j=1}^{n}\ln\left(\frac{1+d_{j}}{1-d_{j}}\right),\;\;\left(\Delta_{1},\Delta_{2}\right)\in D_{W} \end{array}$$

The derivation of Theorem 6 is presented in Section 4.2 and makes use of a degenerate version of the realization of the triple $\left(Y_{1},Y_{2},W^{*}\right)$ given in Theorem 4, and the RDFs $R_{Y_{1},Y_{2}}\left(\Delta_{1},\Delta_{2}\right),R_{Y_{i}|W}\left(\Delta_{i}\right),i=1,2$.

**Remark** **2.** *By Theorem 5, a subset of the Gray–Wyner rate region is obtained by replacing $\left(Y_{1},Y_{2},W\right)\in G\left(0,Q_{\left(Y_{1},Y_{2},W\right)}\right)$ of (39) and (40) by W that makes $Y_{1}$ and $Y_{2}$ conditionally independent, i.e., $\left(Z_{1},Z_{2}\right)\in G\left(0,Q_{\left(Z_{1},Z_{2}\right)}\right)$ and $\left(Z_{1},Z_{2},W\right)$ mutually independent (e.g., $Q_{\left(Z_{1},Z_{2}\right)}$ is block-diagonal).*

### 1.3. Structure of the Paper

Section 2 introduces the mathematical tools of the geometric approach to Gaussian random variables, the weak stochastic realization of conditional independence (Section 2.4).

Section 3 contains the problem statement, the solution procedure and the weak realization of a tuple of multivariable random variables $\left(Y_{1},Y_{2}\right)$ such that another multivariate Gaussian random variable *W* makes $Y_{1}$ and $Y_{2}$ conditionally independent (Section 2.5). $C_{W}\left(Y_{1},Y_{2}\right)=C\left(Y_{1},Y_{2}\right)$.

Section 4 is concerned with the characterization of the Gray–Wyner rate region $R_{GW}\left(\Delta_{1},\Delta_{2}\right)$, the characterization of rates that lie on the Pangloss Plane, and Wyner’s lossy common information. This section includes calculations of the rate distortion functions $R_{Y_{1},Y_{2}}\left(\Delta_{1},\Delta_{2}\right)$, $R_{Y_{i}|W}\left(\Delta_{i}\right),R_{Y_{i}}\left(\Delta_{i}\right),i=1,2$, the weak stochastic realizations of the random variables $\left(Y_{1},Y_{2},{\hat{Y}}_{1},{\hat{Y}}_{2},W\right)$ which achieve these rate distortion functions, for jointly multivariate Gaussian random variables with square-error distortion functions.

Section 5 includes remarks on possible extensions.

Appendix A.3 makes use of a matrix equality and a determinant inequality first obtained by Hua LooKeng in 1952, which are used to carry out the optimization problem of Wyner’s lossy common information $C_{W}\left(Y_{1},Y_{2}\right)=C\left(Y_{1},Y_{2}\right)$.

## 2. Probabilistic Properties of Tuples of Random Variables

The reader finds in this section the basic properties associated with:

(1) the transformation of a tuple of Gaussian multivariate random variables $\left(Y_{1},Y_{2}\right)$ in their canonical variable form, and

(2) The parameterization of all jointly Gaussian distributions $P_{Y_{1},Y_{2},W}\left(y_{1},y_{2},w\right)$ by a zero mean Gaussian random variables $W:\Omega \to R^{k}\equiv W$ such that (a) *W* makes the multivariate random variables $\left(Y_{1},Y_{2}\right)$ conditional independent, and (b) The marginal distribution $P_{Y_{1},Y_{2},W}\left(y_{1},y_{1},\infty \right)=P_{Y_{1},Y_{2}}\left(y_{1},y_{2}\right)$ coincides with the joint distribution of the multivariate random variables $\left(Y_{1},Y_{2}\right)$.

### 2.1. Notation of Elements of Probability Theory

The notation used in this paper is briefly specified. Denote by $Z_{+}=\left\{1,2,\ldots ,\right\}$ the set of the integers and by N={0,1,2,…,} the set of the natural integers. For $n\in Z_{+}$ denote the following finite subsets of the above defined sets by $Z_{n}=\left\{1,2,\ldots ,n\right\}$ and $N_{n}=\left\{0,1,2,\ldots ,n\right\}$.

Denote the real numbers by R and the set of positive and of strictly positive real numbers, respectively, by $R_{+}=\left[0,\infty \right)$ and $R_{++}=\left(0,\infty \right)\subset R$. The vector space of *n*-tuples of real numbers is denoted by $R^{n}$. Denote the Borel σ-algebra on this vector space by $B(R^{n})$, hence, $\left(R^{n},B\left(R^{n}\right)\right)$ is a measurable space.

The expression $R^{n\times m}$ denotes the set of *n* by *m* matrices with elements in the real numbers, for $n,\;m\in Z_{+}$. For the symmetric matrix $Q\in R^{n\times n}$, the inequality Q≥0 denotes that for all vectors $u\in R^{n}$ the inequality $u^{T}Qu\geq 0$ holds. Similar, Q>0 denotes that for all $u\in R^{n}\setminus \left\{0\right\}$, $u^{T}Qu>0$. The notation $Q_{1}\leq Q_{2}$ denotes that $Q_{2}-Q_{1}\geq 0$.

Consider a probability space denoted by (Ω,F,P) consisting of a set Ω, a σ-algebra *F* of subsets of Ω, and a probability measure P:F→[0,1].

A real-valued random variable is a function X:Ω→R such that the following set belongs to the indicated σ-algebra, {ω∈Ω|X(ω)∈(−∞,u]}∈F for all u∈R. A random variable taking values in an arbitrary measurable space (X,B(X)) is defined correspondingly by X:Ω→X and $X^{-1}\left(A\right)=\left\{\omega \in \Omega |X\left(\omega \right)\in A\right\}\in B\left(X\right)$, for all A∈B(X). The measure (or distribution if X is an Euclidean space) induced by the random variable on (X,B(X)) is denoted by $P_{X}$ or P(dx). The σ-algebra generated by a random variable X:Ω→X is defined as the smallest σ-algebra containing the subsets $X^{-1}\left(A\right)\in F$ for all A∈B(X). It is denoted by $F^{X}$. The real-valued random variable *X* is called *G*-measurable for a σ-algebra G⊆F if the subset {ω∈Ω|X(ω)∈(−∞,u]}∈G for all u∈R. Denote the set of positive random variables which are measurable on a sub-σ-algebra G⊆F by,

$$L_{+}\left(G\right)=\left\{X:\Omega \to R_{+}=\left[0,\infty \right)|X\;isG-measurable\right\}.$$

The tuple of sub-σ-algebras $F_{1},\;F_{2}\subseteq F$ is called *independent* if $E\left[X_{1}X_{2}\right]=E\left[X_{1}\right]E\left[X_{2}\right]$ for all $X_{1}\in L_{+}\left(F_{1}\right)$ and all $X_{2}\in L_{+}\left(F_{2}\right)$. The definition can be extended to any finite set of independent sub-σ-algebras.

### 2.2. Geometric Approach of Gaussian Random Variables and Canonical Variable Form

The purpose of this section is to introduce the *geometric approach* of a tuple of finite-dimensional Gaussian random variables using the canonical variable form of the tuple introduced by H. Hotelling, [18]. The use of the geometric approach of two Gaussian random variables with respect to the computation of mutual information is elaborated by Gelfand and Yaglom in [19], making reference to an insight due to Kolmogorov. However, the canonical variable form is not given in [19].

An *$R^{n}$-valued Gaussian random variable* with as parameters the *mean value* $m_{X}\in R^{n}$ and the *variance* $Q_{X}\in R^{n\times n}$, $Q_{X}=Q_{X}^{T}\geq 0$, is a function $X:\Omega \to R^{n}$ which is a random variable and such that the measure of this random variable equals a Gaussian measure described by its characteristic function,

$$E\left[\exp\left(iu^{T}X\right)\right]=\exp\left(iu^{T}m_{X}-\frac{1}{2}u^{T}Q_{X}u\right),\;\forall \;u\in R^{n}.$$

Note that this definition includes the case in which the random variable is almost surely equal to a constant in which case $Q_{X}=0$. A Gaussian random variable with these parameters is denoted by $X\in G(m_{X},Q_{X})$.

The *effective dimension* of the random variable is denoted by $\dim\left(X\right)=\mathrm{rank}\left(Q_{X}\right)$.

Any tuple of random variables $X_{1},\ldots ,X_{k}$ is called *jointly Gaussian* if the vector ${\left(X_{1}^{T},X_{2}^{T},\ldots ,X_{k}^{T}\right)}^{T}$ is a Gaussian random variable. A tuple of Gaussian random variables $\left(Y_{1},Y_{2}\right)$ will be denoted this way to save space, rather than by

$$\begin{array}{c} \left(\begin{array}{c} Y_{1}\\ Y_{2} \end{array}\right). \end{array}$$

Then, the variance matrix of this tuple is denoted by

$$\left(Y_{1},Y_{2}\right)\in G\left(0,Q_{\left(Y_{1},Y_{2}\right)}\right),\;\;Q_{\left(Y_{1},Y_{2}\right)}=\left(\begin{array}{cc} Q_{Y_{1}} & Q_{Y_{1},Y_{2}}\\ Q_{Y_{1},Y_{2}}^{T} & Q_{Y_{2}} \end{array}\right)\in R^{\left(p_{1}+p_{2}\right)\times \left(p_{1}+p_{2}\right)}.$$

The reader should distinguish the variance matrices $Q_{\left(Y_{1},Y_{2}\right)}$ and $Q_{Y_{1},Y_{2}}\in R^{p_{1}\times p_{2}}$. Any such tuple of Gaussian random variables is independent if and only if $Q_{Y_{1},Y_{2}}=0$.

**Definition** **1.** *The canonical variable form.*

*Consider a tuple of Gaussian random variables $Y_{i}:\Omega \to R^{p_{i}}$, with $Q_{Y_{i}}>0$, for i=1,2, $\left(Y_{1},Y_{2}\right)\in G\left(0,Q_{\left(Y_{1},Y_{2}\right)}\right)$, $Q_{\left(Y_{1},Y_{2}\right)}\geq 0$. Define the* canonical variable form *of these random variables if a basis has been chosen and a transformation of the random variables to this basis has been carried out such that with respect to the new basis, one has the representation*

$$\left(Y_{1},Y_{2}\right)\in G\left(0,Q_{\mathrm{cvf}}\right),\;\;where,$$

(50) $$\begin{array}{c} Q_{\mathrm{cvf}}=\left(\begin{array}{cccccc} I_{p_{11}} & 0 & 0 & I_{p_{21}} & 0 & 0\\ 0 & I_{p_{12}} & 0 & 0 & D & 0\\ 0 & 0 & I_{p_{13}} & 0 & 0 & 0\\ I_{p_{21}} & 0 & 0 & I_{p_{21}} & 0 & 0\\ 0 & D & 0 & 0 & I_{p_{22}} & 0\\ 0 & 0 & 0 & 0 & 0 & I_{p_{23}} \end{array}\right)\in R^{p\times p},\\ p,\;p_{1},\;p_{2},\;p_{11},\;p_{12},\;p_{13},\;p_{21},\;p_{22},\;p_{23}\in N,\\ p=p_{1}+p_{2},\;p_{1}=p_{11}+p_{12}+p_{13},\;p_{2}=p_{21}+p_{22}+p_{23},\;p_{11}=p_{21},\;p_{12}=p_{22}, \end{array}$$

(51) $$D=\mathrm{Diag}\left(d_{1},\ldots ,d_{p_{12}}\right),\;\;1>d_{1}\geq d_{2}\geq \ldots \geq d_{p_{12}}>0,$$

(52) $$Y=\left(\begin{array}{c} Y_{1}\\ Y_{2} \end{array}\right)=\left(\begin{array}{c} Y_{11}\\ Y_{12}\\ Y_{13}\\ Y_{21}\\ Y_{22}\\ Y_{23} \end{array}\right),\;\;Y_{ij}:\Omega \to R^{p_{ij}},\;i=1,2,\;j=1,2,3.$$

*One then says that $\left(Y_{11},\ldots ,Y_{1k_{1}}\right)$, $\left(Y_{21},\ldots ,Y_{2k_{2}}\right)$ are the canonical variables and $\left(d_{1},\ldots ,d_{k_{12}}\right)$ the canonical correlation coefficients.*

*If $Q_{\left(Y_{1},Y_{2}\right)}>0$ then necessarily $p_{11}=p_{21}=0$.*

Appendix A.1 gives Algorithm A1 to transform the variance matrix $Q_{\left(Y_{1},Y_{2}\right)}\geq 0$ by two nonsingular transformations $S_{i}\in R^{p_{i}\times p_{i}},i=1,2$, to its canonical variable form $Q_{\mathrm{cvf}}$ of Definition 1, such that

(53) $$S_{1}Y_{1}=\left(V_{1},Y_{1}^{\prime }\right)=\left(\left(Y_{11},Y_{12}\right),Y_{13}\right),\;S_{2}Y_{2}=\left(V_{2},Y_{2}^{\prime }\right)=\left(\left(Y_{21},Y_{22}\right),Y_{23}\right),$$

(54) $$Y_{11}=Y_{21}-a.s.,\;E\left[Y_{12}Y_{22}^{T}\right]=D.$$

**Proposition** **1.** *Properties of components of the canonical variable form.*

*Consider a tuple $\left(Y_{1},Y_{2}\right)\in G\left(0,Q_{\mathrm{cvf}}\right)$ of Gaussian random variables in the canonical variable form.*

*(a) The three components $Y_{11},Y_{12},Y_{13}$ of $Y_{1}$ are independent random variables. Similarly, the three components $Y_{21},Y_{22},Y_{23}$ of $Y_{2}$ are independent random variables.*

*(b) The equality $Y_{11}=Y_{21}$ of these random variables holds almost surely.*

*(c) The tuple of random variables $\left(Y_{12},Y_{22}\right)$ is correlated as shown by the formula*

(55) $$E\left[Y_{12}Y_{22}^{T}\right]=D=\mathrm{Diag}\left(d_{1},\ldots ,d_{p_{12}}\right).$$

*Note that the different components of $Y_{12}$ and of $Y_{22}$ are independent random variables; thus, $Y_{12,i}$ and $Y_{12,j}$ are independent, and $Y_{22,i}$ and $Y_{22,j}$ are independent, and $Y_{12,i}$ and $Y_{22,j}$ are independent, for all i≠j; and that $Y_{12,j}$ and $Y_{22,j}$ for $j=1,\ldots ,p_{12}=p_{22}$ are correlated.*

*(d) The random variable $Y_{13}$ is independent of $Y_{2}$. Similarly, the random variable $Y_{23}$ is independent of $Y_{1}$*

**Proof.**  The results are immediately obvious from the fact that the random variables are all jointly Gaussian and from the variance formula (51) of the canonical variable form. □

Next, the interpretation of the various components of the canonical variable form is defined, as in [20].

**Definition** **2.** *Interpretation of components of the canonical variable form.*

*Consider a tuple of jointly Gaussian random variables $\left(Y_{1},Y_{2}\right)\in G\left(0,Q_{\mathrm{cvf}}\right)$ in the canonical variable form of Definition 1. Call the various components as defined in the next table.*

$Y_{11}=Y_{21}-a.s.$

identical information *of $Y_{1}$ and $Y_{2}$*

$Y_{12}$

correlated information *of $Y_{1}$ with respect to $Y_{2}$*

$Y_{13}$

private information *of $Y_{1}$ with respect to $Y_{2}$ *

$Y_{21}=Y_{11}-a.s.$

identical information *of $Y_{1}$ and $Y_{2}$*

$Y_{22}$

correlated information *of $Y_{2}$ with respect to $Y_{1}$*

$Y_{23}$

private information *of $Y_{2}$ with respect to $Y_{1}$*

*For $Y_{11}=Y_{21}-a.s.$ the term identical information is used*.

Theorem 7 is a formula of mutual information $I(Y_{1};Y_{2})$ for a general tuple of finite-dimensional Gaussian random variables $\left(Y_{1},Y_{2}\right)\in G\left(0,Q_{\left(Y_{1},Y_{2}\right)}\right)$. This formula is the subject of much discussion in Gelfand and Yaglom [19] (see Equantion (2.8’) and Chapter II).

**Theorem** **7.** *Consider a tuple of finite-dimensional Gaussian random variables $\left(Y_{1},Y_{2}\right)\in G\left(0,Q_{\left(Y_{1},Y_{2}\right)}\right)$, $Q_{Y_{i}}>0,i=1,2$.*

*Compute the canonical variable form of the tuple of Gaussian random variables according to Algorithm A1. This yields the indices $p_{11}=p_{21}$, $p_{12}=p_{22}$, $p_{13}$, $p_{23}$, and $n=p_{11}+p_{12}=p_{21}+p_{22}$ and the diagonal matrix D with canonical correlation coefficients or singular values $d_{i}\in \left(0,1\right)$ for i=1,…,n.*

*Then, mutual information $I(Y_{1};Y_{2})$ is computed according to the formula*

(56) $$\begin{array}{cc} I(Y_{1};Y_{2})= & \left\{\begin{array}{ccc} 0,\; & if & 0=p_{11}=p_{12}=p_{21}=p_{22},\;p_{13}>0,\;p_{23}>0,\\ -\frac{1}{2}\sum_{i=1}^{n}\ln\left(1-d_{i}^{2}\right),\; & if & 0=p_{11}=p_{21},\;p_{12}=p_{22}>0,\;p_{13}\geq 0,\;p_{23}\geq 0,\\ \infty ,\; & if & p_{11}=p_{21}>0,\;p_{12}=p_{22}\geq 0,\;p_{13}\geq 0,\;p_{23}\geq 0. \end{array}\right. \end{array}$$

*where $d_{i}$ are the canonical correlation coefficients, i.e.,*

(57) $$d_{i}=d_{i}\left(Y_{12,i}Y_{22,i}\right)=\frac{E\left\{Y_{12,i}Y_{22,i}\right\}}{\sqrt{E{\left\{Y_{12,i}\right\}}^{2}E{\left\{Y_{22,i}\right\}}^{2}}}=E\left\{Y_{12,i}Y_{22,i}\right\},\;\;i=1,\ldots ,n.$$

**Proof.**  The derivation given in Appendix A.3 of [21] (since it is not given in [19]). □

By the last entry of (57), it is appropriate to consider to only $\left(Y_{1},Y_{2}\right)\in G\left(0,Q_{\left(Y_{1},Y_{2}\right)}\right)$ such that $p_{11}=p_{21}=0$, i.e., by removing the identical components prior to the analysis of mutual information problems.

**Remark** **3.** *The material discussed in Section 1.2 makes use of the concepts of this section. The main point to be made is that, in lossy source coding problems, the source distribution is fixed, while the optimal reproduction distribution needs to be found and realized. Then, a pre-encoder can be used by invoking Algorithm A1.*

### 2.3. Conditional Independence of a Triple of Gaussian Random Variables

The concept of conditional independence is basic to the entire paper. The definition is provided below. The characterization of a Gaussian measure on a triple of Gaussian random variables having the conditional independence property is stated.

**Definition** **3.** *Conditional independence.*

*Consider a probability space (Ω,F,P) and three sub-σ-algebras $F_{1},F_{2},G\subseteq F$. Call the sub-σ-algebras $F_{1}$ and $F_{2}$* conditionally independent *given, or conditioned on, the sub-σ-algebra G if the following factorization property holds:*

(58) $$\begin{array}{c} E\left[Y_{1}Y_{2}|G\right]=E\left[Y_{1}|G\right]E\left[Y_{2}|G\right],\;\;\forall \;\;Y_{1}\in L_{+}\left(F_{1}\right),\;\;Y_{2}\in L_{+}\left(F_{2}\right). \end{array}$$

*Denote this property by $(F_{1},F_{2}|G)\in CI$.*

For Gaussian random variables, the definition of minimality of a Gaussian random variable *X* that makes two Gaussian random variables $\left(Y_{1},Y_{2}\right)$ conditionally independent is needed. The definition is introduced below.

**Definition** **4.** *Minimality of conditional independence of Gaussian random variables.*

*Consider three random variables, $Y_{i}:\Omega \to R^{p_{i}}$ for i=1, 2 and $X:\Omega \to R^{n}$.*

*Call the random variables $Y_{1}$ and $Y_{2}$* Gaussian conditionally independent *conditioned on or given $F^{X}$ if:*

*(1) $(F^{Y_{1}},F^{Y_{2}}|F^{X})\in \mathrm{CI}$;*

*(2) $\left(Y_{1},Y_{2},X\right)$ are jointly Gaussian random variables.*

*The notation $(Y_{1},Y_{2}|X)\in \mathrm{CIG}$ is used to denote this property.*

*Call the random variables $\left(Y_{1},Y_{2}|X\right)$* minimally Gaussian conditionally independent *if*

*(1) They are Gaussian conditionally independent;*

*(2) There does not exist another tuple $\left(Y_{1},Y_{2}|X_{1}\right)$ with $X_{1}:\Omega \to R^{n_{1}}$ such that $(Y_{1},Y_{2}|X_{1})\in \mathrm{CIG}$ and $n_{1}<n$.*

*This property is denoted by $\left(Y_{1},Y_{2}|X_{1}\right)\in {\mathrm{CIG}}_{min}$.*

There exists a simple equivalent condition for the conditional independence of tuple of Gaussian random variables by a third Gaussian random variable. This condition is expressed in terms of parameterizing the variance matrix of the tuple as presented in the next proposition.

**Proposition** **2.** *[22] (Proposition 3.4) Equivalent condition for the conditional independence of the tuple of Gaussian random variables.*

*Consider a triple of jointly Gaussian random variables denoted as $\left(Y_{1},Y_{2},X\right)\in G\left(0,Q\right)$ with $Q_{X}>0$. This triple is Gaussian conditionally independent if and only if*

(59) $$Q_{Y_{1},Y_{2}}=Q_{Y_{1},X}Q_{X}^{-1}Q_{X,Y_{2}}.$$

*It is minimally Gaussian conditionally independent if and only if, in addition, $n=\dim\left(X\right)=\mathrm{rank}\left(Q_{Y_{1},Y_{2}}\right)$.*

It will become apparent in Section 4.4 that the Gray and Wyner lossy rate region $R_{GW}\left(\Delta_{1},\Delta_{2}\right)$ is parameterized by a triple of jointly Gaussian random variables $\left(Y_{1},Y_{2},W\right)$, but not necessarily such that *W* makes $Y_{1}$ and $Y_{2}$ conditionally independent. However, subsets of $R_{GW}\left(\Delta_{1},\Delta_{2}\right)$, are characterized by a triple $\left(Y_{1},Y_{2},W\right)$, such that *W* makes $Y_{1}$ and $Y_{2}$ conditionally independent.

### 2.4. Weak Realization of a Gaussian Probability Measure on a Tuple of Random Variables

This section is motivated by Theorem 9, which states that, among all joint distributions $P_{Y_{1},Y_{2},W}$ induced by a tuple of multivariate correlated Gaussian random variables $\left(Y_{1},Y_{2}\right)$, and an arbitrary random variable W:Ω→W, continuous or discrete-valued, Wyner’s common information $C(Y_{1},Y_{2})$, defined by (13), is minimized by a triple $\left(Y_{1},Y_{2},W\right)$ which induces a jointly Gaussian distribution $P_{Y_{1},Y_{2},W}$, and $W:\Omega \to W=R^{n}$ is finite-dimensional Gaussian random variable.

To develop the above results, use is made of the solution of the problem of the weak Gaussian stochastic realization of a tuple of Gaussian random variables. Specifically, to determine a Gaussian probability measure on a triple of Gaussian random variables such that:

(1) The measure restricted to the first two Gaussian random variables is equal to the considered probability measure;
(2) The third Gaussian random variable makes the other two random variables conditionally independent. This problem does not have a unique solution, there is a set of Gaussian probability measures which meets those conditions. Needed is the parameterization of this set of solutions.

Below, the problem is stated in more detail. Its solution is provided in the next section.

**Problem** **1.** *Weak stochastic realization of a tuple of conditionally independent Gaussian random variables.*

Weak stochastic realization problem of a Gaussian random variable. *Consider a Gaussian measure $P_{0}=G\left(0,Q_{0}\right)$ on the space $\left(R^{p_{1}+p_{2}},B\left(R^{p_{1}+p_{2}}\right)\right)$. Determine the integer n∈N and construct all Gaussian measures on the space $(R^{p_{1}+p_{2}+n},\left(B\left(R^{p_{1}+p_{2}+n}\right)\right)$ such that, if $P_{1}=G\left(0,Q_{1}\right)$ is such a measure with $\left(Y_{1},Y_{2},X\right)\in G\left(0,Q_{1}\right)$, then:*

*(1) $G\left(0,Q_{1}\right){|}_{R^{p_{1}+p_{2}}}=G\left(0,Q_{0}\right)$;*

*(2) $\left(Y_{1},Y_{2}|X\right)\in {\mathrm{CIG}}_{min}$.*

*Here, the indicated random variables $\left(Y_{1},Y_{2},X\right)$ are constructed having the measure $G(0,Q_{1})$ with the dimensions $p_{1},p_{2},n\in Z_{+}$, respectively.*

The next definition and proposition are about the weak Gaussian stochastic realization of a tuple of jointly Gaussian multivariate random variables and its weak stochastic realization.

**Definition** **5.** *Minimality of weak stochastic realization of a tuple of conditionally independent Gaussian random variables.*

*Consider a Gaussian measure $P_{0}=G_{0}\left(0,Q_{\left(y_{1},Y_{2}\right)}\right)$ with zero mean values for a tuple $\left(Y_{1},Y_{2}\right)$ of random variables on the product space $(R^{p_{1}}\times R^{p_{2}},B\left(R^{p_{1}}\right)⊗B\left(R^{p_{2}}\right)$ for $p_{1},\;p_{2}\in Z_{+}$ with*

$$\begin{array}{ccc} Q_{\left(Y_{1},Y_{2}\right)} & = & \left(\begin{array}{cc} Q_{Y_{1}} & Q_{Y_{1},Y_{2}}\\ Q_{Y_{1},Y_{2}}^{T} & Q_{Y_{2}} \end{array}\right),\;\;Q_{Y_{1}}>0,\;Q_{Y_{2}}>0. \end{array}$$

*(a) A weak Gaussian stochastic realization of the Gaussian measure $G_{0}\left(0,Q_{\left(y_{1},Y_{2}\right)}\right)$ is defined to be a Gaussian measure $P_{1}=G_{1}$ if there exists an integer $n\in Z_{+}$ such that the Gaussian measure $G_{1}$ is defined on the space $\left(R^{p_{1}}\times R^{p_{1}}\times R^{n},B\left(R^{p_{1}}\right)⊗B\left(R^{p_{2}}\right)⊗B\left(R^{n}\right)\right)$ associated with random variables in the three spaces denoted, respectively, by $Y_{1}$, $Y_{2}$, and X, and such that:*

*(1) $G_{1}{|}_{R^{p_{1}}\times R^{p_{2}}}=G_{0}\left(0,Q_{\left(Y_{1},Y_{2}\right)}\right)$;*

*(2) $Q_{X}>0$;*

*(3) Conditional independence holds: $P_{Y_{1},Y_{2}|X}=P_{Y_{1}|X}P_{Y_{2}|X},$ where these are Gaussian measures, with means which are linear functions of the random variable X and deterministic variance matrices.*

*(b) The weak Gaussian stochastic realization is called* minimal *if the dimension n of the random variable X is the smallest possible over all weak Gaussian stochastic realizations as defined in (a).*

*(c) A Gaussian random variable representation of a weak Gaussian stochastic realization $G_{1}$ is defined as a triple of random variables satisfying the following relations*

(60) $$\begin{array}{cc} \; & \left(Y_{1},Y_{2},X,V_{1},V_{2}\right),\;p_{V_{1}},\;p_{V_{2}}\in Z_{+},\;p_{V_{1}}\geq p_{1},\;p_{V_{2}}\geq p_{2},\\ \; & Y_{1}:\Omega \to R^{p_{1}},\;Y_{2}:\Omega \to R^{p_{2}},\;V_{1}:\Omega \to R^{p_{v_{1}}},\;V_{2}:\Omega \to R^{p_{v_{2}}},\;X:\Omega \to R^{n},\\ \; & (V_{1},V_{2},X)\in G,\;and\;these\;are\;zero\;mean\;independent\;random\;variables\\ \; & Q_{V_{1}}>0,\;Q_{V_{2}}>0,\;Q_{X}>0; \end{array}$$

(61) $$C_{1}\in R^{p_{1}\times n},\;C_{2}\in R^{p_{2}\times n},\;N_{1}\in R^{p_{1}\times p_{V_{1}}},\;N_{2}\in R^{p_{2}\times p_{V_{2}}},$$

(62) $$Y_{1}=C_{1}\;X+N_{1}\;V_{1},\;$$

(63) $$Y_{2}=C_{2}\;X+N_{2}\;V_{2},\;$$

(64) $$Q_{Y_{1}}=C_{1}Q_{X}C_{1}^{T}+N_{1}Q_{V_{1}}N_{1}^{T},$$

(65) $$Q_{Y_{2}}=C_{2}Q_{X}C_{2}^{T}+N_{2}Q_{V_{2}}N_{2}^{T},$$

(66) $$\begin{array}{cc} \; & Q_{Y_{1},Y_{2}}=C_{1}Q_{X}C_{2}^{T},\\ \; & G_{0}\left(0,Q_{\left(Y_{1},Y_{2}\right)}\right)=G_{1}{|}_{R^{p_{1}}\times R^{p_{2}}}. \end{array}$$

*From the assumptions, it then follows that $\left(Y_{1},Y_{2}\right)$ are Gaussian random variables, hence the last equality makes sense.*

*(d) A minimal Gaussian random variable representation of a weak Gaussian stochastic realization is defined as a triple of random variables as in (c) except that, in addition, it is required that,*

(67) $$\mathrm{rank}\left(C_{1}\right)=n=\mathrm{rank}\left(C_{2}\right).$$

*The case $Q_{X}\geq 0$ in (a).(2) is similar.*

The next proposition shows the equivalence of weak Gaussian stochastic realizations of Definition 5. (a), (b) to Definition 5. (c), (d), respectively.

**Proposition** **3.** *Consider the setting of Definition 5 with $\left(Y_{1},Y_{2}\right)\in G\left(0,Q_{\left(Y_{1},Y_{2}\right)}\right)$ with the representation of (62) and (63).*

*(a) A weak Gaussian stochastic realization in terms of a measure $P_{1}=G_{1}$ as defined in Definition 5. (a) is equivalent to a Gaussian random variable representation of Definition 5. (c).*

*(b) The minimal weak Gaussian stochastic realization of Definition 5. (b) is equivalent to a minimal weak Gaussian random variable representation of Definition 5. (d).*

**Proof.**  The derivation given in Appendix A.5 of [21]. □

**Figure 2:**
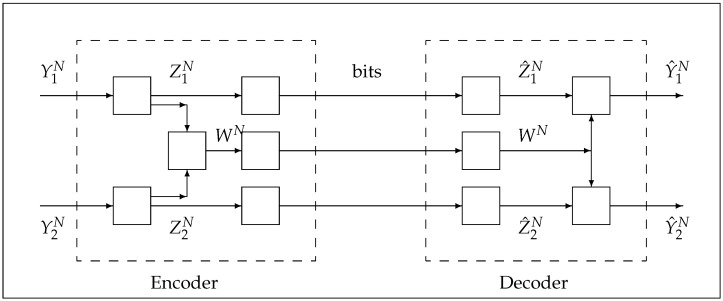
Weak stochastic realization of $\left(Y_{1,i},Y_{2,i}\right)∼P_{Y_{1},Y_{2}},i=1,\ldots ,N$ and $({\hat{Y}}_{1,i},{\hat{Y}}_{2,i}),i=1,\ldots ,N$ at the encoder and decoder with respect to the common and private random variables $\left(W^{N},Z_{1}^{N},Z_{2}^{N}\right),\left(W^{N},{\hat{Z}}_{1}^{N},{\hat{Z}}_{2}^{N}\right)$.

Consider Figure 2. The two signals $Y_{1},Y_{2}$ are to be reproduced at the two decoders by ${\hat{Y}}_{1},{\hat{Y}}_{2}$ subject to the square-error distortion functions. According to Gray and Wyner, the characterization of the lossy rate region is described by a single coding scheme that uses the auxiliary random variable *W*, which is common to both $Y_{1},Y_{2}$. A subset of the rate triples on the Gray and Wyner rate region, which is achieved by a triple that satisfies $(F^{Y_{1}},F^{Y_{2}}|F^{W})\in \mathrm{CIG}$. Below, this conditional independence is further detailed in terms of the mathematical framework of weak stochastic realization such that $(F^{Y_{1}},F^{Y_{2}}|F^{W})\in \mathrm{CIG}$.

**Definition** **6.** *The model for a triple of Gaussian random variables*.

*Consider a tuple of Gaussian random variables specified by $Y=\left(Y_{1},Y_{2}\right)\in G\left(0,Q_{\left(Y_{1},Y_{2}\right)}\right)$ with $Y_{i}:\Omega \to R^{p_{i}}$ for i=1,2. Take a jointly Gaussian measure $G(0,Q_{\left(Y_{1},Y_{2},W\right)})$ for the triple $\left(Y_{1},Y_{2},W\right)$, $W:\Omega \to R^{n}$, $W\in G(0,Q_{W})$, such that the marginal measure on $\left(Y_{1},Y_{2}\right)$ is equal to the considered measure, with*

(68) $$\begin{array}{c} Q_{\left(Y_{1},Y_{2},W\right)}=\left(\begin{array}{ccc} Q_{Y_{1}} & Q_{Y_{1},Y_{2}} & Q_{Y_{1},W}\\ Q_{Y_{1},Y_{2}}^{T} & Q_{Y_{2}} & Q_{Y_{2},W}\\ Q_{Y_{1},W}^{T} & Q_{Y_{2},W}^{T} & Q_{W} \end{array}\right). \end{array}$$

*Denote the parameterized joint measure with respect to W, by $\left(Y_{1},Y_{2},W\right)\in G\left(0,Q_{\left(Y_{1},Y_{2},W\right)}\right)$. This parameterized joint measure $\left(Y_{1},Y_{2},W\right)\in G\left(0,Q_{\left(Y_{1},Y_{2},W\right)}\right)$ also includes the subset such that the conditional independence holds, $(F^{Y_{1}},F^{Y_{2}}|F^{W})\in \mathrm{CIG}$.*

In the following subsections, it will be shown how such a random variable *W* can be constructed in a number of cases.

Algorithm 1. (a) gives the general case, while (b) gives the special case when the joint measure by $\left(Y_{1},Y_{2},W\right)\in G\left(0,Q_{\left(Y_{1},Y_{2},W\right)}\right)$ such that $(F^{Y_{1}},F^{Y_{2}}|F^{W})\in \mathrm{CIG}$ via weak stochastic realization.

**Aldorithm** **1.** *Consider the model of a tuple of Gaussian random variables of Definition 6.*

*(a) General case.*

1. *At the encoder, first compute the variables,*
  (69) $$\left(\begin{array}{c} Z_{1}\\ Z_{2} \end{array}\right)=\left(\begin{array}{c} Y_{1}\\ Y_{2} \end{array}\right)-E\left[\left(\begin{array}{c} Y_{1}\\ Y_{2} \end{array}\right)W^{T}\right]=\left(\begin{array}{c} Y_{1}\\ Y_{2} \end{array}\right)-Q_{(Y_{1},Y_{2}),W}Q_{W}^{†}W$$
  (70) $$Q_{(Y_{1},Y_{2}),W}=E\left[\left(\begin{array}{c} Y_{1}\\ Y_{2} \end{array}\right)W^{T}\right]=\left(\begin{array}{c} Q_{Y_{1},W}\\ Q_{Y_{2},W} \end{array}\right);$$
  *then, the triple*$\left(Z_{1},Z_{2},W\right)$*of jointly Gaussian random variables are such that*$\left(Z_{1},Z_{2}\right)\in G\left(0,Q_{\left(Z_{1},Z_{2}\right)}\right)$*and*$\left(Z_{1},Z_{2}\right)$*independent of W*.
2. *The tuple of random variables* $\left(Y_{1},Y_{2}\right)$ *are represented according to*
(71) $$\left(\begin{array}{c} Y_{1}\\ Y_{2} \end{array}\right)=Q_{(Y_{1},Y_{2}),W}Q_{W}^{†}W+\left(\begin{array}{c} Z_{1}\\ Z_{2} \end{array}\right)$$

*(b) Special case. Consider*$(F^{Y_{1}},F^{Y_{2}}|F^{W})\in \mathrm{CIG}$*, and assume*$Q_{W}>0$.

1. *At the encoder, compute first the variables,*
  (72) $$\begin{array}{cc} Z_{1}= & Y_{1}-E\left[Y_{1}|F^{W}\right]=Y_{1}-Q_{Y_{1},W}Q_{W}^{-1}W, \end{array}$$
  (73) $$\begin{array}{cc} Z_{2}= & Y_{2}-E\left[Y_{2}|F^{W}\right]=Y_{2}-Q_{Y_{2},W}Q_{W}^{-1}W; \end{array}$$
  *then the triple*$\left(Z_{1},Z_{2},W\right)$*of jointly Gaussian random variables are independent.*
2. *The tuple of random variables*$\left(Y_{1},Y_{2}\right)$*are represented according to,*
  (74) $$\begin{array}{c} Y_{1}=Q_{Y_{1},W}Q_{W}^{-1}W+Z_{1},\;\;Y_{2}=Q_{Y_{2},W}Q_{W}^{-1}W+Z_{2}. \end{array}$$

*We emphasize that*$Y_{1}$ and $Y_{2}$ *are conditionally independent condition on W if and only if* $Z_{1}$ and $Z_{2}$ *are independent.*

The validity of the statements of the algorithm follow from the next proposition.

**Proposition** **4.** *Consider the model of a tuple of Gaussian random variables of Definition 6, for cases (a), (b).*

(a) *At the encoder, the conditional expectations are correct and the definitions of $Z_{1}$ and of $Z_{2}$ are well defined.*
(b) *The three random variables $\left(Z_{1},Z_{2},W\right)$ are independent. Consequently, the three sequences*
*$\left(W^{N},Z_{1}^{N},Z_{2}^{N}\right)$, and messages generated by the Gray–Wyner encoder,*
*$f^{\left(E\right)}\left(Y_{1}^{N},Y_{2}^{N}\right)={\bar{f}}^{\left(E\right)}\left(W^{N},Z_{1}^{N},Z_{2}^{N}\right)=\left(S_{0},S_{1},S_{2}\right)$ are independent.*

**Proof.**  Case (a). This follows from realization theory (since no constraints are imposed). Case (b). This is a specific application of Proposition 3. □

For the definition of $C(Y_{1},Y_{2})$, use is made of the construction of the actual family of measures such that $(Y_{1},Y_{2}|W)\in \mathrm{CIG}$ holds, and the weak strochastic realization. These are presented in Theorem 8 and Corollary 1.

### 2.5. Characterization of Minimal Conditional Independence of a Triple of Gaussian Random Variables

Introduce the notation of the parameterization of the family of Gaussian probability distributions

(75) $$\begin{array}{cc} P^{CIG}= & \{P_{Y_{1},Y_{2},W}\left(y_{1},y_{2},w\right)|\;P_{Y_{1},Y_{2}|W}\left(y_{1},y_{2}|w\right)=P_{Y_{1}|W}\left(y_{1}|w\right)P_{Y_{2}|W}\left(y_{2}|w\right),\\ \; & P_{Y_{1},Y_{2},W}\left(y_{1},y_{2},\infty \right)=P_{Y_{1},Y_{2}}\left(y_{1},y_{2}\right),\;\left(Y_{1},Y_{2},W\right)\;is\;jointly\;Gaussian\}. \end{array}$$

A subset of the set $P^{CIG}$ is the set of distributions $P_{min}^{CIG}$, with the additional constraint that the dimension of the random variable *W* is minimal while all other conditions hold, defined by

(76) $$P_{min}^{CIG}=\left\{P_{Y_{1},Y_{2},W}\left(y_{1},y_{2},w\right)\in P^{CIG}|\;\;\left(Y_{1},Y_{2}|W\right)\in {\mathrm{CIG}}_{min}\right\}\subseteq P^{CIG}.$$

The parameterization of the family of Gaussian probability distributions $P^{CIG}$ and $P_{min}^{CIG}$ require the solution of the weak stochastic realization problem of Gaussian random variables defined by Problem 1. This problem is solved in [22] (Theorem 4.2). For the readers’ convenience, it is stated below.

**Theorem** **8.** *Ref. [22] (Theorem 4.2) Consider a tuple $\left(Y_{1},Y_{2}\right)$ of Gaussian random variables in the canonical variable form of Definition 1. Restrict attention to the correlated parts of these random variables. Thus, the random variables $Y_{1},\;Y_{2}$ have the same dimension $n=p_{1}=p_{2}$, and their covariance matrix $D\in R^{n\times n}$ is a nonsingular diagonal matrix with the diagonal ordered real-numbers in the interval (0,1). Hence,*

(77) $$\left(Y_{1},Y_{2}\right)\in G\left(0,Q_{\left(Y_{1},Y_{2}\right)}\right)=P_{0},\;\;Y_{1},Y_{2}:\Omega \to R^{n},\;\;n\in Z_{+},$$

(78) $$Q_{\left(y_{1},y_{2}\right)}=\left(\begin{array}{cc} I & D\\ D & I \end{array}\right),$$

(79) $$D=\mathrm{Diag}\left(d_{1},d_{2},\ldots ,d_{n}\right)\in R^{n\times n},\;\;1>d_{1}\geq d_{2}\geq \ldots \geq d_{n}>0.$$

*That is, $p_{11}=p_{21}=0,p_{13}=p_{23}=0.$*

*(a) There exists a probability measure $P_{1}$, and a triple of Gaussian random variables $Y_{1},Y_{2},$$W:\Omega \to R^{n}$ defined on it, such that (i) $P_{1}{|}_{\left(Y_{1},Y_{2}\right)}=P_{0}$ and (ii) $\left(F^{Y_{1}},F^{Y_{2}}|F^{W}\right)\in {\mathrm{CIG}}_{\min}$;*

*(b) There exist a family of Gaussian measures denoted by $P_{\mathrm{ci}}\subseteq P_{min}^{CIG}$, that satisfy (i) and (ii) of (a), and moreover, this family is parameterized by the matrices and sets, as follows.*

(80) $$G\left(0,Q_{s}\left(Q_{W}\right)\right),\;Q_{W}\in Q_{W},$$

(81) $$\begin{array}{cc} \; & Q_{s}=Q_{s}\left(Q_{W}\right)=\left(\begin{array}{ccc} I & D & D^{1/2}\\ D & I & D^{1/2}Q_{W}\\ D^{1/2} & Q_{W}D^{1/2} & Q_{W} \end{array}\right), \end{array}$$

(82) $$Q_{W}=\left\{Q_{W}\in R^{n\times n}|\;Q_{W}=Q_{W}^{T},\;0<D\leq Q_{W}\leq D^{-1}\right\},$$

(83) $$P_{\mathrm{ci}}=\left\{G\left(0,Q_{s}\left(Q_{W}\right)\right)\;on\;\left(R^{3n},B\left(R^{3n}\right)\right)|\;Q_{W}\in Q_{W}\right\}\subseteq P_{min}^{CIG}.$$

*Furthermore, for any measure $P_{1}\in P_{min}^{CIG}$, there exists a triple of state transformation of the form $\left(Y_{1},Y_{2},W\right)\mapsto \left(S_{1}Y_{1},S_{2}Y_{2},S_{W}W\right)$ for nonsingular square matrices $S_{1},\;S_{2},S_{W}$ such that the corresponding measure of the three transformed variables belongs to $P_{\mathrm{ci}}$.*

The application of Theorem 8 is discussed in the next remark, in the context of parameterizing any rate-triple on the Gray–Wyner lossy rate region $\left(R_{0},R_{1},R_{2}\right)\in R_{GW}\left(\Delta_{1},\Delta_{2}\right)$ that lies on the Pangloss plane.

**Remark** **4.** *Applications of Theorem 8.*

*(a) Theorem 8 is a parameterization of the family of Gaussian measures $P_{\mathrm{ci}}\subseteq P_{min}^{CIG}$ by the entries of the covariance matrix $Q_{W}$. Hence, it is at most an n(n+1)/2− dimensional parameterization;*

*(b) It is shown in Section 4.4 that only a subset of the achievable rate region $R_{GW}\left(\Delta_{1},\Delta_{2}\right)=R_{GW}^{*}\left(\Delta_{1},\Delta_{2}\right)$ is generated from distributions $P_{\mathrm{ci}}\subseteq P_{min}^{CIG}\subseteq P$.*

The next corollary is useful to the calculation of $C(Y_{1},Y_{2})$, since by Theorem 9, an achievable lower bound on $I(Y_{1},Y_{2};W)$ is incurred by a Gaussian random variable *W*, such that the distribution $P_{Y_{1},Y_{2},W}\in P_{\mathrm{ci}}\subseteq P_{min}^{CIG}$, corresponding to $W\in G(0,Q_{W})$. By Theorem 9, and since $C(Y_{1},Y_{2})$ is invariant with respect to nonsingular transformations applied to $\left(Y_{1},Y_{2},W\right)$, the next corollary gives the realization of $\left(Y_{1},Y_{2}\right)$ as defined in Theorem 8, by (77)–(79), expressed in terms of an arbitrary Gaussian random variable $W\in G(0,Q_{W})$.

**Corollary** **1.** *Consider a tuple $\left(Y_{1},Y_{2}\right)$ of Gaussian random variables in the canonical variable form of Definition 1. Restrict the attention to the correlated parts of these random variables, as defined in Theorem 8, by (77)–(79).*

*Then, a realization of the random variables $\left(Y_{1},Y_{2}\right)$ which induce the family of measures $P_{\mathrm{ci}}\subseteq P_{min}^{CIG}$, defined by (80)–(83), is*

(84) $$Y_{1}=Q_{Y_{1},W}Q_{W}^{-1}W+Z_{1}$$

(85) $$Q_{Y_{1},W}=D^{1/2},\;\;Z_{1}\in G\left(0,\left(I-D^{1/2}Q_{W}^{-1}D^{1/2}\right)\right),$$

(86) $$Y_{2}=Q_{Y_{2},W}Q_{W}^{-1}W+Z_{2}$$

(87) $$Q_{Y_{2},W}=D^{1/2}Q_{W},\;\;Z_{2}\in G\left(0,\left(I-D^{1/2}Q_{W}D^{1/2}\right)\right),$$

(88) $$(Z_{1},Z_{2},W),\;are\;independent.$$

*Furthermore, the mutual information $I(Y_{1},Y_{2};W)$ is given by*

(89) $$\begin{array}{cc} I(Y_{1},Y_{2};W)= & H\left(Y_{1},Y_{2}\right)-H\left(Y_{1}|W\right)-H\left(Y_{2}|W\right) \end{array}$$

(90) $$\begin{array}{cc} = & \frac{1}{2}\sum_{i=1}^{n}\ln\left(1-d_{i}^{2}\right)-\frac{1}{2}\ln\left(\det\left(\left[I-D^{1/2}Q_{W}^{-1}D^{1/2}\right]\left[I-D^{1/2}Q_{W}D^{1/2}\right]\right)\right) \end{array}$$

*and it is parameterized by $Q_{W}\in Q_{W}$, where $Q_{W}$ is defined by the set of Equation (82).*

**Proof.**  The correctness of the realization is due to Proposition 2 and Theorem 8. The calculation of mutual information follows from the realization. □

## 3. Wyner’s Common Information

This section is devoted to the calculation of Wyner’s common information $C(Y_{1},Y_{2})$, defined by (13), for $P_{Y_{1},Y_{2}}=G\left(0,Q_{\left(Y_{1},Y_{2}\right)}\right)$, and the construction of the weak stochastic realization of $\left(Y_{1},Y_{2},W\right)$ that achieves this.

### 3.1. Reduction of the Calculation of Wyner’s Common Information

First, we show Theorem 9, which states: given a tuple of multivariate correlated Gaussian random variables $\left(Y_{1},Y_{2}\right)$, and an arbitrary random variable *W* (i.e., taking continuous or discrete values), Wyner’s common information $C(Y_{1},Y_{2})$, defined by (13), is minimized by a triple $\left(Y_{1},Y_{2},W\right)$ which induces a jointly Gaussian distribution $P_{Y_{1},Y_{2},W}$, and $W:\Omega \to W=R^{n}$ is finite-dimensional Gaussian random variable.

**Theorem** **9.** *Consider a tuple of multivariate-correlated Gaussian random variables $Y_{1}:\Omega \to R^{p_{1}},Y_{2}:\Omega \to R^{p_{2}}$, $p_{i}\in Z_{+},i=1,2$ with the variance matrix of this tuple denoted by*

(91) $$\begin{array}{cc} \; & \left(Y_{1},Y_{2}\right)\in G\left(0,Q_{\left(Y_{1},Y_{2}\right)}\right),\;\;Q_{\left(Y_{1},Y_{2}\right)}=\left(\begin{array}{cc} Q_{Y_{1}} & Q_{Y_{1},Y_{2}}\\ Q_{Y_{1},Y_{2}}^{T} & Q_{Y_{2}} \end{array}\right)\in R^{\left(p_{1}+p_{2}\right)\times \left(p_{1}+p_{2}\right)} \end{array}$$

*and, without loss of generality, assume that $Q_{\left(Y_{1},Y_{2}\right)}$ is a positive definite matrix. Let W:Ω→W be any auxiliary random variable, with W being an arbitrary measurable space, and $P_{Y_{1},Y_{2},W}$ any joint probability distribution of the triple $\left(Y_{1},Y_{2},W\right)$ on the product space $(R^{p_{1}}\times R^{p_{2}}\times W,$ $B\left(R^{p_{1}}\right)⊗B\left(R^{p_{2}}\right)⊗B\left(W\right))$ with $(Y_{1},Y_{2})-$ marginal $P_{Y_{1},Y_{2}}$ the Gaussian distribution $P_{Y_{1},Y_{2}}=G\left(0,Q_{\left(Y_{1},Y_{2}\right)}\right)$.*

*The following hold.*

*(a) Define the random variables $Z_{1},Z_{2}$ by*

(92) $$Z_{i}=Y_{i}-E\left[Y_{i}|W\right],\;\;\;\;Z_{i}:\Omega \to R^{p_{i}},\;\;i=1,2.$$

*The inequalities hold:*

(93) $$\begin{array}{cc} I(Y_{1},Y_{2};W)= & H\left(Y_{1},Y_{2}\right)-H\left(Y_{1},Y_{2}|W\right) \end{array}$$

(94) $$\begin{array}{cc} = & H\left(Y_{1},Y_{2}\right)-H\left(Y_{1}|Y_{2},W\right)-H\left(Y_{2}|W\right) \end{array}$$

(95) $$\begin{array}{cc} \geq & H\left(Y_{1},Y_{2}\right)-H\left(Y_{1}|W\right)-H\left(Y_{2}|W\right) \end{array}$$

(96) $$\begin{array}{cc} = & H\left(Y_{1},Y_{2}\right)-H\left(Y_{1}-E\left[Y_{1}|W\right]|W\right)-H\left(Y_{2}-E\left[Y_{2}|W\right]|W\right) \end{array}$$

(97) $$\begin{array}{cc} \geq & H\left(Y_{1},Y_{2}\right)-H\left(Y_{1}-E\left[Y_{1}|W\right]\right)-H\left(Y_{2}-E\left[Y_{2}|W\right]\right) \end{array}$$

(98) $$\begin{array}{cc} = & H\left(Y_{1},Y_{2}\right)-H\left(Z_{1}\right)-H\left(Z_{2}\right) \end{array}$$

(99) $$\begin{array}{cc} \geq & H\left(Y_{1},Y_{2}\right)-H\left(Z_{1}\right)-H\left(Z_{2}\right)\;\;if\;Z_{1},Z_{2}\;have\;finite\;variances,\;and\;Gaussian. \end{array}$$

*(b) If:*

*(i) $W:\Omega \to W=R^{n}$ is an n− dimensional, $n\in Z_{+}$, Gaussian random variable;*

*(ii) $\left(Z_{1},Z_{2},W\right)$ are mutually independent jointly Gaussian random variables, then all inequalities in (93)–(99) hold with equality, and $\left(Y_{1},Y_{2},W\right)$ induces a family of jointly probability distributions $P_{Y_{1},Y_{2},W}$ with $(Y_{1},Y_{2})-$ marginal $P_{Y_{1},Y_{2}}$, such that W makes $Y_{1}$ and $Y_{2}$ conditionally independent, that is $P_{Y_{1},Y_{2}|W}=P_{Y_{1}|W}P_{Y_{2}|W}$;*

*(c) Among all joint distributions, $P_{Y_{1},Y_{2},W}$ induced by the jointly Gaussian random variables $\left(Y_{1},Y_{2}\right)\in G\left(0,Q_{\left(Y_{1},Y_{2}\right)}\right)$ of (91), and an arbitrary random variable W:Ω→W, such that the $(Y_{1},Y_{2})-$ marginal $P_{Y_{1},Y_{2}}$ is the Gaussian distribution $P_{Y_{1},Y_{2}}=G\left(0,Q_{\left(Y_{1},Y_{2}\right)}\right)$, and $P_{Y_{1},Y_{2}|W}=P_{Y_{1}|W}P_{Y_{2}|W}$, a jointly Gaussian distribution achieves the lower bounds of $I(Y_{1},Y_{2};W)$ in part (a), i.e., achieves Wyner’s common information $C(Y_{1},Y_{2})$, defined by (13), and $P_{Y_{1},Y_{2},W}$ is induced by an n− dimensional, $n\in Z_{+}$, Gaussian random variable $W:\Omega \to W=R^{n}$, and $\left(Y_{1},Y_{2}\right)\in G(0,Q_{\left(Y_{1},Y_{2}\right)}$, and such a distribution is induced by the triple $\left(Y_{1},Y_{2},W\right)$ represented by*

(100) $$\begin{array}{cc} \; & Y_{1}=E\left[Y_{1}|W\right]+Z_{1},\;\;Y_{2}=E\left[Y_{2}|W\right]+Z_{2}, \end{array}$$

(101) $$\begin{array}{cc} \; & (W,Z_{1},Z_{2})\;\;\;\;are\;mutually\;independent,\;Gaussian\;random\;variables. \end{array}$$

**Proof.**  (a) (93) is due to an identity of mutual information, (94) is due to the chain rule of entropy, (95) due to conditioning reduces entropy, (96) due to a property of conditional entropy, (97) due to conditioning reduces entropy, (98) is due to definition (92) and (99), is due to maximum entropy principle. (b) Since, $Y_{i}=E\left[Y_{i}|W\right]+Z_{i},i=1,2$ and $\left(Y_{1},Y_{2}\right)\in G\left(0,Q_{\left(Y_{1},Y_{2}\right)}\right)$, if (i) and (ii) hold, then all inequalities hold with equality, and the statements are easily verified. (c) Follows from part (b). □

**Remark** **5.** *Theorem 9 shows that, among all random variables W which induce a joint distribution $P_{Y_{1},Y_{2},W}$, with $(Y_{1},Y_{2})-$ marginal $P_{Y_{1},Y_{2}}$ the Gaussian distribution $P_{Y_{1},Y_{2}}=G\left(0,Q_{\left(Y_{1},Y_{2}\right)}\right)$, then for the Wyner’s common information $C(Y_{1},Y_{2})$ problem, it suffices to consider a jointly Gaussian triple $\left(Y_{1},Y_{2},W\right)$ such that W makes $Y_{1}$ and $Y_{2}$ conditionally independent.*

### 3.2. Wyner’s Common Information of Correlated Random Variables

Assume that the tuple of multivariate correlated Gaussian random variables $\left(Y_{1},Y_{2}\right)\in G\left(0,Q_{\left(Y_{1},Y_{2}\right)}\right)$ of Theorem 9 is already transformed to the canonical variable representation, see Definition 1, using Algorithm A1, i.e., by the nonsingular transformation, $S=\mathrm{Block}-\mathrm{diag}(S_{1},S_{2})$. Mutual information is invariant with respect to nonsingular transformations, and $I\left(Y_{1},Y_{2};W\right)=I\left(S_{1}Y_{1},S_{2}Y_{2};W\right)$.

By Theorem 9. (c), the joint probability distributions $P_{Y_{1},Y_{2},W}\left(y_{1},y_{2},w\right)$ are jointly Gaussian, and parameterized by the random variable *W*. This family of distributions is parameterized by the multidimensional random variable *W*, such that $\left(Y_{1},Y_{2}\right)$ are conditionally independent, conditioned on *W*, the marginal distribution $P_{Y_{1},Y_{2},W}\left(y_{1},y_{2},\infty \right)=P_{Y_{1},Y_{2}}\left(y_{1},y_{2}\right)$ coincides with the distribution of $\left(Y_{1},Y_{2}\right)$, and to represent $\left(Y_{1},Y_{2}\right)$.

Using the above construction, one obtains the next theorem.

**Theorem** **10.** *Consider a tuple of Gaussian random variables $\left(Y_{1},Y_{2}\right)\in G\left(0,Q_{\mathrm{cvf}}\right)$ as described and decomposed according to Algorithm A1. Restrict attention to the correlated parts of these random variables, as described in Theorem 8, (77)–(79) (i.e., only components $\left(Y_{12},Y_{22}\right)$ are present).*

*(a) Theorem 8 holds, and in particular, the family of jointly Gaussian distributions $P_{Y_{1},Y_{2},W}$ induced by $\left(Y_{1},Y_{2}\right)\in G\left(0,Q_{\mathrm{cvf}}\right))$ and a Gaussian random variable $W:\Omega \to R^{n}$, with minimum dimension n, such that the $(Y_{1},Y_{2})-$ marginal is $P_{Y_{1},Y_{2}}=G\left(0,G\left(0,Q_{\mathrm{cvf}}\right)\right)$, and $P_{Y_{1},Y_{2}|W}=P_{Y_{1}|W}P_{Y_{2}|W}$, is paremetrized by the family of Theorem 8. (b), i.e., (80)–(83);*

*(b) Corollary 1, (84)–(88) characterizes the family of realizations of $\left(Y_{1},Y_{2},W\right)$, parameterized by W, which induce jointly Gaussian distributions, such that $W:\Omega \to R^{n}$ is a Gaussian random variable with minimum dimension n, $P_{Y_{1},Y_{2}|W}=P_{Y_{1}|W}P_{Y_{2}|W}$, and $\left(Y_{1},Y_{2}\right)\in G\left(0,Q_{\mathrm{cvf}}\right))$. Moreover, Wyner’s common information $C(Y_{1},Y_{2})$ is computed from the expression $I(Y_{1},Y_{2};W)$ of Corollary 1, (90), optimized over $Q_{W}\in Q_{W}$, where $Q_{W}$ is defined by the set of Equation (82).*

**Remark** **6.** *It is apparent that the proof of the formula $C(Y_{1},Y_{2};W)$ in [15,16] is based on rate distortion function, i.e., they do not directly address Wyner’s optimization problem (13), as in Theorem 9, which first shows, among all continuous or discrete random variables, W is Gaussian, and there is no parameterization of the set of distributions $P_{Y_{1},Y_{2},W}$ achieving conditional independence $P_{Y_{1},Y_{2}|W}=P_{Y_{1}|W}P_{Y_{2}|W}$, i.e., the optimization over the parameterized family of Gaussian measures of Theorem 8 is not given.*

In the next theorem, the family of measures $P_{\mathrm{ci}}\subseteq P_{min}^{CIG}$, defined by (80)–(83), which leads to realization of $\left(Y_{1},Y_{2}\right)$, given in Corollary 1, is ordered for the determination of a single joint distribution $P_{Y_{1},Y_{2},W^{*}}\in P_{\mathrm{ci}}\subseteq P_{min}^{CIG}$, which achieves $C(Y_{1},Y_{2})$. This leads to the realization of $\left(Y_{1},Y_{2}\right)$ expressed in terms of $W^{*}$ and vectors of independent Gaussian random variables $\left(Z_{1},Z_{2}\right)$, one for each realization, each having independent components.

**Theorem** **11.** *Consider a tuple $\left(Y_{1},Y_{2}\right)$ of Gaussian random variables in the canonical variable form of Definition 1. Restrict attention to the correlated parts of these random variables, as defined in Theorem 8, defined by (77)–(79.*

*The following hold.*

*(a) The information quantity $C(Y_{1},Y_{2})$ is given by*

(102) $$\begin{array}{c} C\left(Y_{1},Y_{2}\right)=\frac{1}{2}\sum_{i=1}^{n}\ln\left(\frac{1+d_{i}}{1-d_{i}}\right)=\frac{1}{2}\sum_{i=1}^{n}\ln\left(1+\frac{2d_{i}}{1-d_{i}}\right)\in \left(0,\infty \right). \end{array}$$

*(b) The realizations of the random variables $\left(Y_{1},Y_{2},W^{*}\right)$ that achieve $C(Y_{1},Y_{2})$ are represented by*

$$\begin{array}{cc} \; & V:\Omega \to R^{n},\;V\in G\left(0,I\right),\;\;the\;vector\;V\;has\;independent\;components,\\ \; & F^{V},\;F^{Y_{1}}\vee F^{Y_{2}},\;\;are\;independent\;\sigma -\;algebras, \end{array}$$

(103) $$\begin{array}{cc} \; & L_{1}=L_{2}=D^{1/2}{\left(I+D\right)}^{-1}\in R^{n\times n}, \end{array}$$

(104) $$\begin{array}{cc} \; & L_{3}={\left(I-D\right)}^{1/2}{\left(I+D\right)}^{-1/2}\in R^{n\times n},\;\;L_{1},\;L_{2},\;L_{3},\;are\;diagonal\;matrices, \end{array}$$

(105) $$\begin{array}{cc} \; & W^{*}=L_{1}Y_{1}+L_{2}Y_{2}+L_{3}V,\;\;W^{*}:\Omega \to R^{n}, \end{array}$$

(106) $$\begin{array}{cc} \; & Z_{1}=Y_{1}-D^{1/2}W^{*},\;\;Z_{1}:\Omega \to R^{n}, \end{array}$$

(107) $$\begin{array}{cc} \; & Z_{2}=Y_{2}-D^{1/2}W^{*},\;\;Z_{2}:\Omega \to R^{n}. \end{array}$$

*Then:*

(108) $$Z_{1}\in G\left(0,\left(I-D\right)\right),\;\;Z_{2}\in G\left(0,\left(I-D\right)\right),\;\;W^{*}\in G\left(0,I\right);$$

(109) $$(Z_{1},Z_{2},W^{*}),\;are\;independent\;and$$

(110) $$Y_{1}=D^{1/2}W^{*}+Z_{1},\;\;Y_{2}=D^{1/2}W^{*}+Z_{2}$$

*hence, the variables $\left(Y_{1},Y_{2},W^{*}\right)$ induce a distribution $P_{Y_{1},Y_{2},W^{*}}\in P_{\mathrm{ci}}\subseteq P_{min}^{CIG}$. Note that, in addition, each of the random variables $Z_{1}$, $Z_{2}$, and $W^{*}$ have independent components.*

*(c) The variables $\left(Y_{1},Y_{2},W^{*}\right)$ defined in (b) induce a distribution $P_{Y_{1},Y_{2},W^{*}}\in P_{\mathrm{ci}}\subseteq P_{min}^{CIG}$ which achieves $C(Y_{1},Y_{2})$,*

(111) $$C\left(Y_{1},Y_{2}\right)=I\left(Y_{1},Y_{2};W^{*}\right).$$

**Proof.**  By Theorem 9, the random variables $\left(Y_{1},Y_{2},W\right)$ are restricted to jointly Gaussian random variables. Since mutual information $I(Y_{1},Y_{2};W)$ is invariant with respect to nonsingular transformations $S_{1},S_{2}$, i.e., $I\left(Y_{1},Y_{2};W\right)=I\left(S_{1}Y_{1},SY_{2};W\right)$, and $(F^{Y_{1}},F^{Y_{2}}|F^{W})\in \mathrm{CIG}$ is equivalent to $(F^{S_{1}Y_{1}},F^{S_{2}Y_{2}}|F^{W})\in \mathrm{CIG}$, then it suffices to consider the canonical variable form of Definition 1, and to construct a measure that carries a triple of jointly Gaussian random variables $Y_{1},Y_{2},\;\;W:\Omega \to R^{n}$ such that $(F^{Y_{1}},F^{Y_{2}}|F^{W})\in \mathrm{CIG}$.

(a) (1) Take a probability measure $P_{1}$ such that there exists a triple of Gaussian random variables $Y_{1},Y_{2},\;\;W:\Omega \to R^{n}$ with $P_{1}{|}_{\left(y_{1},y_{2}\right)}=P_{0}$ and $(F^{Y_{1}},F^{Y_{2}}|F^{W})\in \mathrm{CIG}$. It will first be proven that attention can be restricted to those state random variables *W* of which the dimension equals $n=p_{12}=p_{22}$.

Suppose that there exists a state random variable $W:\Omega \to R^{n_{1}}$ such that $\left(F^{Y_{1}},F^{Y_{2}}|F^{W}\right)$∈CIG and $n_{1}>n$. Hence, *W* does not make $\left(Y_{1},Y_{2}\right)$ minimally conditionally independent. Construct a minimal vector which makes the tuple minimally conditionally independent according to the procedure of [22] (Proposition 3.5). Thus,

$$\begin{array}{cc} W_{1}= & E\left[Y_{1}|F^{W}\right]=L_{11}W,\;\;L_{11}\in R^{n\times n_{1}},\\ W_{2}= & E\left[Y_{2}|F^{W_{1}}\right]=L_{12}W_{1},\;\;L_{12}\in R^{n\times n}. \end{array}$$

Then, $\left(F^{Y_{1}},F^{Y_{2}}|F^{W_{2}}\right)\in {\mathrm{CIG}}_{\min}$ and the dimension of $W_{2}$ is $n=p_{12}=p_{22}$. Determine a linear transformation of $W_{2}$ by a matrix $L_{15}\in R^{n\times n}$ such that

$$W_{3}=L_{15}W_{2}=L_{15}L_{12}L_{11}W=L_{13}W,\;\;L_{13}=L_{15}L_{12}L_{11},\;\;W_{3}\in G\left(0,Q_{3}\right),\;\;Q_{3}=I_{n}=L_{13}Q_{W}L_{13}^{T}.$$

It is then possible to construct a matrix $L_{14}\in R^{\left(n_{1}-n\right)\times n_{1}}$ such that

$$W_{4}=L_{14}W,\;\;W_{4}\in G\left(0,Q_{4}\right),\;\;Q_{4}=I,\;\;L_{14}Q_{W}L_{13}^{T}=0;\;\left(\begin{array}{c} W_{3}\\ W_{4} \end{array}\right)\in G\left(0,I_{n_{1}}\right),\;\mathrm{rank}\left(\left(\begin{array}{c} L_{13}\\ L_{14} \end{array}\right)\right)=n_{1},$$

and, due to $L_{14}Q_{W}L_{13}^{T}=0$, $W_{3}$, $W_{4}$ are independent random variables. See [23] [Theorem 4.9] for a theorem with which the existence of $L_{4}$ can be proven. Note further that $F^{W}=F^{W_{3},W_{4}}$.

Hence, the random variables $W_{3},W_{4}$ are independent, $\left(F^{Y_{1}},F^{Y_{2}}|F^{W_{3}}\right)\in {\mathrm{CIG}}_{\min}$, and $I\left(Y_{1},Y_{2};W\right)=I\left(Y_{1},Y_{2};W_{3},W_{4}\right)$.

By properties of mutual information, it now follows that

$$\begin{array}{c} I\left(Y_{1},Y_{2};W_{3},W_{4}\right)-I\left(Y_{1},Y_{2};W_{3}\right)\\ =H\left(Y_{1},Y_{2}\right)+H\left(W_{3},W_{4}\right)-H\left(Y_{1},Y_{2},W_{3},W_{4}\right)-H\left(Y_{1},Y_{2}\right)-H\left(W_{3}\right)+H\left(Y_{1},Y_{2},W_{3}\right)\\ =H\left(Y_{1},Y_{2},W_{3}\right)+H\left(W_{4}\right)-H\left(Y_{1},Y_{2},W_{3},W_{4}\right),\;by\;independence\;ofW_{3}andW_{4};\\ =I(Y_{1},Y_{2},W_{3};W_{4})\geq 0. \end{array}$$

Thus, for the computation of $C(Y_{1},Y_{2})$, attention can be restricted to those state variables *W* which are of miminal dimension.

(2) Take a probability measure $P_{1}$ such that there exists a triple of Gaussian random variables $Y_{1},Y_{2},W:\Omega \to R^{n}$ with $P_{1}{|}_{\left(Y_{1},Y_{2}\right)}=P_{0}$ and $\left(F^{Y_{1}},F^{Y_{2}}|F^{W}\right)\in {\mathrm{CIG}}_{\min}$.

According to [22] (Theorem 4.2), there exist in general many such measures which are parameterized by the matrices and the sets, as stated in Theorem 8, (b), and defined by (80)–(83).

(3) Then, the mutual information of the triple of Gaussian random variables is calculated, using Theorem 8. (b) for any choice of $Q_{W}\in Q_{W}$, where $Q_{W}$ is given by (82). Then

$$I\left(Y_{1},Y_{2};W\right)=H\left(Y_{1},Y_{2}\right)-H\left(Y_{1}|W\right)-H\left(Y_{2}|W\right).$$

The following calculations are then obvious:

$$\begin{array}{cc} \det(Q_{\left(Y_{1},Y_{2}\right)})= & \det\left(\begin{array}{cc} I & D\\ D & I \end{array}\right)=\det\left(I-D^{2}\right)=\prod_{i=1}^{n}\left(1-d_{i}^{2}\right);\\ H(Y_{1},Y_{2})= & \frac{1}{2}\ln\left(\det\left(Q_{\left(y_{1},y_{2}\right)}\right)\right)+\frac{1}{2}\left(2n\right)\ln\left(2\pi e\right)=\frac{1}{2}\sum_{i=1}^{n}\ln\left(1-d_{i}^{2}\right)+n\ln\left(2\pi e\right);\\ \; & \;\;\;\;P_{Y_{1}|W}\left(y_{1}|w\right)\in G\left(E\left[Y_{1}|F^{W}\right],Q_{Y_{1}|W}\right),\\ E[Y_{1}|F^{W}]= & Q_{Y_{1},W}Q_{W}^{-1}W=D^{1/2}Q_{W}^{-1}W;\;\;by(81)\\ Q_{Y_{1}|W}= & I-Q_{Y_{1},W}Q_{W}^{-1}Q_{W}Q_{W}^{-1}Q_{Y_{1},W}^{T}=I-D^{1/2}Q_{W}^{-1}D^{1/2};\;\;by(81)\\ H(Y_{1}|W)= & \frac{1}{2}\ln\left(\det\left(I-D^{1/2}Q_{W}^{-1}D^{1/2}\right)\right)+\frac{1}{2}n\ln\left(2\pi e\right);\\ E[Y_{2}|F^{W}]= & Q_{Y_{2},W}Q_{W}^{-1}W=D^{1/2}Q_{W}Q_{W}^{-1}W=D^{1/2}W;\\ Q_{Y_{2}|W}= & I-Q_{Y_{2},W}Q_{W}^{-1}Q_{W}Q_{W}^{-1}Q_{Y_{2},W}^{T}=I-D^{1/2}Q_{W}D^{1/2};\\ H(Y_{2}|W)= & \frac{1}{2}\ln\left(\det\left(I-D^{1/2}Q_{W}D^{1/2}\right)\right)+\frac{1}{2}n\ln\left(2\pi e\right). \end{array}$$

From the above calculations, it then follows

(112) $$\begin{array}{cc} I(Y_{1},Y_{2};W)= & H\left(Y_{1},Y_{2}\right)-H\left(Y_{1}|W\right)-H\left(Y_{2}|W\right) \end{array}$$

$$\begin{array}{cc} = & \frac{1}{2}\sum_{i=1}^{n}\ln\left(1-d_{i}^{2}\right)+n\ln\left(2\pi e\right) \end{array}$$

$$\begin{array}{cc} \; & -\frac{1}{2}\ln\left(\det\left(I-D^{1/2}Q_{W}^{-1}D^{1/2}\right)\right)-\frac{1}{2}n\ln\left(2\pi e\right) \end{array}$$

(113) $$\begin{array}{cc} \; & -\frac{1}{2}\ln\left(\det\left(I-D^{1/2}Q_{W}D^{1/2}\right)\right)-\frac{1}{2}n\ln\left(2\pi e\right) \end{array}$$

(114) $$\begin{array}{cc} = & \frac{1}{2}\sum_{i=1}^{n}\ln\left(1-d_{i}^{2}\right)-\frac{1}{2}\ln\left(\det\left(\left[I-D^{1/2}Q_{W}^{-1}D^{1/2}\right]\left[I-D^{1/2}Q_{W}D^{1/2}\right]\right)\right). \end{array}$$

The above calculations verify the statements of Corollary 1.

(4) The computation of $C(Y_{1},Y_{2})$ requires the solution of an optimization problem.

(115) $$\begin{array}{cc} C(Y_{1},Y_{2})= & \underset{P_{1}\in P_{\mathrm{ci}}}{\inf}I\left(Y_{1},Y_{2};W\right)\\ = & \underset{Q_{W}\in Q_{W}}{\inf}\left\{\frac{1}{2}\sum_{i=1}^{n}\ln\left(1-d_{i}^{2}\right)-\frac{1}{2}\ln\left(\det\left(\left[I-D^{1/2}Q_{W}^{-1}D^{1/2}\right]\left[I-D^{1/2}Q_{W}D^{1/2}\right]\right)\right)\right\}. \end{array}$$

Since the first term in (115), $\frac{1}{2}\sum_{i=1}^{n}\ln\left(1-d_{i}^{2}\right)$, does not depend on $Q_{W}$ and the natural logarithm is a strictly increasing function, then

(116) $$C\left(Y_{1},Y_{2}\right)\;\;is\;equivalent\;to:\;\;\underset{Q_{W}\in Q_{W}}{\sup}\det\left[\left(I-D^{1/2}Q_{W}^{-1}D^{1/2}\right)\left(I-D^{1/2}Q_{W}D^{1/2}\right)\right].$$

Define:

(117) $$\begin{array}{cc} L_{1}\left(Q_{W}\right)= & \left(I-D^{1/2}Q_{W}^{-1}D^{1/2}\right)\left(I-D^{1/2}Q_{W}D^{1/2}\right), \end{array}$$

(118) $$\begin{array}{cc} f_{1}\left(Q_{W}\right)= & \det(L_{1}\left(Q_{W}\right)). \end{array}$$

Note that the expression $L_{1}\left(Q_{W}\right)\in R^{n\times n}$ is a non-symmetric square matrix in general.

It will be proven that

(119) $$\begin{array}{cc} f_{1}\left(Q_{W}\right)= & \det\left(L_{1}\left(Q_{W}\right)\right)\leq \det\left({\left[I-D\right]}^{2}\right),\;\;\forall \;Q_{W}\in Q_{W}, \end{array}$$

(120) $$\begin{array}{cc} \det(L_{1}\left(Q_{W}\right))= & \det\left({\left[I-D\right]}^{2}\right)\;\;ifandonlyif\;\;Q_{W}=I. \end{array}$$

From these two relations, it follows that $Q_{W}^{*}=I\in R^{n\times n}$ is the unique solution of the supremization problem.

The inequality in (119) follows from Proposition A4. The equality of (120) is proven in two steps. If $Q_{W}=I$, then the equality of (120) holds as follows from direct substitution in (117). The converse is proven by contradiction. Suppose that $Q_{W}\neq I$. Then, it again follows from Proposition A4 that strict inequality holds in (119). Hence, the equality is proven.

(5) Finally, the value of $C(Y_{1},Y_{2})$ is computed for $Q_{W}^{*}=I$.

$$\begin{array}{cc} C(Y_{1},Y_{2})= & \frac{1}{2}\sum_{i=1}^{n}\ln\left(1-d_{1}^{2}\right)-\frac{1}{2}\ln\left(\det\left(I-D^{1/2}{\left(Q_{W}^{*}\right)}^{-1}D^{1/2}\right)\right)-\frac{1}{2}\ln\left(\det\left(I-D^{1/2}\left(Q_{W}^{*}\right)D^{1/2}\right)\right)\\ = & \frac{1}{2}\sum_{i=1}^{n}\ln\left(1-d_{i}^{2}\right)-\frac{1}{2}2\ln\left(\det\left(I-D\right)\right)\\ = & \frac{1}{2}\sum_{i=1}^{n}\ln\left(1-d_{i}^{2}\right)-\frac{1}{2}\sum_{i=1}^{n}\ln\left({\left(1-d_{i}\right)}^{2}\right)=\frac{1}{2}\sum_{i=1}^{n}\ln\left(\frac{1-d_{i}^{2}}{{\left(1-d_{i}\right)}^{2}}\right)\\ = & \frac{1}{2}\sum_{i=1}^{n}\ln\left(\frac{1+d_{i}}{1-d_{i}}\right)=\frac{1}{2}\sum_{i=1}^{n}\ln\left(1+\frac{2d_{i}}{1-d_{i}}\right). \end{array}$$

(b) It follows from part (a) of the theorem that $C(Y_{1},Y_{2})$ is attained as the mutual information $I(Y_{1},Y_{2};W)$ for a random variable *W* with $Q_{W}=Q=I$. Consider now a triple of random variables $\left(Y_{1},Y_{2},W\right)\in G\left(0,Q_{s}\left(I\right)\right)$ as defined in (80)–(83), hence, $Q_{W}=I$. Denote the random variable *W* from now on by $W^{*}$ to indicate that it achieves the infimum of the definition of $C(Y_{1},Y_{2})$. Thus, $Q_{W^{*}}=I$ and

(121) $$\begin{array}{cc} \; & \left(Y_{12},Y_{22},W^{*}\right)\in G\left(0,Q_{s}\left(I\right)\right),\\ \; & Q_{s}\left(I\right)=\left(\begin{array}{ccc} I & D & D^{1/2}\\ D & I & D^{1/2}\\ D^{1/2} & D^{1/2} & I \end{array}\right)>0. \end{array}$$

Let $V:\Omega \to R^{n_{12}}$ be a Gaussian random variable with V∈G(0,I) which is independent of $\left(Y_{1},Y_{2},W\right)$.

Define the new state variable $\bar{W}=L_{1}Y_{1}+L_{2}Y_{2}+L_{3}V$. Then, $\left(Y_{1},Y_{2},V,W^{*}\right)$ are jointly Gaussian and it has to be shown that then $Q_{\bar{W}}=I$, $Q_{Y_{1},\bar{W}}=D^{1/2}$, and $Q_{Y_{2},\bar{W}}=D^{1/2}$. These equalities follow from simple calculations using the expressions of $L_{1}$, $L_{2}$, and $L_{3}$ which calculations are omitted. It then follows from those calculations and the definition of the Gaussian measure $G(0,Q_{s}\left(I\right))$ that, almost surely, $\bar{W}=W^{*}$.

The signals are then represented by

(122) $$Z_{1}=Y_{1}-E\left[Y_{1}|F^{W^{*}}\right]=Y_{1}-Q_{Y_{1},W^{*}}{\left(Q_{W^{*}}\right)}^{-1}W^{*}=Y_{1}-D^{1/2}W^{*},$$

(123) $$Z_{2}=Y_{2}-E\left[Y_{2}|F^{W^{*}}\right]=Y_{2}-Q_{Y_{2},W^{*}}{\left(Q_{W^{*}}\right)}^{-1}W^{*}=Y_{2}-D^{1/2}W^{*}.$$

It is proven that the triple of random variables $\left(Z_{1},Z_{2},W^{*}\right)$ are independent.

$$\begin{array}{cc} E[Z_{1}{\left(W^{*}\right)}^{T}]= & E\left[Y_{1}{\left(W^{*}\right)}^{T}\right]-D^{1/2}E\left[W^{*}{\left(W^{*}\right)}^{T}\right]=D^{1/2}-D^{1/2}=0,\;\;E\left[Z_{2}{\left(W^{*}\right)}^{T}\right]=0,\\ E[Z_{1}Z_{2}^{T}]= & E[\left(Y_{1}-D^{1/2}W^{*}\right){\left(Y_{2}-D^{1/2}W^{*}\right)}^{T}]=0. \end{array}$$

Hence, the original signals are represented as shown by the formulas,

$$\begin{array}{cc} Y_{1}= & Z_{1}+D^{1/2}W^{*},\;\;by\left(121\right),Q_{y_{1},W^{*}}Q_{W^{*}}^{-1}=D^{1/2},and\;by\;definition\;ofZ_{1},\\ Y_{2}= & Z_{2}+D^{1/2}W^{*},\;\;similarly. \end{array}$$

□

### 3.3. Wyner’s Common Information of Arbitrary Gaussian Random Variables

First, the two special cases of (1) a tuple of independent Gaussian random variables and (2) a tuple of identical Gaussian random variables are analyzed. From those results and that of the previous subsection, one can then prove Wyner’s common information for arbitrary Gaussian random variables.

The special case of the canonical variable form with only private parts is presented below.

**Proposition** **5.** *Consider the case of a tuple of Gaussian vectors with only private parts. Hence, the Gaussian measure is*

(124) $$\left(Y_{13},Y_{23}\right)\in G\left(0,Q_{\left(Y_{13},Y_{23}\right)}\right),\;\;Q_{\left(Y_{13},Y_{23}\right)}=\left(\begin{array}{cc} I & 0\\ 0 & I \end{array}\right),\;Y_{13}:\Omega \to R^{p_{13}},\;Y_{23}:\Omega \to R^{p_{23}}.$$

*(a)*  *The minimal σ-algebra $F^{W}$ which makes $Y_{13},Y_{23}$ conditionally independent is the trivial σ-algebra denoted by $F_{0}=\left\{\emptyset ,\Omega \right\}$. Thus, $(F^{Y_{13}},F^{Y_{23}}|F_{0})\in \mathrm{CI}$. The random variable W, in this case, is the constant $W_{3}=0\in R$, hence $F^{W_{3}}=F_{0}$.*
*(b)*  *Then, $W^{*}=W_{3}$ and*
(125) $$C\left(Y_{1},Y_{2}\right)=C\left(Y_{13},Y_{23}\right)=I\left(Y_{13},Y_{23};W_{3}\right)=0.$$
*(c)*  *The weak stochastic realization that achieves $C(Y_{13},Y_{23})=0$ is*
(126) $$Z_{1}=Y_{13},\;\;Z_{2}=Y_{23},\;\;W_{3}=0.$$

The special case of canonical variable form with only identical parts is presented below.

**Proposition** **6.** *Consider the case of a tuple of Gaussian vectors with only the identical part. Hence the Gaussian measure is,*

(127) $$\begin{array}{cc} \; & Y_{11}:\Omega \to R^{p_{11}},\;\;Y_{21}:\Omega \to R^{p_{21}},\;\;p_{11}=p_{21},\\ \; & \left(Y_{11},Y_{21}\right)\in G\left(0,Q_{\left(Y_{11},Y_{21}\right)}\right),\;\;Q_{\left(y_{11},y_{21}\right)}=\left(\begin{array}{cc} I & I\\ I & I \end{array}\right),\;\;Y_{11}=Y_{21}\;a.s. \end{array}$$

*(a)*  *The only minimal σ-algebra which makes $Y_{11}$ and $Y_{21}$ Gaussian conditional-independent is $F^{Y_{11}}=F^{Y_{21}}$. The state variable is thus, $W_{1}=Y_{11}=Y_{21}$ and $F^{W}=F^{Y_{11}}=F^{Y_{21}}$.*
*(b)*  *Then $C\left(Y_{1},Y_{2}\right)=C\left(Y_{11},Y_{21}\right)=+\infty$.*
*(c)*  *The weak stochastic realization is again simple, the variable W equals the identical component and there is no need to use the signals $Z_{1}$ and $Z_{2}$. Thus, the representations are,*
(128) $$Z_{1}=0\in R,\;\;Z_{2}=0\in R,\;\;W=Y_{11}=Y_{21}.$$

Theorem 4 is now proven. Thus, the setting is that of a tuple of arbitrary Gaussian random variables, *not necessarily restricted to the correlated parts of these random variables of Theorem 8, by (77)–(79)*. It is shown that $C(Y_{1},Y_{2})$ is computed by a decomposition and by the use of the formulas previously obtained in Section 3.2.

**Proof** **of Theorem 4.** (a)

(129) $$\begin{array}{cc} \; & C\left(Y_{1},Y_{2}\right)=\underset{(Y_{1},Y_{2},W)\in \mathrm{CIG}}{\inf}I\left(Y_{1},Y_{2};W\right)\\ = & \inf\;\left\{I\left(Y_{11},Y_{21};W_{1}\right)+I\left(Y_{12},Y_{22};W_{2}\right)+I\left(Y_{13},Y_{23};0\right)\right\},\;\;by\;Proposition\;A1,\\ \geq & \underset{(Y_{1},Y_{2},W)\in \mathrm{CIG}}{\inf}I\left(Y_{11},Y_{21};W_{1}\right)+\underset{(Y_{1},Y_{2},W)\in \mathrm{CIG}}{\inf}I\left(Y_{12},Y_{22};W_{2}\right)+\underset{(Y_{1},Y_{2},W)\in \mathrm{CIG}}{\inf}I\left(Y_{13},Y_{23};0\right)\\ = & \underset{(Y_{11},Y_{21},W_{1})\in \mathrm{CIG}}{\inf}I\left(Y_{11},Y_{21};W_{1}\right)+\underset{(Y_{12},Y_{22},W_{2})\in \mathrm{CIG}}{\inf}I\left(Y_{12},Y_{22};W_{2}\right)+I\left(Y_{13},Y_{23};0\right)\\ = & C\left(Y_{11},Y_{21};W_{1}\right)+C\left(Y_{12},Y_{22};W_{2}\right)+C\left(Y_{13},Y_{23};0\right)\\ = & \left\{\begin{array}{ccc} 0, & if & p_{13}>0,\;p_{23}>0,\;p_{11}=p_{12}=p_{21}=p_{22}=0,\\ \frac{1}{2}\sum_{i=1}^{n}\ln\left(\frac{1+d_{i}}{1-d_{i}}\right), & if & p_{12}=p_{22}>0,\;p_{11}=p_{21}=0,\;p_{13}\geq 0,\;p_{23}\geq 0,\\ +\infty , & else. \end{array}\right. \end{array}$$

The latter equality follows from, respectively, Proposition 6, Theorem 11 and Proposition 5

(a and b). It will be shown that $C(Y_{1},Y_{2})$ is less than or equal to the right-hand side of Equation (129). From the latter inequality and the above inequality then follows the expression according to Equation (129).

To be specific, it will be proven that $C(Y_{1},Y_{2})$ is less than the expression $I(Y_{1},Y_{2};W^{*})$ where $W^{*}$ is defined in statement (b) of the proposition. It then follows from the proof of Theorem 11 that $\left(F^{Y_{12}},F^{Y_{22}}|F^{W_{2}^{*}}\right)\in {\mathrm{CIG}}_{\min}$.

Then:

$$\begin{array}{cc} C(Y_{1},Y_{2})= & \underset{(Y_{1},Y_{2}|W)\in \mathrm{CIG}}{\inf}I\left(Y_{1},Y_{2};W\right)\leq I\left(Y_{1},Y_{2};W^{*}\right)\\ = & I\left(Y_{11},Y_{21};W_{1}^{*}\right)+I\left(Y_{12},Y_{22};W_{2}^{*}\right)+I\left(Y_{13},Y_{23};\emptyset \right)\\ = & \left\{\begin{array}{ccc} 0, & if & p_{13}>0,\;p_{23}>0,\;p_{11}=p_{12}=p_{21}=p_{22}=0,\\ \frac{1}{2}\sum_{i=1}^{n}\ln\left(\frac{1+d_{i}}{1-d_{i}}\right), & if & p_{12}=p_{22}>0,\;p_{11}=p_{21}=0,\;p_{13}\geq 0,\;p_{23}\geq 0,\\ +\infty , & else. \end{array}\right. \end{array}$$

The latter equality is proven as follows. In the first case, when $p_{13}>0$, $p_{23}>0$, and $p_{11}=p_{12}=p_{21}=p_{22}=0$, then $Y_{1}=Y_{13}$ and $Y_{2}=Y_{23}$ are independent random variables. It then follows from Proposition 5 that $I\left(Y_{1},Y_{2};0\right)=I\left(Y_{13},Y_{23};0\right)=0$. In the second case, when $p_{12}=p_{22}>0$, $p_{13}\geq 0$, $p_{23}\geq 0$, and $p_{11}=p_{21}=0$, it follows from Proposition A1 and from Theorem 11 that

$$I\left(Y_{1},Y_{2};W^{*}\right)=I\left(Y_{12},Y_{22};W_{2}^{*}\right)+I\left(Y_{13},Y_{23};0\right)=\frac{1}{2}\sum_{i=1}^{n}\ln\left(\frac{1+d_{i}}{1-d_{i}}\right).$$

In the third case, when $p_{11}=p_{21}>0$ and the other $p_{ij}$ indices are arbitrary, then $I(Y_{1},Y_{2};W^{*})=+\infty$. Hence, the inequality $C(Y_{1},Y_{2})\leq right-handside$ is proven and hence equality holds.

(c) This directly follows from Proposition 4. See also Section 3.6 of [21]. □

A procedure for the numerical calculations of Wyner’s information common information is given in Section 3.7 of [21].

## 4. Parametrization of Gray and Wyner Rate Region and Wyner’s Lossy Common Information

This section is devoted to the characterizations of rates that lie in the Gray–Wyner rate region for a tuple of Gaussian random variables with square error distortion functions.

By Gray–Wyner [1] (Theorem 8), reproduced in Theorem 1 to characterize rate triples $\left(R_{0},R_{1},R_{2}\right)\in R_{GW}\left(\Delta_{1},\Delta_{2}\right)$, it is necessary to:

(i) Characterize the rate distortion functions $R_{Y_{i}}\left(\Delta_{i}\right),R_{Y_{i}|W}\left(\Delta_{i}\right),i=1,2$ and $R_{Y_{1},Y_{2}}\left(\Delta_{1},\Delta_{2}\right)$;

(ii) Construct the realizations that induce the test channels of $R_{Y_{i}}\left(\Delta_{i}\right),R_{Y_{i}|W}\left(\Delta_{i}\right),i=1,2$ and $R_{Y_{1},Y_{2}}\left(\Delta_{1},\Delta_{2}\right)$, and understand the structural properties of the realizations.

### 4.1. Characterizations of Joint, Conditional and Marginal RDFs

Theorem 12 is the characterization of the joint RDF $R_{Y_{1},Y_{2}}\left(\Delta_{1},\Delta_{2}\right)$ from [24].

**Theorem** **12.** *Ref. [24] Consider a tuple of Gaussian random variables $Y_{i}:\Omega \to R^{p_{i}}$, with $\left(Y_{1},Y_{2}\right)\in G\left(0,Q_{\left(Y_{1},Y_{2}\right)}\right)$, $Q_{\left(Y_{1},Y_{2}\right)}\geq 0$ (which implies $Q_{Y_{i}}>0$, for i=1,2). Consider the joint RDF $R_{Y_{1},Y_{2}}\left(\Delta_{1},\Delta_{2}\right)$ with square error distortion functions $D_{Y_{1}}\left(y_{1},{\hat{y}}_{1}\right)=\left|\right|y_{1}-{\hat{y}}_{1}{\left|\right|}_{R^{p_{1}}}^{2},$$\;D_{Y_{2}}\left(y_{2},{\hat{y}}_{2}\right)=\left|\right|y_{2}-{\hat{y}}_{2}{\left|\right|}_{R^{p_{2}}}^{2}$. Then, the following hold:*

*(a) The mutual information $I(Y_{1},Y_{2};{\hat{Y}}_{1},{\hat{Y}}_{2})$ satisfies*

(130) $$I\left(Y_{1},Y_{2};{\hat{Y}}_{1},{\hat{Y}}_{2}\right)\geq I\left(Y_{1},Y_{2};E\left\{Y_{1}|F^{{\hat{Y}}_{1},{\hat{Y}}_{2}}\right\},E\left\{Y_{2}|F^{{\hat{Y}}_{1},{\hat{Y}}_{2}}\right\}\right)$$

*and the mean square error satisfies*

(131) $$E\left\{\left|\right|Y_{i}-{\hat{Y}}_{i}{\left|\right|}_{R^{p_{1}}}^{2}\right\}\geq E\left\{\left|\right|Y_{i}-E\left\{Y_{i}|F^{{\hat{Y}}_{1},{\hat{Y}}_{2}}\right\}{\left|\right|}_{R^{p_{i}}}^{2}\right\},\;\;i=1,2.$$

*Moreover, inequalities in (130) and (131) hold with equality if there exists a jointly Gaussian realization of $\left({\hat{Y}}_{1},{\hat{Y}}_{2}\right)$ or a Gaussian test channel distribution $P_{{\hat{Y}}_{1},{\hat{Y}}_{2}|Y_{1},Y_{2}}$ such that the joint distribution $P_{{\hat{Y}}_{1},{\hat{Y}}_{2},Y_{1},Y_{2}}$ is jointly Gaussian, and such that the following identities both hold;*

(132) $$E\left\{Y_{1}|F^{{\hat{Y}}_{1},{\hat{Y}}_{2}}\right\}=E\left\{Y_{1}|F^{{\hat{Y}}_{1}}\right\}={\hat{Y}}_{1},\;\;\;\;E\left\{Y_{2}|F^{{\hat{Y}}_{1},{\hat{Y}}_{2}}\right\}=E\left\{Y_{2}|F^{{\hat{Y}}_{2}}\right\}={\hat{Y}}_{2}.$$

*(b) A realization that achieves the lower bounds of part (a), i.e., satisfies (132), is the Gaussian realization of $\left(Y_{1},Y_{2},{\hat{Y}}_{1},{\hat{Y}}_{2}\right)$ given by*

(133) $$\left(\begin{array}{c} {\hat{Y}}_{1}\\ {\hat{Y}}_{2} \end{array}\right)=H\left(\begin{array}{c} Y_{1}\\ Y_{2} \end{array}\right)+\left(\begin{array}{c} V_{1}\\ V_{2} \end{array}\right)$$

(134) $$\left(V_{1},V_{2}\right)\in G\left(0,Q_{\left(V_{1},V_{2}\right)}\right),\;\;\left(V_{1},V_{2}\right)\;\;independent\;of\;\;\left(Y_{1},Y_{2}\right),$$

(135) $$HQ_{\left(Y_{1},Y_{2}\right)}=Q_{\left(Y_{1},Y_{2}\right)}-Q_{\left(E_{1},E_{2}\right)}\geq 0,$$

(136) $$\begin{array}{cc} \; & Q_{\left(V_{1},V_{2}\right)}=Q_{\left(E_{1},E_{2}\right)}H^{T}=HQ_{\left(Y_{1},Y_{2}\right)}-HQ_{\left(Y_{1},Y_{2}\right)}H^{T}\geq 0,\\ \; & ifQ_{\left(Y_{1},Y_{2}\right)}>0then \end{array}$$

(137) $$\begin{array}{cc} \; & H=I_{p_{1}+p_{2}}-Q_{\left(E_{1},E_{2}\right)}Q_{\left(Y_{1},Y_{2}\right)}^{-1},\;\;Q_{\left(V_{1},V_{2}\right)}=Q_{\left(E_{1},E_{2}\right)}-Q_{\left(E_{1},E_{2}\right)}Q_{\left(Y_{1},Y_{2}\right)}^{-1}Q_{\left(E_{1},E_{2}\right)}\geq 0, \end{array}$$

*where $\left(E_{1},E_{2}\right)$ is the error tuple, that satisfies the structural property,*

(138) $$\begin{array}{c} E_{i}=Y_{i}-E\left\{Y_{i}|F^{{\hat{Y}}_{1},{\hat{Y}}_{2}}\right\}=Y_{i}-E\left\{Y_{i}|F^{{\hat{Y}}_{i}}\right\}=Y_{i}-{\hat{Y}}_{i},\;\;i=1,2 \end{array}$$

*and the variance matrix of this tuple is,*

$$\begin{array}{cc} \; & \left(E_{1},E_{2}\right)\in G\left(0,Q_{\left(E_{1},E_{2}\right)}\right),\;\;Q_{\left(E_{1},E_{2}\right)}=\left(\begin{array}{cc} Q_{E_{1}} & Q_{E_{1},E_{2}}\\ Q_{E_{1},E_{2}}^{T} & Q_{E_{2}} \end{array}\right)\in R^{\left(p_{1}+p_{2}\right)\times \left(p_{1}+p_{2}\right)}. \end{array}$$

*(c) The joint RDF $R_{Y_{1},Y_{2}}\left(\Delta_{1},\Delta_{2}\right)$ is characterized by*

(139) $$\begin{array}{cc} \; & R_{Y_{1},Y_{2}}\left(\Delta_{1},\Delta_{2}\right)=\underset{E\left\{\left|\right|E_{i}{\left|\right|}_{R^{p_{i}}}^{2}\right\}=\mathrm{trace}\left(Q_{E_{i}}\right)\leq \Delta_{i},\;i=1,2}{\inf}\frac{1}{2}\ln{\left(\frac{\det(Q_{\left(Y_{1},Y_{2}\right)})}{\det(Q_{\left(E_{1},E_{2}\right)})}\right)}^{+}\in \left[0,\infty \right], \end{array}$$

(140) $$\begin{array}{cc} \; & such\;that\;\;Q_{\left({\hat{Y}}_{1},{\hat{Y}}_{2}\right)}=Q_{\left(Y_{1},Y_{2}\right)}-Q_{\left(E_{1},E_{2}\right)}\geq 0 \end{array}$$

*where the test channel distribution $P_{{\hat{Y}}_{1},{\hat{Y}}_{2}|Y_{1},Y_{2}}$ or the joint distribution $P_{{\hat{Y}}_{1},{\hat{Y}}_{2},Y_{1},Y_{2}}$ is induced by the realization of part (b).*

The conditional rate distortion function, derived in [25,26], is also required.

**Theorem** **13.** *Ref. [25] (Theorem 1, Thorem 4), [26] Consider a triple of random variables $Y_{i}:\Omega \to R^{p_{i}},i=1,2$, W:Ω→W, where W is continuous or finite-valued, with joint distribution $P_{Y_{1},Y_{2},W}$, and marginal distributions $P_{Y_{1},Y_{2}}$ and $P_{Y_{i}},i=1,2$, the jointly Gaussian distribution $\left(Y_{1},Y_{2}\right)\in G\left(0,Q_{\left(Y_{1},Y_{2}\right)}\right),Q_{\left(Y_{1},Y_{2}\right)}\geq 0$ and $Y_{i}\in G\left(0,Q_{Y_{i}}\right),Q_{Y_{i}}>0,i=1,2$, respectively. Consider the conditional RDFs $R_{Y_{i}|W}\left(\Delta_{i}\right),i=1,2$ with square error distortion functions $D_{Y_{i}}\left(y_{i},{\hat{y}}_{i}\right)=\left|\right|y_{i}-{\hat{y}}_{i}{\left|\right|}_{R^{p_{i}}}^{2},i=1,2$. Then, the following hold.*

*(a) For an arbitrary random variable, W:Ω→W, the mutual information $I(Y_{i};{\hat{Y}}_{i}|W)$ satisfies*

(141) $$\begin{array}{c} I\left(Y_{i};{\hat{Y}}_{i}|W\right)\geq I\left(Y_{i};E\left\{Y_{i}|F^{{\hat{Y}}_{i},W}\right\}|W\right),\;\;i=1,2 \end{array}$$

*and the mean square error satisfies*

(142) $$\begin{array}{c} E\left\{\left|\right|Y_{i}-{\hat{Y}}_{i}{\left|\right|}_{R^{p_{i}}}^{2}\right\}\geq E\left\{\left|\right|Y_{i}-E\left\{Y_{i}|F^{{\hat{Y}}_{i},W}\right\}{\left|\right|}_{R^{p_{i}}}^{2}\right\},\;\;i=1,2. \end{array}$$

*Moreover, inequalities in (141) and (142) hold with equality, if there exists a realization of ${\hat{Y}}_{1}$ of the test channel distribution $P_{{\hat{Y}}_{i}|Y_{i},W}$, such that the joint distribution $P_{{\hat{Y}}_{i},Y_{i},W}$ satisfies the identity:*

(143) $$\begin{array}{c} {\hat{X}}_{i}^{\mathrm{cm}}≐E\left\{Y_{i}|F^{{\hat{Y}}_{i},W}\right\}=E\left\{Y_{i}|F^{{\hat{Y}}_{i}}\right\}={\hat{Y}}_{i},\;\;\;\;i=1,2. \end{array}$$

*(b) Suppose $P_{Y_{1},Y_{2},W}$ is a jointly Gaussian distribution and $W:\Omega \to R^{n}$ is Gaussian. A realization that achieves the lower bounds of part (a), i.e., satisfies (143), is the Gaussian realization of $\left(Y_{i},W,{\hat{Y}}_{i}\right)$ given by*

(144) $$\begin{array}{cc} \; & {\hat{Y}}_{i}=H_{i}Y_{i}+\left(I_{p_{i}}-H_{i}\right)Q_{Y_{i},W}Q_{W}^{†}W+V_{i},\;\;i=1,2, \end{array}$$

(145) $$\begin{array}{cc} \; & V_{i}\in G\left(0,Q_{V_{1}}\right),\;\;V_{i}\;\;independent\;of\;\;Y_{i}, \end{array}$$

(146) $$\begin{array}{cc} \; & H_{i}Q_{Y_{i}|W}=Q_{Y_{i}|W}-Q_{E_{i}}\geq 0, \end{array}$$

(147) $$\begin{array}{cc} \; & Q_{V_{i}}=H_{i}Q_{E_{i}}=H_{i}Q_{Y_{i}|W}-H_{i}Q_{Y_{i}|W}H_{i}^{T}\geq 0, \end{array}$$

*where ${}^{†}$ denotes the pseudoinverse of a matrix, $E_{i}$ is the error, that satisfies the structural property,*

(148) $$\begin{array}{c} E_{i}=Y_{i}-E\left\{Y_{i}|F^{{\hat{Y}}_{i},W}\right\}=Y_{i}-{\hat{Y}}_{i},\;\;E_{i}\in G\left(0,Q_{E_{i}}\right),\;\;i=1,2. \end{array}$$

*The RDF $R_{Y_{i}|W}\left(\Delta_{i}\right)$ for jointly Gaussian $\left(Y_{1},Y_{2},W\right)$ is characterized by*

(149) $$\begin{array}{cc} \; & R_{Y_{i}|W}\left(\Delta_{i}\right)=\underset{E\left\{\left|\right|E_{i}{\left|\right|}_{R^{p_{i}}}^{2}\right\}=\mathrm{trace}\left(Q_{E_{i}}\right)\leq \Delta_{i}}{\inf}\frac{1}{2}\ln{\left(\frac{\det(Q_{Y_{i}|W})}{\det(Q_{E_{i}})}\right)}^{+}\in \left[0,\infty \right],\;\;i=1,2, \end{array}$$

(150) $$\begin{array}{cc} \; & suchthat\;\;Q_{{\hat{Y}}_{i}}=Q_{Y_{i}|W}-Q_{E_{i}}\geq 0, \end{array}$$

*where the test channel distribution $P_{{\hat{Y}}_{i}|Y_{i},W}$ or the joint distribution $P_{{\hat{Y}}_{i},Y_{i},W}$ is induced by the above realization.*

The following is stated as a conjectured, because it is not shown in this paper; it can be shown using Theorem 13.

**Conjecture** **1.** Conditional RDF of Gaussian sources with arbitrary conditioning RV

Consider a triple of random variables $Y_{i}:\Omega \to R^{p_{i}},i=1,2$, W:Ω→W, where *W* is continuous or finite-valued, with joint distribution $P_{Y_{1},Y_{2},W}$, and marginal distributions $P_{Y_{1},Y_{2}}$ and $P_{Y_{i}},i=1,2$, the jointly Gaussian distribution $\left(Y_{1},Y_{2}\right)\in G\left(0,Q_{\left(Y_{1},Y_{2}\right)}\right),Q_{\left(Y_{1},Y_{2}\right)}\geq 0$ and $Y_{i}\in G\left(0,Q_{Y_{i}}\right),Q_{Y_{i}}>0,i=1,2$, respectively. Consider the conditional RDFs $R_{Y_{i}|W}\left(\Delta_{i}\right),i=1,2$ with square error distortion functions $D_{Y_{i}}\left(y_{i},{\hat{y}}_{i}\right)=\left|\right|y_{i}-{\hat{y}}_{i}{\left|\right|}_{R^{p_{i}}}^{2},i=1,2$.

Then, the following hold.

(a) For an arbitrary random variable, W:Ω→W, and ${\hat{X}}_{i}^{\mathrm{cm}}$ satisfying (143), the following lower bounds hold.

(151) $$\begin{array}{cc} I(X_{i};{\hat{X}}_{i}|W)\geq & I(X_{i};{\hat{X}}_{i}^{\mathrm{cm}}|W),\;i=1,2 \end{array}$$

(152) $$\begin{array}{cc} = & \int_{W}I\left(X_{i};{\hat{X}}_{i}^{\mathrm{cm}}|W=w\right)P_{W}\left(dw\right) \end{array}$$

(153) $$\begin{array}{cc} \geq & \underset{w\in W}{\inf}I\left(X_{i};{\hat{X}}_{i}^{\mathrm{cm}}|W=w\right), \end{array}$$

(154) $$\begin{array}{cc} E[D_{X_{i}}\left(X_{i},{\hat{X}}_{i}\right)]= & \int_{W}\left(\int_{R^{p_{1}}\times R^{p_{2}}}D_{X_{i}}\left(x_{i},{\hat{x}}_{i}\right)P_{X_{i},{\hat{X}}_{i}|W}\left(x_{i},{\hat{x}}_{i}|w\right)\right)P_{W}\left(dw\right) \end{array}$$

(155) $$\begin{array}{cc} = & \int_{W}\Delta_{i}\left(w\right)P_{W}\left(dw\right), \end{array}$$

(156) $$\begin{array}{cc} \geq & \int_{W}\Delta_{i}^{\mathrm{cm}}\left(w\right)P_{W}\left(dw\right), \end{array}$$

(157) $$\begin{array}{cc} \geq & \underset{w\in W}{\inf}\Delta_{i}^{\mathrm{cm}}\left(w\right),\;i=1,2, \end{array}$$

where

(158) $$\begin{array}{c} \Delta_{i}\left(w\right)≐E\left[D_{X_{i}}\left(X_{i},{\hat{X}}_{i}\right)|W=w\right],\;\Delta_{i}^{\mathrm{cm}}\left(w\right)≐E\left[D_{X_{i}}\left(X_{i},{\hat{X}}_{i}^{\mathrm{cm}}\right)|W=w\right],\;i=1,2. \end{array}$$

Moreover, the inequalities in (153), (157), are achieved if,

(i) (141) holds, and

(ii) the mutual information $I(X_{i};{\hat{X}}_{i}|W=w)$ and $\Delta_{i}\left(w\right)$ for i=1,2, are independent of w∈W.

(b) The rate distortion function $R_{X_{i}|W}\left(\Delta_{i}\right)$ for W:Ω→W a continuous or finite-valued, achieves a minimum value if,

(i) $W:\Omega \to R^{n}$, $n\in Z_{+}$ is Gaussian, and $P_{Y_{i},W}$ is jointly Gaussian,

(ii) $\left({\hat{X}}_{i},X_{i},W\right)$ is given by the realization of Theorem 13.(b) for i=1,2.

The characterization of the marginal RDFs $R_{Y_{i}}\left(\Delta_{i}\right),i=1,2$—which are well-known, and can be found in many books—is also needed; the weak realization of the test channel, which follows from Theorem 13 (see also [25]), as a degenerate case, and summarized in the next theorem, is important in this paper.

**Theorem** **14.** *Ref. [25] (Theorem 1, Theorem 4) Consider a tuple of Gaussian random variables $Y_{i}:\Omega \to R^{p_{i}},i=1,2$, with $\left(Y_{1},Y_{2}\right)\in G\left(0,Q_{\left(Y_{1},Y_{2}\right)}\right)$, $Q_{\left(Y_{1},Y_{2}\right)}>0$, $Q_{Y_{i}}>0$, for i=1,2.*

*For the marginal RDFs $R_{Y_{i}}\left(\Delta_{i}\right),i=1,2$ with square error distortion functions $D_{Y_{i}}\left(y_{i},{\hat{y}}_{i}\right)=\left|\right|y_{i}-{\hat{y}}_{i}{\left|\right|}_{R^{p_{i}}}^{2}$, the statements of Theorem 13 hold with W, generating the trivial information, i.e., $F^{W}=\left\{\Omega ,\emptyset \right\}$. That is, the marginal RDFs $R_{Y_{i}}\left(\Delta_{i}\right)$ are characterized by*

(159) $$\begin{array}{cc} \; & R_{Y_{i}}\left(\Delta_{i}\right)=\underset{E\left\{\left|\right|E_{i}{\left|\right|}_{R^{p_{i}}}^{2}\right\}=\mathrm{trace}\left(Q_{E_{i}}\right)\leq \Delta_{i}}{\inf}\frac{1}{2}\ln{\left(\frac{\det(Q_{Y_{i}})}{\det(Q_{E_{i}})}\right)}^{+}\in \left[0,\infty \right],\;\;i=1,2, \end{array}$$

(160) $$\begin{array}{cc} \; & suchthat\;\;Q_{{\hat{Y}}_{i}}=Q_{Y_{i}}-Q_{E_{i}}\geq 0 \end{array}$$

*where the test channel distribution $P_{{\hat{Y}}_{i}|Y_{i}}$ or the joint distribution $P_{{\hat{Y}}_{i},Y_{i}}$ is induced by the realization*

(161) $$\begin{array}{cc} \; & {\hat{Y}}_{i}=H_{i}Y_{i}+V_{i},\;\;i=1,2, \end{array}$$

(162) $$\begin{array}{cc} \; & V_{i}\in G\left(0,Q_{V_{1}}\right),\;\;V_{i}\;\;independent\;of\;\;Y_{i}, \end{array}$$

(163) $$\begin{array}{cc} \; & H_{i}Q_{Y_{i}}=Q_{Y_{i}}-Q_{E_{i}}\geq 0, \end{array}$$

(164) $$\begin{array}{cc} \; & Q_{V_{i}}=H_{i}Q_{E_{i}}=H_{i}Q_{Y_{i}}-H_{i}Q_{Y_{i}}H_{i}^{T}\geq 0. \end{array}$$

*and where $E_{i}$ is the error that satisfies the structural property*

(165) $$\begin{array}{c} E_{i}=Y_{i}-E\left\{Y_{i}|F^{{\hat{Y}}_{i}}\right\}=Y_{i}-{\hat{Y}}_{i},\;\;E_{i}\in G\left(0,Q_{E_{i}}\right),\;\;i=1,2. \end{array}$$

Then, we express the characterization of the joint RDF of Theorem 12, using the canonical variable form, and the canonical correlation coefficients. The special case when $Q_{\left(E_{1},E_{2}\right)}$ is is block-diagonal is given in [27].

**Theorem** **15.** *Consider the statement of Theorem 12. Compute the canonical variable form of the tuple of Gaussian random variables $\left(Y_{1},Y_{2}\right)\in G\left(0,Q_{\left(Y_{1},Y_{2}\right)}\right),Q_{Y_{i}}>0$, according to Algorithm A1. This yields the indices $p_{11}=p_{21}$, $p_{12}=p_{22}$, $p_{13}$, $p_{23}$, and $n=p_{11}+p_{12}=p_{21}+p_{22}$, the diagonal matrix $D_{4}$ with canonical correlation coefficients $d_{4,i}\in \left(0,1\right)$ for $i=1,\ldots ,p_{12}$, and decompositions (see Algorithm A1, 1–4)*

(166) $$\begin{array}{c} Q_{Y_{1}}\;=\;U_{1}D_{1}U_{1}^{T},\;\;Q_{Y_{2}}\;=\;U_{2}D_{2}U_{2}^{T}, \end{array}$$

*with $U_{i}\in R^{p_{i}\times p_{i}}$ orthogonal ($U_{i}U_{i}^{T}=I_{p_{i}}=U_{i}^{T}U_{i}$), i=1,2, singular-value decomposition of*

(167) $$\begin{array}{c} D_{1}^{-\frac{1}{2}}U_{1}^{T}Q_{Y_{1}Y_{2}}U_{2}D_{2}^{-\frac{1}{2}}\;=\;U_{3}D_{3}U_{4}^{T}, \end{array}$$

*with $U_{3}\in R^{p_{1}\times p_{1}}$, $U_{4}\in R^{p_{2}\times p_{2}}$ orthogonal,*

(168) $$\begin{array}{cc} D_{3}= & \left(\begin{array}{ccc} I_{p_{11}} & 0 & 0\\ 0 & D_{4} & 0\\ 0 & 0 & 0 \end{array}\right)\in R^{p_{1}\times p_{2}}, \end{array}$$

(169) $$\begin{array}{cc} D_{4}= & \mathrm{Diag}\left(d_{4,1},...,d_{4,p_{12}}\right)\in R^{p_{12}\times p_{12}},\;\;1>d_{4,1}\geq d_{4,2}\geq \ldots \geq d_{4,p_{12}}>0. \end{array}$$

*Define the new variance matrix of $Q_{\left(Y_{1},Y_{2}\right)}$ according to*

(170) $$\begin{array}{c} Q_{\mathrm{cvf}}=\left(\begin{array}{cc} I_{p_{1}} & D_{3}\\ D_{3}^{T} & I_{p_{2}} \end{array}\right). \end{array}$$

*Compute the canonical variable form of the tuple of Gaussian error random variables $\left(E_{1},E_{2}\right)\in G\left(0,Q_{\left(E_{1},E_{2}\right)}\right)$ of Theorem 12.(b), according to Algorithm A1. This yields the indices ${\bar{p}}_{11}={\bar{p}}_{21}$, ${\bar{p}}_{12}={\bar{p}}_{22}$, ${\bar{p}}_{13}$, ${\bar{p}}_{23}$, and $\bar{n}={\bar{p}}_{11}+{\bar{p}}_{12}={\bar{p}}_{21}+{\bar{p}}_{22}$ and the diagonal matrix ${\bar{D}}_{4}$ with canonical correlation coefficients ${\bar{d}}_{4,i}\in \left(0,1\right)$ for $i=1,\ldots ,{\bar{p}}_{12}$, and decompositions (see Algorithm A1, 1–4),*

(171) $$\begin{array}{cc} \; & Q_{E_{1}}\;=\;{\bar{U}}_{1}{\bar{D}}_{1}{\bar{U}}_{1}^{T},\;\;Q_{E_{2}}\;=\;{\bar{U}}_{2}{\bar{D}}_{2}{\bar{U}}_{2}^{T}, \end{array}$$

(172) $$\begin{array}{cc} \; & {\bar{D}}_{i}=\mathrm{Diag}\left({\bar{d}}_{i,1},\ldots ,{\bar{d}}_{i,p_{i}}\right)\in R^{p_{i}\times p_{i}},\;\;{\bar{d}}_{i,1}\geq {\bar{d}}_{i,2}\geq \ldots \geq {\bar{d}}_{i,p_{i}}>0,\;\;i=1,2, \end{array}$$

*with ${\bar{U}}_{i}\in R^{p_{i}\times p_{i}}$ orthogonal (${\bar{U}}_{i}{\bar{U}}_{i}^{T}=I_{p_{i}}={\bar{U}}_{i}^{T}{\bar{U}}_{i}$), i=1,2, singular-value decomposition of*

(173) $$\begin{array}{c} {\bar{D}}_{1}^{-\frac{1}{2}}{\bar{U}}_{1}^{T}Q_{E_{1}E_{2}}{\bar{U}}_{2}{\bar{D}}_{2}^{-\frac{1}{2}}\;=\;{\bar{U}}_{3}{\bar{D}}_{3}{\bar{U}}_{4}^{T}, \end{array}$$

*with ${\bar{U}}_{3}\in R^{p_{1}\times p_{1}}$, ${\bar{U}}_{4}\in R^{p_{2}\times p_{2}}$ orthogonal,*

(174) $$\begin{array}{cc} {\bar{D}}_{3}= & \left(\begin{array}{ccc} I_{{\bar{p}}_{11}} & 0 & 0\\ 0 & {\bar{D}}_{4} & 0\\ 0 & 0 & 0 \end{array}\right)\in R^{p_{1}\times p_{2}}, \end{array}$$

(175) $$\begin{array}{cc} {\bar{D}}_{4}= & \mathrm{Diag}\left({\bar{d}}_{4,1},...,{\bar{d}}_{4,{\bar{p}}_{12}}\right)\in R^{{\bar{p}}_{12}\times {\bar{p}}_{12}},\;\;1>{\bar{d}}_{4,1}\geq {\bar{d}}_{4,2}\geq \ldots \geq {\bar{d}}_{4,{\bar{p}}_{12}}>0. \end{array}$$

*Define the new variance matrix of $Q_{\left(E_{1},E_{2}\right)}$ according to,*

(176) $$\begin{array}{c} {\bar{Q}}_{\mathrm{cvf}}=\left(\begin{array}{cc} I_{p_{1}} & {\bar{D}}_{3}\\ {\bar{D}}_{3}^{T} & I_{p_{2}} \end{array}\right). \end{array}$$

*The joint RDF $R_{Y_{1},Y_{2}}\left(\Delta_{1},\Delta_{2}\right)$ of Theorem 12. (c) is equivalently characterized by*

(177) $$\begin{array}{cc} \; & R_{Y_{1},Y_{2}}\left(\Delta_{1},\Delta_{2}\right)=\underset{Q_{\left(E_{1},E_{2}\right)}\geq 0:\;\bar{n}\in Z_{+},\sum_{i=1}^{p_{1}}{\bar{d}}_{1,i}\leq \Delta_{1},\;\sum_{i=1}^{p_{2}}{\bar{d}}_{2,i}\leq \Delta_{2}}{\inf}\frac{1}{2}\ln{\left(\frac{\det\left(D_{1}\right)\det\left(D_{2}\right)\det\left(Q_{\mathrm{cvf}}\right)}{\det\left({\bar{D}}_{1}\right)\det\left({\bar{D}}_{2}\right)\det\left({\bar{Q}}_{\mathrm{cvf}}\right)}\right)}^{+}\in \left[0,\infty \right], \end{array}$$

(178) $$\begin{array}{cc} \; & such\;that\;\;Q_{\left({\hat{Y}}_{1},{\hat{Y}}_{2}\right)}=Q_{\left(Y_{1},Y_{2}\right)}-Q_{\left(E_{1},E_{2}\right)}\geq 0, \end{array}$$

*where*

(179) $$\begin{array}{cc} \; & \det\left(Q_{\mathrm{cvf}}\right)=\det\left(I_{p_{1}}-D_{3}D_{3}^{T}\right) \end{array}$$

(180) $$\begin{array}{cc} = & \left\{\begin{array}{ccc} 1, & if & p_{13}>0,\;p_{23}>0,\;p_{11}=p_{12}=p_{21}=p_{22}=0,\\ \prod_{i=1}^{n}\left(1-d_{4,i}^{2}\right), & if & p_{11}=p_{21}=0,\;p_{12}=p_{22}=n,\;p_{13}\geq 0,\;p_{23}\geq 0,\\ 0, & if & p_{11}=p_{21}>0,\;p_{12}=p_{22}\geq 0,\;p_{13}\geq 0,\;p_{23}\geq 0, \end{array}\right. \end{array}$$

(181) $$\begin{array}{cc} \; & \det\left({\bar{Q}}_{\mathrm{cvf}}\right)=\det\left(I_{p_{1}}-{\bar{D}}_{3}{\bar{D}}_{3}^{T}\right) \end{array}$$

(182) $$\begin{array}{cc} = & \left\{\begin{array}{ccc} 1, & if & {\bar{p}}_{13}>0,\;{\bar{p}}_{23}>0,\;{\bar{p}}_{11}={\bar{p}}_{12}={\bar{p}}_{21}={\bar{p}}_{22}=0,\\ \prod_{i=1}^{\bar{n}}\left(1-{\bar{d}}_{4,i}^{2}\right), & if & {\bar{p}}_{11}={\bar{p}}_{21}=0,\;{\bar{p}}_{12}={\bar{p}}_{22}=\bar{n},\;{\bar{p}}_{13}\geq 0,\;{\bar{p}}_{23}\geq 0,\\ 0, & if & {\bar{p}}_{11}={\bar{p}}_{21}>0,\;{\bar{p}}_{12}={\bar{p}}_{22}\geq 0,\;{\bar{p}}_{13}\geq 0,\;{\bar{p}}_{23}\geq 0, \end{array}\right. \end{array}$$

*Moreover, a necessary condition for $R_{Y_{1},Y_{2}}\left(\Delta_{1},\Delta_{2}\right)<+\infty$ is ${\bar{p}}_{11}={\bar{p}}_{21}=0$.*

**Proof.**  First, apply Algorithm A1 to the tuple of Gaussian random variables $\left(Y_{1},Y_{2}\right)\in G\left(0,Q_{\left(Y_{1},Y_{2}\right)}\right)$, and then to the Gaussian random variables $\left(E_{1},E_{2}\right)\in G\left(0,Q_{\left(E_{1},E_{2}\right)}\right)$ of Theorem 12. (b). This gives (166)–(176). Then, (177) follows from (139) using (166)–(176), and the standard properties of a determinant of a matrix. The remaining equations are obtained from (166)–(176). The last statement follows from the values of $\det\left({\bar{Q}}_{\mathrm{cvf}}\right),\det\left(Q_{\mathrm{cvf}}\right)$.

□

**Remark** **7.** *By Theorem 15, since $R_{Y_{1},Y_{2}}\left(\Delta_{1},\Delta_{2}\right)\in \left[0,\infty \right]$, by (180), it suffices to consider $Q_{\left(Y_{1},Y_{2}\right)}>0$, which implies $p_{11}=p_{21}=0$, $Q_{Y_{i}}>0,i=1,2$. Furthermore, to ensure $R_{Y_{1},Y_{2}}\left(\Delta_{1},\Delta_{2}\right)\in \left[0,\infty \right)$, it suffices to also consider $Q_{\left(E_{1},E_{2}\right)}>0$, which implies that ${\bar{p}}_{11}={\bar{p}}_{21}=0$, $Q_{E_{i}}>0,i=1,2$.*

From Theorem 15, the next corollary directly follows which identifies the subset of the distortion region such that Gray’s lower bound [28], $R_{Y_{1},Y_{2}}\left(\Delta_{1},\Delta_{2}\right)\geq R_{Y_{2}|Y_{1}}\left(\Delta_{2}\right)+R_{Y_{1}}\left(\Delta_{1}\right)$ holds with equality.

**Corollary** **2.** *Consider the statement of Theorem 15, and without loss of generality, assume $\left(Y_{1},Y_{2}\right)\in G\left(0,Q_{\left(Y_{1},Y_{2}\right)}\right)$, with $Q_{\left(Y_{1},Y_{2}\right)}>0$ (and hence $Q_{Y_{i}}>0,i=1,2$).*

*The joint RDF $R_{Y_{1},Y_{2}}\left(\Delta_{1},\Delta_{2}\right)$ with $D_{1},D_{2},{\bar{D}}_{1},{\bar{D}}_{2},Q_{\mathrm{cvf}},{\bar{Q}}_{\mathrm{cvf}}$, defined in Theorem 15, and corresponding to $p_{11}=p_{21}={\bar{p}}_{11}={\bar{p}}_{21}=0$ satisfies the lower bound ($R_{Y_{2}|Y_{1}}\left(\Delta_{2}\right)$ is obtained from Theorem 13 by letting $W=Y_{1}$.),*

(183) $$\begin{array}{cc} R_{Y_{1},Y_{2}}\left(\Delta_{1},\Delta_{2}\right)\geq & R_{Y_{2}|Y_{1}}\left(\Delta_{2}\right)+R_{Y_{1}}\left(\Delta_{1}\right)\\ = & \underset{E\left\{\left|\right|E_{2}{\left|\right|}_{R^{p_{2}}}^{2}\right\}=\mathrm{trace}\left(Q_{E_{2}}\right)\leq \Delta_{2}}{\inf}\frac{1}{2}\ln\left(\frac{\det(Q_{Y_{2}|Y_{1}})}{\det(Q_{E_{2}})}\right) \end{array}$$

(184) $$\begin{array}{cc} \; & \;\;+\underset{E\left\{\left|\right|E_{1}{\left|\right|}_{R^{p_{1}}}^{2}\right\}=\mathrm{trace}\left(Q_{E_{1}}\right)\leq \Delta_{1}}{\inf}\frac{1}{2}\ln\left(\frac{\det(Q_{Y_{1}})}{\det(Q_{E_{1}})}\right) \end{array}$$

(185) $$\begin{array}{cc} = & \underset{\sum_{i=1}^{p_{2}}{\bar{d}}_{2,i}\leq \Delta_{2}}{\inf}\frac{1}{2}\ln\left(\frac{\det\left(D_{2}\right)\det\left(Q_{\mathrm{cvf}}\right)}{\det({\bar{D}}_{2})}\right)+\underset{\sum_{i=1}^{p_{1}}{\bar{d}}_{1,i}\leq \Delta_{1}}{\inf}\frac{1}{2}\ln\left(\frac{\det(D_{1})}{\det({\bar{D}}_{1})}\right) \end{array}$$

*that is, ${\bar{p}}_{12}={\bar{p}}_{22}=0$.*

*Moreover, the inequalities (184) and (185) hold with the equalities, on the strictly positive surface $D_{C}\left(Y_{1},Y_{2}\right)$, defined by*

(186) $$\begin{array}{c} D_{C}\left(Y_{1},Y_{2}\right)=\left\{\left(\Delta_{1},\Delta_{2}\right)\in \left[0,\infty \right]\times \left[0,\infty \right]|Q_{\left(Y_{1},Y_{2}\right)}-Q_{\left(E_{1},E_{2}\right)}>0\right\}. \end{array}$$

**Proof.**  The lower bound (183) is due to Gray [28]. The equality in (184) follows by using the values of the rate distortion functions in the right hand side of (183). Equality (185) follows from the singular value decomposition of the matrices given in Theorem 15, using $Q_{Y_{2}|Y_{1}}=Q_{Y_{2}}-Q_{Y_{2},Y_{1}}Q_{Y_{1}}^{-1}Q_{Y_{2},Y_{1}}^{T}$. To establish the equalities, note that (177) with $\det({\bar{Q}}_{\mathrm{cvf}})=1$ equivalently ${\bar{p}}_{12}={\bar{p}}_{22}=0$, is precisely (185). Moreover, it can be easily verified that ${\bar{p}}_{12}={\bar{p}}_{22}=0$ for the distortion region $D_{C}\left(Y_{1},Y_{2}\right)$. □

### 4.2. Wyner’s Lossy Common Information of Correlated Gaussian Vectors

Derived in this section are the characterizations of $C_{GW}\left(Y_{1},Y_{2};\Delta_{1},\Delta_{2}\right)$ via Theorem 2, for jointly Gaussian random variables with square-error distortion, as well as $C_{W}\left(Y_{1},Y_{2}\right)$ via Theorem 3.

**Definition** **7.** Wyner’s lossy common information *of a tuple of Gaussian multivariate random variables. Consider a tuple of jointly Gaussian random variables $Y_{1}:\Omega \to R^{p_{1}}\equiv Y_{1}$, $Y_{2}:\Omega \to R^{p_{2}}\equiv Y_{2}$, in terms of the notation $\left(Y_{1},Y_{2}\right)\in G\left(0,Q_{\left(Y_{1},Y_{2}\right)}\right),Q_{Y_{i}}>0,i=1,2$, and square error distortion functions between $\left(y_{1},y_{2}\right)$, and its reproduction $\left({\hat{y}}_{1},{\hat{y}}_{2}\right)$, given by*

(187) $$\begin{array}{c} D_{Y_{1}}\left(y_{1},{\hat{y}}_{1}\right)=\left|\right|y_{1}-{\hat{y}}_{1}{\left|\right|}_{R^{p_{1}}}^{2},\;\;\;\;D_{Y_{2}}\left(y_{2},{\hat{y}}_{2}\right)=\left|\right|y_{2}-{\hat{y}}_{2}{\left|\right|}_{R^{p_{2}}}^{2} \end{array}$$

*where $\left|\right|\cdot {\left|\right|}_{R^{p_{i}}}^{2}$ denotes Euclidean distances on $R^{p_{i}},i=1,2$.*

*(a) Wyner’s common information (information definition) of the tuple of Gaussian random variables $\left(Y_{1},Y_{2}\right)$ is defined by the expression*

(188) $$\begin{array}{c} C\left(Y_{1},Y_{2}\right)=\underset{W:\Omega \to R^{n},\;\left(F^{Y_{1}},F^{Y_{2}}|F^{W}\right)\in \mathrm{CIG}}{\inf}\;I\left(Y_{1},Y_{2};W\right)\in \left[0,\infty \right]. \end{array}$$

*Call any random variable W as defined above such that $\left(Y_{1},Y_{2},W\right)\in G\left(0,Q_{\left(Y_{1},Y_{2},W\right)}\right)$ and $(F^{Y_{1}},F^{Y_{2}}|F^{W})\in \mathrm{CIG}$ a* state *of the tuple $\left(Y_{1},Y_{2}\right)$.*

*If there exists a random variable $W^{*}:\Omega \to R^{n^{*}}$ with $n^{*}\in Z_{+}=\left\{1,2,\ldots ,\right\}$ which attains the infimum; thus, if $C\left(Y_{1},Y_{2}\right)=I\left(Y_{1},Y_{2};W^{*}\right)$, then call that random variable a* minimal information state *of the tuple $\left(Y_{1},Y_{2}\right)$.*

*(b) Wyner’s common information (operational definition) is defined for a tuple of strictly positive real numbers $\gamma =\left(\gamma_{1},\gamma_{2}\right)\in R_{++}\times R_{++}=\left(0,\infty \right)\times \left(0,\infty \right)$ such that, for all $0\leq (\Delta_{1},\Delta_{2})\leq \gamma$,*

(189) $$\begin{array}{cc} C_{GW}(Y_{1},Y_{2}; & \Delta_{1},\Delta_{2})=C_{W}\left(Y_{1},Y_{2}\right)=C\left(Y_{1},Y_{2}\right),\\ \; & for\;\;\left(\Delta_{1},\Delta_{2}\right)\in D_{W}=\left\{\left(\Delta_{1},\Delta_{2}\right)\in \left[0,\infty \right]\times \left[0,\infty \right]|0\leq \left(\Delta_{1},\Delta_{2}\right)\leq \gamma \right\} \end{array}$$

*provided identity (15) holds, i.e., $R_{Y_{1}|W}\left(\Delta_{1}\right)+R_{Y_{2}|W}\left(\Delta_{2}\right)+I\left(Y_{1},Y_{2};W\right)=R_{Y_{1},Y_{2}}\left(\Delta_{1},\Delta_{2}\right)$.*

By the above definition, the problem of calculating Wyner’s lossy common information via (18) is decomposed into the characterization of $C(Y_{1},Y_{2})$ such that identity (15) is satisfied. This follows from the fact that the only difference between $C_{W}\left(Y_{1},Y_{2}\right)$ and $C(Y_{1},Y_{2})$ is the specification of the region $D_{W}$ such that $C_{GW}\left(Y_{1},Y_{2};\Delta_{1},\Delta_{2}\right)=C_{W}\left(Y_{1},Y_{2}\right)=C\left(Y_{1},Y_{2}\right)$ is constant for $\left(\Delta_{1},\Delta_{2}\right)\in D_{W}$.

In the next theorem, we make use of the characterizations of the various rate distortion functions, and the test channel realizations to identify subsets of the rate region that lie on the Pangloss plane, and are consistent with the characterization of Viswanatha, Akyol and Rose [12] (Theorem 1, Equations (19) and (20)).

**Theorem** **16.** *Consider a tuple $\left(Y_{1},Y_{2}\right)$ of Gaussian random variables in the canonical variable form of Definition 1. Restrict the attention to the correlated parts of these random variables, as defined in Theorem 8, by (77)–(79). Furthermore, consider a realization of the random variables $\left(Y_{1},Y_{2}\right)$ which induces the family of measures $P_{\mathrm{ci}}\subseteq P_{min}^{CIG}$, as defined in Corollary 1, by (84)–(88).*

*Then, the following hold.*

*(a) The joint rate distortion function $R_{Y_{1},Y_{2}}\left(\Delta_{1},\Delta_{2}\right)$ of $\left(Y_{1},Y_{2}\right)$ with square error distortion satisfies*

(190) $$\begin{array}{cc} \; & R_{Y_{1},Y_{2}}\left(\Delta_{1},\Delta_{2}\right)=\underset{\sum_{j=1}^{n}\Delta_{1,j}\leq \Delta_{1},\sum_{j=1}^{n}\Delta_{2,j}\leq \Delta_{2}}{\inf}\frac{1}{2}\sum_{j=1}^{n}\ln\left(\frac{\left(1-d_{j}^{2}\right)}{\Delta_{1,j}\Delta_{2,j}}\right),\;\left(\Delta_{1},\Delta_{2}\right)\in D_{C}\left(Y_{1},Y_{2}\right), \end{array}$$

(191) $$\begin{array}{cc} \; & \mathrm{trace}\left(Q_{E_{1}}\right)=E||Y_{1}-{\hat{Y}}_{1}{\left|\right|}_{R^{n}}^{2}=\sum_{j=1}^{n}\Delta_{1,j},\;\;\mathrm{trace}\left(Q_{E_{2}}\right)=E||Y_{2}-{\hat{Y}}_{2}{\left|\right|}_{R^{n}}^{2}=\sum_{j=1}^{n}\Delta_{2,j} \end{array}$$

*where $D_{C}\left(Y_{1},Y_{2}\right)$ is a strictly positive surface, defined by*

(192) $$\begin{array}{c} D_{C}\left(Y_{1},Y_{2}\right)=\left\{\left(\Delta_{1},\Delta_{2}\right)\in \left[0,\infty \right]\times \left[0,\infty \right]|Q_{\left(Y_{1},Y_{2}\right)}-Q_{\left(E_{1},E_{2}\right)}>0\right\} \end{array}$$

*and where $Q_{\left(E_{1},E_{2}\right)}$ is the variance of the errors $E_{i}=Y_{i}-{\hat{Y}}_{i},i=1,2$, with parameters ${\bar{p}}_{11}={\bar{p}}_{21}={\bar{p}}_{12}={\bar{p}}_{22}=0$, and ${\bar{p}}_{13}={\bar{p}}_{23}=n$.*

*The conditional rate distortion functions $R_{Y_{i}|W}\left(\Delta_{i}\right)$ of $Y_{i}$ conditioned on W with square error distortion, and mutual information $I(Y_{1},Y_{2};W)$ satisfy*

(193) $$\begin{array}{cc} \; & R_{Y_{1}|W}\left(\Delta_{1}\right)=\underset{\mathrm{trace}\left(Q_{E_{1}}\right)\leq \Delta_{1}}{\inf}\frac{1}{2}\ln{\left(\frac{\det(I-D^{1/2}Q_{W}^{-1}D^{1/2})}{\det(Q_{E_{1}})}\right)}^{+},\;\;\Delta_{1}\in \left[0,\infty \right) \end{array}$$

(194) $$\begin{array}{cc} \; & R_{Y_{2}|W}\left(\Delta_{2}\right)=\underset{\mathrm{trace}\left(Q_{E_{2}}\right)\leq \Delta_{2}}{\inf}\frac{1}{2}\ln{\left(\frac{\det(I-D^{1/2}Q_{W}D^{1/2})}{\det(Q_{E_{2}})}\right)}^{+},\;\;\Delta_{2}\in \left[0,\infty \right) \end{array}$$

(195) $$\begin{array}{cc} \; & I\left(Y_{1},Y_{2};W\right)=\frac{1}{2}\ln{\left(\frac{\det(I-D^{2})}{\det(\left[I-D^{1/2}D_{W}^{-1}D^{1/2}\right]\left[I-D^{1/2}D_{W}D^{1/2}\right])}\right)}^{+}. \end{array}$$

*where $\mathrm{trace}(Q_{E_{i}}),i=1,2$ are defined as in (191).*

*(b) The representations of reproductions (the reader may verify that the realization satisfies the conditions given in Viswanatha, Akyol and Rose [12], Theorem 1, Equations (19) and (20)), $\left({\hat{Y}}_{1},{\hat{Y}}_{2}\right)$ of $\left(Y_{1},Y_{2}\right)$ at the output of decoder 1 and decoder 2, which achieve the joint rate distortion functions $R_{Y_{1},Y_{2}}\left(\Delta_{1},\Delta_{2}\right),R_{Y_{i}|W}\left(\Delta_{i}\right),i=1,2$ of part (a), are*

(196) $$\begin{array}{cc} \; & Y_{1}=D^{1/2}Q_{W}^{-1}W+Z_{1}, \end{array}$$

(197) $$\begin{array}{cc} \; & Y_{2}=D^{1/2}W+Z_{2}, \end{array}$$

(198) $$\begin{array}{cc} \; & {\hat{Y}}_{1}=Y_{1}-Q_{E_{1}}{\left(I-D^{1/2}Q_{W}^{-1}D^{1/2}\right)}^{-1}Z_{1}+V_{1}, \end{array}$$

(199) $$\begin{array}{cc} \; & \;\;\;\;=D^{1/2}Q_{W}^{-1}W+A_{1}Z_{1}+V_{1}, \end{array}$$

(200) $$\begin{array}{cc} \; & {\hat{Y}}_{2}=Y_{2}-Q_{E_{2}}{\left(I-D^{1/2}Q_{W}D^{1/2}\right)}^{-1}Z_{2}+V_{2}, \end{array}$$

(201) $$\begin{array}{cc} \; & \;\;\;\;=D^{1/2}W+A_{2}Z_{2}+V_{2}, \end{array}$$

(202) $$\begin{array}{cc} \; & Z_{1}\in G\left(0,\left(I-D^{1/2}Q_{W}^{-1}D^{1/2}\right)\right),\;\;Z_{2}\in G\left(0,\left(I-D^{1/2}Q_{W}D^{1/2}\right)\right),\;\;W\in G\left(0,Q_{W}\right), \end{array}$$

(203) $$\begin{array}{cc} \; & Q_{E_{1}}=E\{\left(Y_{1}-{\hat{Y}}_{1}\right){\left(Y_{1}-{\hat{Y}}_{1}\right)}^{T}\},\;\;Q_{E_{2}}=E\{\left(Y_{2}-{\hat{Y}}_{2}\right){\left(Y_{2}-{\hat{Y}}_{2}\right)}^{T}\}, \end{array}$$

(204) $$\begin{array}{cc} \; & V_{1}\in G\left(0,Q_{E_{1}}A_{1}^{T}\right),\;\;V_{2}\in G\left(0,Q_{E_{2}}A_{2}^{T}\right), \end{array}$$

(205) $$\begin{array}{cc} \; & A_{1}=I-Q_{E_{1}}{\left(I-D^{1/2}Q_{W}^{-1}D^{1/2}\right)}^{-1},\;\;A_{2}=I-Q_{E_{2}}{\left(I-D^{1/2}Q_{W}D^{1/2}\right)}^{-1}, \end{array}$$

(206) $$\begin{array}{cc} \; & Q_{E_{i}}=U_{i}\Lambda_{i}U_{i}^{T},\;\;\Lambda_{i}=\mathrm{Diag}\left(\Delta_{i,1},\ldots ,\Delta_{i,n}\right)\in R^{n\times n},\;\;U_{i}U_{i}^{T}=U_{i}^{T}U_{i}=I,\;\;i=1,2, \end{array}$$

(207) $$\begin{array}{cc} \; & Q_{Y_{1}|W}=I-D^{1/2}Q_{W}^{-1}D^{1/2}=U_{1}\Lambda_{Y_{1}|W}U_{1}^{T},\;\;\Lambda_{Y_{1}|W}=\mathrm{Diag}\left(\Lambda_{Y_{1}|W,1},\ldots ,\Lambda_{Y_{1}|W,n}\right), \end{array}$$

(208) $$\begin{array}{cc} \; & Q_{Y_{2}|W}=I-D^{1/2}Q_{W}D^{1/2}=U_{2}\Lambda_{Y_{2}|W}U_{2}^{T},\;\;\Lambda_{Y_{2}|W}=\mathrm{Diag}\left(\Lambda_{Y_{2}|W,1},\ldots ,\Lambda_{Y_{2}|W,n}\right), \end{array}$$

(209) $$\begin{array}{cc} \; & (V_{1},V_{2},Z_{1},Z_{2},W),\;\;\;are\;independent \end{array}$$

*and are parameterized by $Q_{W}\in Q_{W}$, where $Q_{W}$ is defined by the set of Equation (82).*

*Moreover, the joint distribution $P_{Y_{1},Y_{2},{\hat{Y}}_{1},{\hat{Y}}_{2},W}$ satisfies (the reader may verify that conditions (210) are identical to Viswanatha, Akyol and Rose [12], Theorem 1, Equations (19) and (20)) for rates that lie on the Pangloss plane)*

(210) $$\begin{array}{cc} \; & P_{{\hat{Y}}_{1},{\hat{Y}}_{2}|W}=P_{{\hat{Y}}_{1}|W}P_{{\hat{Y}}_{2}|W},\;\;P_{Y_{1},Y_{2}|{\hat{Y}}_{1},{\hat{Y}}_{2},W}=P_{Y_{1},Y_{2}|{\hat{Y}}_{1},{\hat{Y}}_{2}}, \end{array}$$

(211) $$\begin{array}{cc} \; & P_{Y_{1},Y_{2},{\hat{Y}}_{1},{\hat{Y}}_{2},W}=P_{{\hat{Y}}_{1}|Y_{1},W}P_{{\hat{Y}}_{2}|Y_{2},W}P_{Y_{1}|W}P_{Y_{2}|W}P_{W}, \end{array}$$

(212) $$\begin{array}{cc} \; & P_{Y_{1},Y_{2},{\hat{Y}}_{1},{\hat{Y}}_{2},W}=P_{Y_{1}|{\hat{Y}}_{1}}P_{Y_{2}|{\hat{Y}}_{2}}P_{{\hat{Y}}_{1}|W}P_{{\hat{Y}}_{2}|W}P_{W}. \end{array}$$

*(c) Consider part (a) and the realization of part (b). Then, $R_{Y_{1}|W}\left(\Delta_{1}\right)+R_{Y_{2}|W}\left(\Delta_{2}\right)+I\left(Y_{1},Y_{2};W\right)$$=R_{Y_{1},Y_{2}}\left(\Delta_{1},\Delta_{2}\right)$ on the subset $D_{C}\left(Y_{1},Y_{2}\right)$, defined by (192) such that (210) holds.*

*(d) Suppose $Q_{W}=Q_{W^{*}}\in Q_{W}$ is diagonal, i.e., $Q_{W^{*}}=\mathrm{Diag}\left(Q_{W_{1}^{*}},\ldots ,Q_{W_{n}^{*}}\right),\;d_{i}\leq Q_{W_{i}^{*}}\leq d_{i}^{-1},\forall i$. Then, the conditional RDFs $R_{Y_{i}|W^{*}}\left(\Delta_{i}\right)$ are given by*

(213) $$\begin{array}{cc} R_{Y_{1}|W^{*}}\left(\Delta_{1}\right)= & \underset{\sum_{j=1}^{n}\Delta_{1,j}=\Delta_{1}}{\inf}\frac{1}{2}\sum_{j=1}^{n}\ln\left(\frac{\left(1-d_{j}/Q_{W_{j}^{*}}\right)}{\Delta_{1,j}}\right), \end{array}$$

(214) $$\begin{array}{cc} R_{Y_{2}|W^{*}}\left(\Delta_{2}\right)= & \underset{\sum_{j=1}^{n}\Delta_{2,j}=\Delta_{2}}{\inf}\frac{1}{2}\sum_{j=1}^{n}\ln\left(\frac{\left(1-d_{j}Q_{W_{j}^{*}}\right)}{\Delta_{2,j}}\right), \end{array}$$

*and the optimal $\Delta_{1,j},\Delta_{2,j}$ are obtained from the water-filling equations,*

(215) $$\begin{array}{cc} \; & \Delta_{1,j}=\left\{\begin{array}{cc} \lambda , & \lambda <1-d_{j}/Q_{W_{j}^{*}}\;\\ 1-d_{j}, & \lambda \geq 1-d_{j}/Q_{W_{j}^{*}}, \end{array}\right.\;\Delta_{1}\in \left[0,\infty \right), \end{array}$$

(216) $$\begin{array}{cc} \; & \Delta_{2,j}=\left\{\begin{array}{cc} \lambda , & \lambda <1-d_{j}Q_{W_{j}^{*}}\;\\ 1-d_{j}, & \lambda \geq 1-d_{j}Q_{W_{j}^{*}}, \end{array}\right.\;\Delta_{2}\in \left[0,\infty \right). \end{array}$$

*and the representations of part (b) hold, with $Q_{E_{i}},Q_{Y_{i}|W^{*}}$ diagonal matrices.*

**Proof.**  (a) Since the attention is restricted to the correlated parts of these random variables, as defined in Theorem 8, by (77)–(79), then the statements of joint RDF $R_{Y_{1},Y_{2}}\left(\Delta_{1},\Delta_{2}\right)$ of part (a) are a special case of Theorem 12. (c), and obtained from Corollary 2. Similarly, expressions (193)–(195) follow from (13). However, as demonstrated shortly, these also follow, from the derivation of part (b). (b) Recall that the joint rate distortion function is achieved by a jointly Gaussian distribution $P_{Y_{1},Y_{2},{\hat{Y}}_{1},{\hat{Y}}_{2}}$ such that the average square-error distortions are satisfied. Consider the realization of the random variables $\left(Y_{1},Y_{2}\right)$ which induce the family of measures $P_{\mathrm{ci}}\subseteq P_{min}^{CIG}$, as defined in Corollary 1, by (84)–(88). By properties of mutual information, then

(217) $$\begin{array}{cc} I(Y_{1},Y_{2};{\hat{Y}}_{1},{\hat{Y}}_{2})= & H\left(Y_{1},Y_{2}\right)-I\left(Y_{1},Y_{2}|{\hat{Y}}_{1},{\hat{Y}}_{2}\right) \end{array}$$

(218) $$\begin{array}{cc} = & H\left(Y_{1},Y_{2}\right)-H\left(Y_{1}|{\hat{Y}}_{1},{\hat{Y}}_{2},Y_{2}\right)-H\left(Y_{2}|{\hat{Y}}_{1},{\hat{Y}}_{2}\right) \end{array}$$

(219) $$\begin{array}{cc} = & H\left(Y_{1},Y_{2}\right)-H\left(Y_{2}|{\hat{Y}}_{1},{\hat{Y}}_{2},Y_{1}\right)-H\left(Y_{1}|{\hat{Y}}_{1},{\hat{Y}}_{2}\right) \end{array}$$

(220) $$\begin{array}{cc} \geq & H\left(Y_{1},Y_{2}\right)-H\left(Y_{1}|{\hat{Y}}_{1}\right)-H\left(Y_{2}|{\hat{Y}}_{2}\right),\;\;cond.\;reduces\;entropy, \end{array}$$

(221) $$\begin{array}{cc} = & \frac{1}{2}\sum_{i=1}^{n}\ln\left(1-d_{i}^{2}\right)+n\ln\left(2\pi e\right)-H\left(Y_{1}|{\hat{Y}}_{1}\right)-H\left(Y_{2}|{\hat{Y}}_{2}\right)\\ \geq & \frac{1}{2}\sum_{i=1}^{n}\ln\left(1-d_{i}^{2}\right)+n\ln\left(2\pi e\right)-\frac{1}{2}\sum_{i=1}^{n}\ln\left(\Delta_{1,i}\right)-\frac{1}{2}n\ln\left(2\pi e\right) \end{array}$$

(222) $$\begin{array}{cc} \; & -\frac{1}{2}\sum_{i=1}^{n}\ln\left(\Delta_{2,i}\right)-\frac{1}{2}n\ln\left(2\pi e\right),\;\;maximum\;entropy\;of\;Gaus.\;dist. \end{array}$$

(223) $$\begin{array}{cc} = & \frac{1}{2}\sum_{i=1}^{n}\ln\left(\frac{\left(1-d_{i}^{2}\right)}{\Delta_{1,i}\Delta_{2,i}}\right) \end{array}$$

where $\sum_{i=1}^{n}\Delta_{1,i}=E[\;||Y_{1}-{\hat{Y}}_{1}{\left|\right|}_{R^{n}}^{2}]\leq \Delta_{1}$ and $\sum_{i=1}^{n}\Delta_{2,i}=E[\;||Y_{2}-{\hat{Y}}_{2}{\left|\right|}_{R^{n}}^{2}]\leq \Delta_{2}$. The average distortion satisfies

(224) $$\Delta_{1}\geq E[\;||Y_{1}-{\hat{Y}}_{1}{\left|\right|}_{R^{n}}^{2}]\geq E[\;||Y_{1}-E\left[Y_{1}|F^{{\hat{Y}}_{1}}\right]{\left|\right|}_{R^{n}}^{2}],$$

(225) $$\Delta_{2}\geq E[\;||Y_{1}-{\hat{Y}}_{1}{\left|\right|}_{R^{n}}^{2}]\geq E[\;||Y_{1}-E\left[Y_{1}|F^{{\hat{Y}}_{1}}\right]{\left|\right|}_{R^{n}}^{2}].$$

Furthermore,

(226) $$\begin{array}{cc} \; & if\;\;P_{Y_{1},Y_{2}|{\hat{Y}}_{1},{\hat{Y}}_{2}}=P_{Y_{1}|{\hat{Y}}_{1}}P_{Y_{2}|{\hat{Y}}_{2}}\;\;then\;inequality\;(220)\;holds\;with\;equality, \end{array}$$

(227) $$if\;\;E\left[Y_{1}|F^{{\hat{Y}}_{1}}\right]={\hat{Y}}_{1}\;\;then\;inequality\;(224)\;holds\;with\;equality,$$

(228) $$\begin{array}{cc} \; & if\;\;E\left[Y_{2}|F^{{\hat{Y}}_{2}}\right]={\hat{Y}}_{2}\;\;then\;inequality\;(225)\;holds\;with\;equality. \end{array}$$

It can be verified that the representations (196)–(209) satisfy $P_{Y_{1},Y_{2}|{\hat{Y}}_{1},{\hat{Y}}_{2}}=P_{Y_{1}|{\hat{Y}}_{1}}P_{Y_{2}|{\hat{Y}}_{2}}$, $E\left[Y_{1}|F^{{\hat{Y}}_{1}}\right]={\hat{Y}}_{1}$, $E\left[Y_{2}|F^{{\hat{Y}}_{2}}\right]={\hat{Y}}_{2}$, and that all inequalities become equalities. The decomposition of the joint distribution according to (210) follows from the representations of $\left({\hat{Y}}_{1},{\hat{Y}}_{2}\right)$, and similarly for (211) and (112). The conditional RDFs $R_{Y_{i}|W}\left(\Delta_{i}\right),i=1,2$ are shown as above. (c) This is easily verified, because (210) holds and hence rates lie on the Pangloss plane, for the strictly positive surface, $D_{C}\left(Y_{1},Y_{2}\right)$. (d) This follows directly from parts (a)–(c). □

**Remark** **8.** *We should emphasize that Theorem 16 does not fully characterize the Pangloss plane, i.e., the subset of the rate regions such that $R_{Y_{1}|W}\left(\Delta_{1}\right)+R_{Y_{2}|W}\left(\Delta_{2}\right)+I\left(Y_{1},Y_{2};W\right)=R_{Y_{1},Y_{2}}\left(\Delta_{1},\Delta_{2}\right)$ is larger than $D_{C}\left(Y_{1},Y_{2}\right)$. To determine the entire set that characterizes the Pangloss plane, we need to consider the rate distortion function (177) with (178), and the general realization (40). We do not pursue this further, because it requires the closed-form solution of $R_{Y_{1},Y_{2}}\left(\Delta_{1},\Delta_{2}\right)$, which is currently an open and challenging problem, and beyond the scope of this paper. We should mention that the analysis of the scalar-valued Gaussian example in [12,13], i.e., when $p_{1}=p_{2}=1$, made use of closed-form expression of $R_{Y_{1},Y_{2}}\left(\Delta_{1},\Delta_{2}\right)$ due to [13].*

**Proof**  (Proof of Theorem 6). One way to prove the statement is to compute the characterizations of the rate distortion functions $R_{Y_{i}}\left(\Delta_{i}\right),R_{Y_{i}|W}\left(\Delta_{i}\right),i=1,2$ and $R_{Y_{1},Y_{2}}\left(\Delta_{1},\Delta_{2}\right)$, using the realization of the random variables $\left(Y_{1},Y_{2}\right)$ which induce the family of measures $P_{\mathrm{ci}}\subseteq P_{min}^{CIG}$, as defined in Corollary 1, by (84)–(88). In view of Definition 7. (b), it suffices to verify that identity (15) holds, i.e., $R_{Y_{1}|W}\left(\Delta_{1}\right)+R_{Y_{2}|W}\left(\Delta_{2}\right)+I\left(Y_{1},Y_{2};W\right)=R_{Y_{1},Y_{2}}\left(\Delta_{1},\Delta_{2}\right)$ for $\left(\Delta_{1},\Delta_{2}\right)\in D_{W}$, for the choice $W=W^{*}\in G\left(0,I\right)$ which achieves the minimum in (188) (i.e., due to Theorem 11. (b)).

Similar to Theorem 16, it can be shown that the conditional RDFs $R_{Y_{i}|W^{*}}\left(\Delta_{i}\right),i=1,2$ are given by

(229) $$R_{Y_{1}|W^{*}}\left(\Delta_{1}\right)=\underset{\sum_{j=1}^{n}\Delta_{1,j}\leq \Delta_{1}}{\inf}\frac{1}{2}\sum_{j=1}^{n}\ln\left(\frac{\left(1-d_{j}\right)}{\Delta_{1,j}}\right),\;\;W^{*}\in G\left(0,I\right)$$

(230) $$R_{Y_{2}|W^{*}}\left(\Delta_{2}\right)=\underset{\sum_{j=1}^{n}\Delta_{2,j}\leq \Delta_{1}}{\inf}\frac{1}{2}\sum_{j=1}^{n}\ln\left(\frac{\left(1-d_{j}\right)}{\Delta_{2,j}}\right),\;\;W^{*}\in G\left(0,I\right),$$

(231) $$\begin{array}{cc} \; & E||Y_{1}-{\hat{Y}}_{1}{\left|\right|}_{R^{n}}^{2}=\sum_{j=1}^{n}\Delta_{1,j},\;\;\;\;E||Y_{2}-{\hat{Y}}_{2}{\left|\right|}_{R^{n}}^{2}=\sum_{j=1}^{n}\Delta_{2,j}. \end{array}$$

The pay-off of the joint RDF $R_{Y_{1},Y_{2}}\left(\Delta_{1},\Delta_{2}\right)$ in (190) is related to the pay-offs of the conditional RDFs $R_{Y_{1}|W}\left(\Delta_{1}\right),R_{Y_{2}|W}\left(\Delta_{2}\right)$, and $C\left(Y_{1},Y_{2}\right)=I\left(Y_{1},Y_{2};W^{*}\right)$ in (102), via the identity

(232) $$\frac{1}{2}\sum_{j=1}^{n}\ln\left(\frac{\left(1-d_{j}^{2}\right)}{\Delta_{1,j}\Delta_{2,j}}\right)=\frac{1}{2}\sum_{j=1}^{n}\ln\left(\frac{\left(1-d_{j}\right)}{\Delta_{1,j}}\right)+\frac{1}{2}\sum_{j=1}^{n}\ln\left(\frac{\left(1-d_{j}\right)}{\Delta_{2,j}}\right)+\frac{1}{2}\sum_{i=1}^{n}\ln\left(\frac{1+d_{i}}{1-d_{i}}\right).$$

For $\left(\Delta_{1},\Delta_{2}\right)\in D_{W}$ defined by (47), it then follows from (232), the identity

(233) $$R_{Y_{1},Y_{2}}\left(\Delta_{1},\Delta_{2}\right)=\underset{\sum_{j=1}^{n}\Delta_{1,j}\leq \Delta_{1},\sum_{j=1}^{n}\Delta_{2,j}\leq \Delta_{2}}{\inf}\frac{1}{2}\sum_{j=1}^{n}\ln\left(\frac{\left(1-d_{j}^{2}\right)}{\Delta_{1,j}\Delta_{2,j}}\right)$$

(234) $$=R_{Y_{1}|W^{*}}\left(\Delta_{1}\right)+R_{Y_{2}|W^{*}}\left(\Delta_{2}\right)+I\left(Y_{1},Y_{2};W^{*}\right),\;\;\left(\Delta_{1},\Delta_{2}\right)\in D_{W}\;\;defined\;by\;(47).$$

This completes the proof. □

### 4.3. Applications to Problems of the Literature [15,16,17]

The next two corollaries illustrate the application of the results developed in this paper to the optimization problems analyzed in [15,16,17].

**Corollary** **3.** *Applications to problems in [15]*

*Consider the Gaussian secure source coding and Wyner’s common information [15], defined by the optimization problem [15] (see Equation (18), Section IV.B),*

(235) $$\begin{array}{c} \arg\min_{P_{Y_{1},Y_{2},W}:P_{Y_{1},Y_{2}|W}=P_{Y_{1}|W}P_{Y_{2}|W}}\left\{\lambda I\left(Y_{1};W\right)+I\left(Y_{1},Y_{2};W\right)\right\},\;\;\lambda \in \left[0,\infty \right) \end{array}$$

*where the tuple $\left(Y_{1},Y_{2}\right)$ are the zero mean jointly Gaussian, and W:Ω→W is continuous or discrete-valued random variable (The derivation of the formula for (235) in [15] makes use of rate distortion functions, [15] (Equation (47))).*

*Then, the following hold.*

*For any jointly distributed random variables $\left(Y_{1},Y_{2},W\right)$ that minimize the expression in (235), there exists a jointly Gaussian triple $\left(Y_{1},Y_{2},W\right)$ such that $W:\to R^{n}$ is a Gaussian random variable, which achieves the same minimum value.*

*Moreover, the following characterization of (235) holds.*

(236) $$\begin{array}{cc} \; & \arg\min_{P_{Y_{1},Y_{2},W}:P_{Y_{1},Y_{2}|W}=P_{Y_{1}|W}P_{Y_{2}|W}}\left\{\lambda I\left(Y_{1};W\right)+I\left(Y_{1},Y_{2};W\right)\right\}\\ \; & =\;\;\arg\min_{Q_{W}\in Q_{W}:Q_{W}defined\;by\;\left(82\right)}\{\frac{\lambda }{2}\ln\frac{1}{\det(I-D^{1/2}Q_{W}D^{1/2})}+\frac{1}{2}\sum_{i=1}^{n}\ln\left(1-d_{i}^{2}\right) \end{array}$$

(237) $$\begin{array}{c} \;\;\;\;-\frac{1}{2}\ln\left(\det\left(\left[I-D^{1/2}D_{W}^{-1}D^{1/2}\right]\left[I-D^{1/2}D_{W}D^{1/2}\right]\right)\right)\}\\ =\;\;\arg\min_{Q_{W}=D_{W}\in Q_{W}:Q_{W}defined\;by\;\left(82\right)}\{\frac{\lambda }{2}\sum_{i=1}^{n}\ln\left({\left[1-\frac{d_{i}}{q_{i}}\right]}^{-1}\right)+\frac{1}{2}\sum_{i=1}^{n}\ln\left(1-d_{i}^{2}\right)\\ \;\;\;\;-\frac{1}{2}\ln\left(\left[1-\frac{d_{i}}{q_{i}}\right]\left[1-q_{i}d_{i}\right]\right)\} \end{array}$$

*where*

(238) $$\begin{array}{c} Q_{W}=D_{W}=\mathrm{Diag}\left(q_{1},q_{2},\ldots ,q_{n}\right)\in Q_{W},\;\;q_{1}\geq q_{2}\geq \ldots \geq q_{n}>0,\;\;Q_{W}=\left(82\right). \end{array}$$

**Proof.**  By the use Theorem 9. (c), it suffices to restrict attention to jointly Gaussian random variables $\left(Y_{1},Y_{2},W\right)$. Transform the tuple $\left(Y_{1},Y_{2}\right)$ in the canonical variable form of Definition 1. Restrict attention to the correlated parts of these random variables, as defined in Theorem 8, by (77)–(79), and consider the realization of the transformed random variables of Corollary 1. Then, the value of $I\left(Y_{1};W\right)+I\left(Y_{2},Y_{2};W\right)$ is identical to the value of the same expression, evaluated using the realization of Corollary 1. By simple evaluation, using the realization of Corollary 1,

(239) $$\begin{array}{cc} \lambda I(Y_{1};W) & +I\left(Y_{2},Y_{2};W\right)=\frac{\lambda }{2}\ln\frac{1}{\det(I-D^{1/2}Q_{W}^{-1}D^{1/2})}\\ \; & +\frac{1}{2}\sum_{i=1}^{n}\ln\left(1-d_{i}^{2}\right)-\frac{1}{2}\ln\left(\det\left(\left[I-D^{1/2}Q_{W}^{-1}D^{1/2}\right]\left[I-D^{1/2}Q_{W}D^{1/2}\right]\right)\right) \end{array}$$

and it is parameterized by $Q_{W}\in Q_{W}$, where $Q_{W}$ is defined by the set of Equation (82). By Hadamard’s determinant inequality, an achievable lower bound on the first right-hand side term of (239), holds if $Q_{W}\in Q_{W}$ and $\left(I-D^{1/2}Q_{W}^{-1}D^{1/2}\right)$ is a diagonal matrix, and this lower bound is achieved by a diagonal $Q_{W}\in Q_{W}$. Furthermore, by recalling the derivation of Theorem 11, an achievable lower bound on the second right-hand side term of (239) holds, i.e., of $I(Y_{1},Y_{2};W)$, when $Q_{W}\in Q_{W}$ is diagonal. Hence, both lower bounds are achieved simultaneously, by $Q_{W}\in Q_{W}$ and $Q_{W}$ a diagonal matrix. Then, an achievable lower bound on (239) is obtained, if $Q_{W}$ is specified by (238). □

The remaining optimization problem in (237) is easily carried out, and hence omitted.

Corollary 4 illustrates the application of the results developed in this paper to the Gaussian relaxed Wyner’s common information [16,17] (Definition 2 and Section III).

**Corollary** **4.** *Applications to problems in [16,17]*

*Consider the Gaussian relaxed Wyner’s common information considered in [16,17] (see Definition 2 and Section III of [17])*

(240) $$\begin{array}{cc} C_{\gamma }\left(Y_{1},Y_{2}\right)= & \min_{P_{W|Y_{1},Y_{2}}:\;I\left(Y_{1};Y_{2}|W\right)\leq \gamma }I\left(Y_{1},Y_{2};W\right) \end{array}$$

*where the tuple $\left(Y_{1},Y_{2}\right)$ are zero mean jointly Gaussian, and W:Ω→W is continuous or discrete-valued random variable (the value of (240) computed in [16,17], Theorem 4, is different from (241); moreover, the derivation in [16,17], Section III.A, is different from the derivation presented below). Then*

(241) $$\begin{array}{c} C_{\gamma }\left(Y_{1},Y_{2}\right)=C\left(Y_{1},Y_{2}\right)=\left(19\right),\;\;\;\forall \gamma \in \left(0,\infty \right). \end{array}$$

**Proof.**  By the use Theorem 9. (c), it suffices to restrict the attention to jointly Gaussian random variables $\left(Y_{1},Y_{2},W\right)$. By Proposition 3 or Corollary 1, there exists a family of realizations of $\left(Y_{1},Y_{2}\right)$ parameterized by a Gaussian random variable *W*, which induces conditional independence $P_{Y_{1},Y_{2}|W}=P_{Y_{1}|W}P_{Y_{2}|W}$, and hence the lower bound $I\left(Y_{1},Y_{2};W\right)\geq H\left(Y_{1},Y_{2}\right)-H\left(Y_{1}|W\right)-H\left(Y_{2}|W\right)$ is achieved, i.e., the constraint in (240) is always satisfied, because the minimizer is such that $I(Y_{1};Y_{2}|W)=0$, i.e., the constraint is not active. Hence, the general solution of (240) is the one given in Theorem 4. □

**Remark** **9.** *Corollary 4 implies that the definition of the relaxed Gaussian Wyner’s common information considered in [16,17] (see Definition 2 and Section III of [17]) should be replaced by $\min_{P_{W|Y_{1},Y_{2}}:\;I\left(Y_{1};Y_{2}|W\right)=\gamma }I\left(Y_{1},Y_{2};W\right)$, i.e., the inequality is replaced by an equality, so that the constraint is active for all γ∈(0,∞).*

### 4.4. Characterization and Parameterization of the Gray and Wyner Rate Region by Jointly Gaussian RVs

Derived in this section, for jointly Gaussian random variables with square-error distortion, using [1] ((4) of page 1703, Equation (42)), i.e., (12), and the RDFs, $R_{Y_{1},Y_{2}}\left(\Delta_{1},\Delta_{2}\right),$$R_{Y_{i}|W}\left(\Delta_{i}\right)$, $R_{Y_{i}}\left(\Delta_{i}\right),i=1,2$, of Theorems 12, 13, 14, and Theorem 9, are:

(1) Theorem 5—the characterizations of the rate region $R_{GW}\left(\Delta_{1},\Delta_{2}\right)$, and
(2) The characterization of rates that lie on Pangloss Plane.

**Proof**  (Proof of Theorem 5). (a) This follows from the realization of random variables that induce Gaussian measures, by repeating Theorem 9, without requiring *W* makes $Y_{1}$ and $Y_{2}$ conditionally independent, i.e., the stated realization with random variables $Z_{1}$ and $Z_{2}$ correlated, achieves a lower bound on $I(Y_{1},Y_{2};W)$, among all random variables *W*. (b) The stated characterization (42) follows from the discussion prior to the Theorem, i.e., by an application of [1] ((4) of page 1703, Equation (42)), i.e., $T(\alpha_{1},\alpha_{2})$, and Theorem 9, Theorem 13, which imply that the infimum in $T(\alpha_{1},\alpha_{2})$ is over the parameterized set of jointly Gaussian random variables $\left(Y_{1},Y_{2},W\right)\in G\left(0,Q_{\left(Y_{1},Y_{2},W\right)}\right)$ with joint distribution (68). From part (a), then (43) follows. (44) is due to identity $I\left(Y_{1},Y_{2};W\right)=H\left(Y_{1},Y_{2}\right)-H\left(Y_{1}|Y_{2},W\right)-H\left(Y_{2}|W\right)$ and that the values $R_{Y_{1}|W}\left(\Delta_{1}\right)$ and $R_{Y_{2}|W}\left(\Delta_{2}\right)$ depend only on $Q_{Y_{1}|W}$ and $Q_{Y_{2}|W}$, and the errors (see Theorem 13). □

Theorem 17 gives the parameterization of a subset of the Pangloss Plane, as a degenerate case of Theorem 5.

**Theorem** **17.** *Consider the statement of Theorem 5.*

*(a) Rate triples $\left(R_{0},R_{1},R_{2}\right)$ that lie on the Pangloss Plane, is determined by the subset of the rate region $R_{GW}\left(\Delta_{1},\Delta_{2}\right)$ of Theorem 5. (b), such that the joint distribution $P_{W,Y_{1},Y_{2},{\hat{Y}}_{1},{\hat{Y}}_{2}}$ satisfies the conditions,*

(242) $$\begin{array}{c} \;P_{{\hat{Y}}_{1},{\hat{Y}}_{2}|W}=P_{{\hat{Y}}_{1}|W}P_{{\hat{Y}}_{2}|W},\;\;P_{Y_{1},Y_{2}|{\hat{Y}}_{1},{\hat{Y}}_{2},W}=P_{Y_{1},Y_{2}|{\hat{Y}}_{1},{\hat{Y}}_{2}}. \end{array}$$

*Specifically, the Pangloss Plane is characterized by*

$$\begin{array}{cc} T^{G}\left(\alpha_{1},\alpha_{2}\right)= & \underset{\left(Y_{1},Y_{2},W\right)\in G(0,Q_{\left(Y_{1},Y_{2},W\right)}\;of(30),(40)}{\inf}\{I\left(Y_{1},Y_{2};W\right) \end{array}$$

(243) $$\begin{array}{cc} \; & +\alpha_{1}R_{Y_{1}|W}\left(\Delta_{1}\right)+\alpha_{2}R_{Y_{2}|W}\left(\Delta_{2}\right)\}\\ = & \underset{Q_{Y_{1}|Y_{2},W},Q_{Y_{2}|W}}{\inf}\{\frac{1}{2}\ln\frac{\det(Q_{\left(Y_{1},Y_{2}\right)})}{\det\left(Q_{Y_{1}|Y_{2},W}\right)\det\left(Q_{Y_{2}|W}\right)} \end{array}$$

(244) $$+\alpha_{1}R_{Y_{1}|W}\left(\Delta_{1}\right)+\alpha_{2}R_{Y_{2}|W}\left(\Delta_{2}\right)\}$$

*such that*

(245) $$\begin{array}{c} P_{W,Y_{1},Y_{2},{\hat{Y}}_{1},{\hat{Y}}_{2}}satisfies\left(242\right),and\;its\;marginals\;induce\;the\;test\;channels\\ oftheRDFs,R_{Y_{1},Y_{2}}\left(\Delta_{1},\Delta_{2}\right),R_{Y_{i}|W}\left(\Delta_{i}\right),R_{Y_{i}}\left(\Delta_{i}\right),i=1,2,of\;Theorems\;12,\;13,\;14. \end{array}$$

*(b) A subset of the rate triple $\left(R_{0},R_{1},R_{2}\right)$ that lie on the Pangloss Plane is determined by the restriction of part (a) to $(Y_{1},Y_{2}|W)\in \mathrm{CIG}$, i.e.,*

$$\begin{array}{cc} T^{GCI}\left(\alpha_{1},\alpha_{2}\right)= & \underset{\left(Y_{1},Y_{2},W\right)\in G(0,Q_{\left(Y_{1},Y_{2},W\right)},\;\left(Y_{1},Y_{2}|W\right)\in \mathrm{CIG}}{\inf}\{I\left(Y_{1},Y_{2};W\right) \end{array}$$

(246) $$\begin{array}{cc} \; & +\alpha_{1}R_{Y_{1}|W}\left(\Delta_{1}\right)+\alpha_{2}R_{Y_{2}|W}\left(\Delta_{2}\right)\}\\ = & \underset{Q_{Y_{1}|W},Q_{Y_{2}|W}}{\inf}\{\frac{1}{2}\ln\frac{\det(Q_{\left(Y_{1},Y_{2}\right)})}{\det\left(Q_{Y_{1}|W}\right)\det\left(Q_{Y_{2}|W}\right)} \end{array}$$

(247) $$+\alpha_{1}R_{Y_{1}|W}\left(\Delta_{1}\right)+\alpha_{2}R_{Y_{2}|W}\left(\Delta_{2}\right)\}$$

*such that (245) holds, where $I\left(Y_{1},Y_{2};W\right)=H\left(Y_{1},Y_{2}\right)-H\left(Y_{1}|W\right)-H\left(Y_{2}|W\right)$, and $R_{Y_{i}|W}\left(\Delta_{i}\right),$i=1,2 are given in Theorem 13. (c).*

**Proof.**  (a) Condition (242) characterizes the rates $\left(R_{0},R_{1},R_{2}\right)\in R_{GW}\left(\Delta_{1},\Delta_{2}\right)$ that lie on the Pangloss plane, and these are derived in [12] (Theorem 1, Equations (19) and (20)). Hence, the statement follows from Theorem 5.

(b) That (246) defines a subset of $R_{GW}\left(\Delta_{1},\Delta_{2}\right)$, follows from the fact that the set of joint distributions $\left(Y_{1},Y_{2},W\right)\in G(0,Q_{\left(Y_{1},Y_{2},W\right)}$ and $(Y_{1},Y_{2}|W)\in \mathrm{CIG}$ is a subset of the set of joint distributions $\left(Y_{1},Y_{2},W\right)\in G\left(0,Q_{\left(Y_{1},Y_{2},W\right)}\right)$. Moreover, by part (a) and $(Y_{1},Y_{2}|W)\in \mathrm{CIG}$ implies $I\left(Y_{1},Y_{2};W\right)=H\left(Y_{1},Y_{2}\right)-H\left(Y_{1}|Y_{2},W\right)-H\left(Y_{2}|W\right)=H\left(Y_{1},Y_{2}\right)-H\left(Y_{1}|W\right)-H\left(Y_{2}|W\right)$ and that the values $R_{Y_{1}|W}\left(\Delta_{1}\right)$ and $R_{Y_{2}|W}\left(\Delta_{2}\right)$ only depend on $Q_{Y_{1}|W}$ and $Q_{Y_{2}|W}$, and the errors, as shown in Theorem 13. (c). Hence, the statement holds. □

From Theorem 16 and Theorem 17. (b) follows a simpler parameterization of rates that lie on the Pangloss Plane of the Gray–Wyner rate region $R_{GW}\left(\Delta_{1},\Delta_{2}\right)$, when $\left(Y_{1},Y_{2}\right)$ are in canonical variable form.

**Corollary** **5.** *Consider the statement of Theorem 16 with square error distortion functions, i.e., a tuple $\left(Y_{1},Y_{2}\right)$ is the canonical variable form.*

*A subset of the rate triple $\left(R_{0},R_{1},R_{2}\right)$ that lie on the Pangloss Plane corresponding to the restriction $(Y_{1},Y_{2}|W)\in \mathrm{CIG}$, is determined from*

(248) $$\begin{array}{cc} T_{\mathrm{cvf}}^{CIG}\left(\alpha_{1},\alpha_{2}\right)= & \underset{Q_{W}}{\inf}\left\{I\left(Y_{1},Y_{2};W\right)+\alpha_{1}R_{Y_{1}|W}\left(\Delta_{1}\right)+\alpha_{2}R_{Y_{2}|W}\left(\Delta_{2}\right)\right\}\\ = & \underset{Q_{W}}{\inf}\{\frac{1}{2}\sum_{i=1}^{n}\ln\left(1-d_{i}^{2}\right)-\frac{1}{2}\ln\left(\det\left(\left[I-D^{1/2}Q_{W}^{-1}D^{1/2}\right]\left[I-D^{1/2}Q_{W}D^{1/2}\right]\right)\right) \end{array}$$

(249) $$+\alpha_{1}R_{Y_{1}|W}\left(\Delta_{1}\right)+\alpha_{2}R_{Y_{2}|W}\left(\Delta_{2}\right)\}$$

*such that (245) holds, where $0\leq \alpha_{i}\leq 1,i=1,2,\alpha_{1}+\alpha_{2}\geq 1$, and where $R_{Y_{i}|W}\left(\Delta_{i}\right),i=1,2$ are given in Theorem 16, and the infimum is taken over $Q_{W}\in Q_{W}$, defined by the set of Equation (82).*

**Proof.**  The stated characterization (248) is the application of [1] ((4) of page 1703, Equation (42)) and the results of Theorem 16 and Theorem 17. (b). □

In view of Theorem 1 (i.e., Theorem 8 in [1]), additional parameterizations of the Gray–Wyner rate region $R_{GW}\left(\Delta_{1},\Delta_{2}\right)$ directly follow from the expressions already derived, i.e., the joint RDF $R_{Y_{1},Y_{2}}\left(\Delta_{1},\Delta_{2}\right)$ of Theorem 12, the conditional RDFs $R_{Y_{1}|W}\left(\Delta_{1}\right),R_{Y_{2}|W}\left(\Delta_{2}\right)$ of Theorem 13, the maginal RDFs $R_{Y_{1}}\left(\Delta_{1}\right),R_{Y_{2}}\left(\Delta_{2}\right)$ of Theorem 14, and the values of $I(Y_{1},Y_{2};W)$, based on Gaussian realization of Theorem 5. (a).

## 5. Conclusions

This paper formulates the classical Gray and Wyner source coding for a simple network with a tuple of multivariate, correlated Gaussian random variables, with square-error fidelity at the two decoders, from the geometric approach of a Gaussian random variables and the weak stochastic realization of correlated Gaussian random variables. This approach leads to a parameterization of the Gray–Wyner rate region, with respect to variance matrix of the jointly Gaussian triple $\left(Y_{1},Y_{2},W\right)$, where *W* a Gaussian auxiliary random variable. However, much remains to be achieved, from the computation point of view, for this problem, and to exploit the new approach to other multi-user problems of information theory.

## Data Availability

Numerical evaluations of Wyner’s common information, based on the implementation of the canonical variable form, and the calculation of the canonical variable coefficients are found in [21], Section 3.7.

## Appendix A

### Appendix A.1. Algorithm to Generate the Canonical Variable Form

The algorithm that generates $Q_{\mathrm{cvf}}$ is presented below.

Transformation of a variance matrix into its canonical variable form.

Data: $p_{1},p_{2}\in Z_{+}$, $Q\in R^{\left(p_{1}+p_{2}\right)\times \left(p_{1}+p_{2}\right)}$, satisfying $Q=Q^{T}\geq 0$ (it is noted that Q>0 implies $Q_{11}>0,$$Q_{22}>0$), with decomposition

$$\begin{array}{c} Q=\left(\begin{array}{cc} Q_{11} & Q_{12}\\ Q_{12}^{T} & Q_{22} \end{array}\right),\;\;Q_{11}\in R^{p_{1}\times p_{1}},\;Q_{22}\in R^{p_{2}\times p_{2}},\;Q_{12}\in R^{p_{1}\times p_{2}},\;\;Q_{11}>0,Q_{22}>0. \end{array}$$

1 Perform singular-value decompositions:
  $$Q_{11}\;=\;U_{1}D_{1}U_{1}^{T},\;Q_{22}\;=\;U_{2}D_{2}U_{2}^{T},$$
  with $U_{1}\in R^{p_{1}\times p_{1}}$ orthogonal ($U_{1}U_{1}^{T}=I=U_{1}^{T}U_{1}$) and
  $$D_{1}=\mathrm{Diag}\left(d_{1,1},\ldots ,d_{1,p_{1}}\right)\in R^{p_{1}\times p_{1}},\;d_{1,1}\geq d_{1,2}\geq \ldots \geq d_{1,p_{1}}>0,$$
  and $U_{2},D_{2}$ satisfying corresponding conditions.
2 Perform a singular-value decomposition of
  $$D_{1}^{-\frac{1}{2}}U_{1}^{T}Q_{12}U_{2}D_{2}^{-\frac{1}{2}}\;=\;U_{3}D_{3}U_{4}^{T},$$
  with $U_{3}\in R^{p_{1}\times p_{1}}$, $U_{4}\in R^{p_{2}\times p_{2}}$ orthogonal and
  $$\begin{array}{cc} D_{3}= & \left(\begin{array}{ccc} I_{p_{11}} & 0 & 0\\ 0 & D_{4} & 0\\ 0 & 0 & 0 \end{array}\right)\in R^{p_{1}\times p_{2}},\\ D_{4}= & \mathrm{Diag}\left(d_{4,1},\ldots ,d_{4,p_{12}}\right)\in R^{p_{12}\times p_{12}},\;\;1>d_{4,1}\geq d_{4,2}\geq \ldots \geq d_{4,p_{12}}>0. \end{array}$$
3 Compute the new variance matrix according to
  $$\begin{array}{c} Q_{\mathrm{cvf}}=\left(\begin{array}{cc} I_{p_{1}} & D_{3}\\ D_{3}^{T} & I_{p_{2}} \end{array}\right). \end{array}$$
4 The transformation to the canonical variable representation
  $\left(Y_{1}\mapsto S_{1}Y_{1},\;Y_{2}\mapsto S_{2}Y_{2}\right)$ is then
  $$S_{1}\;=\;U_{3}^{T}D_{1}^{-\frac{1}{2}}U_{1}^{T},\;S_{2}\;=\;U_{4}^{T}D_{2}^{-\frac{1}{2}}U_{2}^{T}.$$

If Q>0 then $Q_{11}>0$ and $Q_{22}>0$, and $D_{3}$ does not contain the first row and first column, i.e., these are removed.

### Appendix A.2. Information Theory

In this appendix, the reader finds two formulas of information theory which are used in the body of the paper. These are obtained from [29,30,31].

The first equality is proven in [29] (p. 19, Th. 2.4.1) and [30] (p. 31, (2.4.26), (2.4.28)).

**Proposition** **A1.**
*Consider random variables $Y_{1,1},Y_{1,2},Y_{2,1},Y_{2,2},X_{1},X_{2}$ such that the following two triples are independent random variables, $\left(Y_{1,1},Y_{2,1},X_{1}\right)$ and $\left(Y_{1,2},Y_{2,2},X_{2}\right)$. Then, the mutual information expression additively decomposes,*

(A1)
$$\begin{array}{c} I\left(Y_{1,1},Y_{1,2},Y_{2,1},Y_{2,2};X_{1},X_{2}\right)=I\left(Y_{1,1},Y_{2,1};X_{1}\right)+I\left(Y_{1,2},Y_{2,2};X_{2}\right). \end{array}$$

**Proof.** For completeness, the proof is given in [21] (Proposition A.2). □

Consider a tuple of jointly Gaussian random variables $\left(X,Y\right)\in G\left(0,Q_{\left(X,Y\right)}\right)$ with $X:\Omega \to R^{n}$, $Y:\Omega \to R^{p}$, $Q_{\left(X,Y\right)}>0$, and $Q_{\left(X,Y\right)}=\left(\begin{array}{cc} Q_{X} & Q_{X,Y}\\ Q_{X,Y}^{T} & Q_{Y} \end{array}\right)$. Then,

(A2) $$\begin{array}{cc} H(X)= & \frac{1}{2}\ln\left(\det(Q_{X})\right)+\frac{1}{2}n\ln\left(2\pi e\right), \end{array}$$

(A3) $$\begin{array}{cc} H(Y|X)= & H\left(X,Y\right)-H\left(X\right)=\frac{1}{2}\ln\left(\det(Q_{Y}-Q_{X,Y}^{T}Q_{X}^{-1}Q_{X,Y})\right)+\frac{1}{2}p\ln\left(2\pi e\right), \end{array}$$

(A4) $$\begin{array}{cc} I(Y;X)= & -\frac{1}{2}\ln\left(\frac{\det(Q_{\left(X,Y\right)})}{\det\left(Q_{Y}\right)\det\left(Q_{X}\right)}\right). \end{array}$$

### Appendix A.3. An Inequality for Determinants

An inequality for matrices is derived in this appendix which is needed in the body of the paper.

**Lemma** **A1.**
*Consider the real-valued matrices $A,\;B\in R^{n\times n}$. Assume that*

(A5)
$$\begin{array}{ccc} 0 & \leq & I-A^{T}A,\;\;0<I-B^{T}B,\;and\;\mathrm{rank}\left(B\right)=n. \end{array}$$

*Then,*

(A6)
$$\begin{array}{ccc} & & \det\left(\left[I-A^{T}A\right]\left[I-B^{T}B\right]\right)\leq {\left(\det\left(I-A^{T}B\right)\right)}^{2}. \end{array}$$

A related result is mentioned at [32] (Theorem 9.E.6). In that book, the proof of corresponding result refers to paper [33]. That reference was received by the authors but they could not read it because the paper is in Mandarin. However, they could read the formulas of the paper. Hua LooKeng developed these results to calculate an orthonormal basis for a function of one complex variable. A more recent reference for this inequality is [34] (Theorem 7.19).

The proof of Lemma A1 below is analogous to that of Hua LooKeng in [33]. The main differences are in the assumptions.

**Lemma** **A2.**
*Ref. [33] (pp. 464, 470). Consider the matrices $A,B\in R^{n\times n}$. Assume that $I-B^{T}B$ is a nonsingular matrix and that rank(B)=n. Then*

(A7)
$$\begin{array}{ccc} & & \left(I-A^{T}A\right)-\left(I-A^{T}B\right){\left[I-B^{T}B\right]}^{-1}{\left(I-A^{T}B\right)}^{T}\\ & = & -\left(A-B\right){\left[I-B^{T}B\right]}^{-1}{\left(A-B\right)}^{T}. \end{array}$$

**Proof.** For completeness, the proof is given in [21] (Lemma A.4). □

**Proposition** **A2.**
*Ref. [33] (Equation (2)). Consider the symmetric positive-definite matrices $Q_{1},\;Q_{2},$$Q\in R^{n\times n}$ such that $Q_{1}+Q_{2}=Q$. Then*

(A8)
$$\begin{array}{c} \det\left(Q_{1}\right)+\det\left(Q_{2}\right)\leq \det\left(Q\right). \end{array}$$

**Proof. Proof of Lemma** **A1.** By the assumptions, $0\leq (I-A^{T}A)$, $0<(I-B^{T}B)$, and rank(B)=n, from Lemma A2 follows that

$$\begin{array}{ccc} & & \left(A-B\right){\left[I-B^{T}B\right]}^{-1}{\left(A-B\right)}^{T}+\left[I-A^{T}A\right]\\ & = & \left(I-A^{T}B\right){\left[I-B^{T}B\right]}^{-1}{\left(I-A^{T}B\right)}^{T};\;\\ & & 0\leq \det(I-A^{T}A),\;by\;the\;assumptionon\;A,\\ & \leq & \det\left(\left(A-B\right){\left[I-B^{T}B\right]}^{-1}{\left(A-B\right)}^{T}\right)+\det\left(\left[I-A^{T}A\right]\right),by\;an\;assumptionon\;B,\\ & \leq & \det(\left(I-A^{T}B\right){\left[I-B^{T}B\right]}^{-1}{\left(I-A^{T}B\right)}^{T})\;\\ & & by\;Lemma\;,Proposition\;,and\;by\;the\;assumptions,\\ & = & {\left(\det(I-A^{T}B)\right)}^{2}{\left[\det\left(\left[I-B^{T}B\right]\right)\right]}^{-1},\\ & \Rightarrow & \det\left(\left[I-A^{T}A\right]\left[I-B^{T}B\right]\right)=\det\left(\left[I-A^{T}A\right]\right)\det\left(\left[I-B^{T}B\right]\right)\leq {\left(\det(\left[I-A^{T}B\right])\right)}^{2}. \end{array}$$

□

Another preliminary result is needed.

**Proposition** **A3.**
*Consider the matrix $Q_{X}=Q_{X}^{T}\in R^{n\times n}$ of Proposition 2 and the matrix $D\in R^{n\times n}$ of Definition 1. Thus, both $Q_{X}>0$ and D>0. Then,*

(A9)
$$\begin{array}{ccc} & & D\leq Q_{X}^{-1}\leq D^{-1}\;\;\Leftrightarrow \;\;D\leq Q_{X}\leq D^{-1}, \end{array}$$

(A10)
$$\begin{array}{ccc} & & D<Q_{X}^{-1}<D^{-1}\;\;\Leftrightarrow \;\;D<Q_{X}<D^{-1}. \end{array}$$

**Proof.** For completeness, the proof is given in [21] (Proposition A.6). □

**Proposition** **A4.**
*Consider the matrices defined in Definition 1. Thus, $D\in R^{n\times n}$, is a diagonal matrix satisfying 0<D, and the matrix $Q_{X}\in R^{n\times n}$ satisfies $Q_{X}=Q_{X}^{T}$ and $0<D\leq Q_{X}\leq D^{-1}$. Then,*

(A11)
$$\begin{array}{ccc} & & \det\left(\left[I-D^{1/2}Q_{X}^{-1}D^{1/2}\right]\left[I-D^{1/2}Q_{X}D^{1/2}\right]\right)\leq \det\left({\left[I-D\right]}^{2}\right),\;\forall \;Q_{X}, \end{array}$$

(A12)
$$\begin{array}{ccc} & & \det\left(\left[I-D^{1/2}Q_{X}^{-1}D^{1/2}\right]\left[I-D^{1/2}Q_{X}D^{1/2}\right]\right)<\det\left({\left[I-D\right]}^{2}\right),\;if\;Q_{X}\neq I. \end{array}$$

**Proof.** (1) If $Q_{X}<D^{-1}$ then $\det(D^{-1}-Q_{X})>0$. Consider the case of in which $Q_{X}$ satisfies $Q_{X}\leq D^{-1}$ but not $Q_{X}<D^{-1}$. Then, $\det(D^{-1}-Q_{X})=0$. Hence,

$$\begin{array}{cc} \det(I-D^{1/2}Q_{X}D^{1/2})=\; & \det(D^{1/2}\left[D^{-1}-Q_{X}\right]D^{1/2})\\ = & \det\left(D^{1/2}\right)\det\left(D^{-1}-Q_{X}\right)\det\left(Q^{1/2}\right)=0. \end{array}$$

Then, 0<D<I implies that $\det({\left[I-D\right]}^{2})\geq 0$, hence that the inequality (A11) holds.

(2) If $D<Q_{X}$ then $\det(D-Q_{X})<0$. If $D\leq Q_{X}$ but not $D<Q_{X}$ then by Proposition A3 $Q_{X}^{-1}\leq D^{-1}$ but not $Q_{X}^{-1}<D^{-1}$. Then, $\det(D^{-1}-Q_{X}^{-1})=0$. Hence,

$$\begin{array}{cc} \det(I-D^{1/2}Q_{X}^{-1}D^{1/2})= & \det(D^{1/2}\left[D^{-1}-Q_{X}^{-1}\right]D^{1/2})\\ = & \det\left(D^{1/2}\right)\det\left(D^{-1}-Q_{X}^{-1}\right)\det\left(D^{1/2}\right)=0. \end{array}$$

In this case, the inequality (A11) also holds.

(3) Then consider the case in which $D<Q_{x}<D^{-1}$. Lemma A1 will be used to prove the result. Define, therefore,

$$\begin{array}{ccc} A & = & \left(Q_{X}^{-1/2}\right)D^{1/2},\;\;B=\left(Q_{X}^{1/2}\right)D^{1/2}. \end{array}$$

First, it is proven that the assumptions of the lemma are satisfied. Note that $0<Q_{X}$ implies that $\mathrm{rank}(Q_{X})=n$. This and the fact that rank(D)=n imply that $\mathrm{rank}\left(B\right)=\mathrm{rank}\left(Q_{X}^{1/2}D^{1/2}\right)=n$. Further note that

$$\begin{array}{ccc} I-A^{T}A & = & I-D^{1/2}Q_{X}^{-1}D^{1/2},\\ & & 0<D\leq Q_{X}\leq D^{-1}\;by\;assumption,\;\Rightarrow \\ 0 & < & D\leq Q_{X}^{-1}\leq D^{-1},\;\;\;by\;Proposition,\\ 0 & < & D^{2}\leq D^{1/2}Q_{X}^{-1}D^{1/2}\leq I\;\Rightarrow \\ 0 & \leq & I-D^{1/2}Q_{X}^{-1}D^{1/2}=I-A^{T}A;\\ & & 0<D<Q_{X}<D^{-1}\;by\;the\;case\;considered,\\ 0 & < & D^{2}<D^{1/2}Q_{X}D^{1/2}<I,\;\Rightarrow \\ 0 & < & I-D^{1/2}Q_{X}D^{1/2}=I-B^{T}B;\\ I-A^{T}B & = & I-D^{1/2}Q_{X}^{-1/2}Q_{X}^{1/2}D^{1/2}=I-D. \end{array}$$

From Lemma A1 it then follows that

$$\begin{array}{ccc} & & \det\left(\left[I-D^{1/2}Q_{X}^{-1}D^{1/2}\right]\left[I-D^{1/2}Q_{X}D^{1/2}\right]\right)\leq \det\left({\left[I-D\right]}^{2}\right). \end{array}$$

(4) Then suppose that, in addition, $Q_{X}\neq I$. Then

$$\begin{array}{ccc} A-B & = & Q_{X}^{-1/2}D^{1/2}-Q_{X}^{1/2}D^{1/2}=Q_{x}^{-1/2}\left[I-Q_{X}\right]D^{1/2}\neq 0,\;usingthat,\;Q_{X}\neq I,\\ 0 & < & \left(A-B\right){\left[I-B^{T}B\right]}^{-1}{\left(A-B\right)}^{T},\;because\;0<I-B^{T}B,\;and\;A-B\neq 0,\\ & & \det(I-A^{T}A)\\ & < & \det\left(\left(A-B\right){\left[I-B^{T}B\right]}^{-1}\left(A-B\right)\right)+\det\left(I-A^{T}A\right),\\ & \leq & \det(\left(I-A^{T}B\right){\left[I-B^{T}B\right]}^{-1}{\left(I-A^{T}B\right)}^{T}),\;by\;Lemma,\\ & \Rightarrow & \det\left(\left(I-A^{T}A\right)\left(I-B^{T}B\right)\right)=\det\left(I-A^{T}A\right)\det\left(I-B^{T}B\right)\\ & < & {\left[\det\left(I-A^{T}B\right)\right]}^{2},\;by\;Lemma\;and\;its\;proof,\\ & \Rightarrow & \det\left(\left[I-D^{1/2}Q_{X}^{-1}D^{1/2}\right]\left[I-D^{1/2}Q_{X}D^{1/2}\right]\right)<\det\left({\left[I-D\right]}^{2}\right),\;by\;sub.of\;A,\;B. \end{array}$$

□
